# Which interactions matter in economic evaluations? A systematic review and simulation study

**DOI:** 10.1186/s12874-020-00978-0

**Published:** 2020-05-07

**Authors:** Helen Dakin, Alastair Gray

**Affiliations:** Health Economics Research Centre, Nuffield Department of Population Health, University of Oxford, Old Road Campus, Headington, Oxford, OX3 7LF UK

**Keywords:** Factorial randomised controlled trial, Economic evaluation, Cost-effectiveness analysis, Systematic review, Interactions, Simulation

## Abstract

**Background:**

We aimed to assess the magnitude of interactions in costs, quality-adjusted life-years (QALYs) and net benefits within a sample of published economic evaluations of factorial randomised controlled trials (RCTs), evaluate the impact that different analytical methods would have had on the results and compare the performance of different criteria for identifying which interactions should be taken into account.

**Methods:**

We conducted a systematic review of full economic evaluations conducted alongside factorial RCTs and reviewed the methods used in different studies, as well as the incidence, magnitude, statistical significance, and type of interactions observed within the trials. We developed the interaction-effect ratio as a measure of the magnitude of interactions relative to main effects. For those studies reporting sufficient data, we assessed whether changing the form of analysis to ignore or include interactions would have changed the conclusions. We evaluated how well different criteria for identifying which interactions should be taken into account in the analysis would perform in practice, using simulated data generated to match the summary statistics of the studies identified in the review.

**Results:**

Large interactions for economic endpoints occurred frequently within the 40 studies identified in the review, although interactions rarely changed the conclusions.

**Conclusions:**

Simulation work demonstrated that in analyses of factorial RCTs, taking account of all interactions or including interactions above a certain size (regardless of statistical significance) minimised the opportunity cost from adopting treatments that do not in fact have the highest true net benefit.

## Background

It has recently been suggested that many treatments are likely to have non-additive effects on costs and quality-adjusted life-years (QALYs), and that ignoring such interactions and making separate decisions on treatments which could in practice be used together may not achieve the best allocation of healthcare resources [1, 2]. Estimates of the incidence, magnitude and direction of interactions for economic endpoints are therefore required for decision-makers considering which interventions can be assessed independently and for researchers conducting factorial trials with economic evaluations or model-based economic evaluations on multiple treatments.

Factorial randomised controlled trials (RCTs) provide unbiased estimates of the magnitude of interactions. These studies randomise patients to different levels of at least two factors: for example, a 2 × 2 design may compare placebo, A, B and A + B. Taking account of interactions when analysing factorial trials avoids bias, but reduces statistical power, while omitting interaction terms and assuming that there is no interaction is more efficient, but introduces bias whenever the true interaction is non-zero [3–6]. In practice, researchers cannot know whether treatments genuinely interact and have only a single sample in which to decide which interactions matter and estimate treatment effects. Analysts must therefore pre-specify a decision rule or criterion that determines the circumstances in which interactions will be included in the base case analysis.

Analyses of primary clinical endpoints generally omit interactions that are not statistically significant [3, 4, 7]. Economic evaluation, however, focuses on estimating expected costs and benefits to inform decision-making, where statistical inference is arguably irrelevant [8]. It has therefore been suggested that it is important to avoid bias by including interactions in economic evaluations of factorial RCTs unless they are shown to be negligible [1]. However, even within this context there are several reasons to avoid conducting inefficient analyses. Firstly, inefficient analyses will over-estimate the value of further information, potentially displacing spending on healthcare today with over-investment in research. Secondly, small sample sizes or inefficient analyses may mean that (by chance) the treatment with highest expected net monetary benefit (NMB) in the sample being analysed is not the one that would genuinely maximise NMB in the population. However, it is not known what criteria achieve the best balance in minimising inefficiency and bias for economic evaluations.

This paper aims to assess the magnitude of interactions within a sample of published economic evaluations, evaluate the impact that different analytical methods would have had on the results and compare the performance of different criteria for identifying which interactions should be taken into account. We first conducted a systematic review of full economic evaluations conducted alongside factorial RCTs and reviewed the methods used in different studies and the incidence, magnitude, statistical significance, and type of interactions observed within the trials. As part of this review, we identified the existence of “mixed” interactions and developed the “interaction-effect ratio” as a measure of the magnitude and direction of interactions compared with main effects. For those studies reporting sufficient data, we assessed whether changing the form of analysis to ignore or include interactions would have changed the conclusions. We then evaluated how well different criteria for determining which interactions are considered in the analysis would perform in practice, using simulated data generated to match the summary statistics of the published examples.

## Systematic review

### Methods

A systematic review was conducted to identify studies for the simulation study. This aimed to identify all factorial RCTs with economic evaluations published before 2010 evaluating any intervention/comparator in any patient group. The protocol is available in Additional file 1. MEDLINE (including daily update and old MEDLINE), EMBASE, Econlit and Journals@Ovid were searched through Ovid on 9th February 2010. We also searched www.bmj.com, Tufts CEA registry (https://research.tufts-nemc.org/cear/Default.aspx), Wiley Interscience, National Institute for Health Research (NIHR) publications list (http://www.hta.ac.uk) and Centre for Reviews and Dissemination (CRD, http://www.crd.york.ac.uk/crdweb) Database on the same date. The review was not updated because the original review was sufficient to identify a representative sample of studies and provide the basis for the simulation study. The review followed PRISMA guidelines [9].

Search terms to identify factorial trials (e.g. “factorial”, “2 x 2”, “2 by 2”, “two by two”, or “2 x 3”) were combined with search terms to identify economic evaluations (“cost-effect*” or “economic evaluation” (See Additional file 1). Since some papers on factorial trial-based economic evaluations do not describe the design as factorial, clinical papers on factorial trials that happened to be picked up in the main database searches and which mentioned plans for an economic evaluation or collection of cost data were flagged. Additional targeted literature searches were then conducted to identify papers reporting economic evaluations of these specific factorial trials.

One author (HD) examined titles and abstracts to assess whether they met all of the following inclusion criteria:

- Described the methods and/or results of a cost-effectiveness, cost-utility, cost-consequence or cost-benefit analysis quantifying the costs and benefits of interventions designed to improve health or affect healthcare systems.
- Used patient- or cluster-level data from a factorial RCT, as defined in Additional file 1.
- Published at least brief details of the methods and/or results of the trial-based economic evaluation on/before 31st December 2009. Studies were not excluded from the review based on language, providing that at least an English abstract was available. For completeness, protocols published as journal articles by 31st December 2009 were also included, to give information on intended analytical methods.

The same author extracted data on study characteristics, study design, statistical methods and results (See Additional file 2). Mean costs and mean health benefits within each cell of the factorial design and their standard deviations were extracted if reported. These data were used in the simulation study and to estimate the magnitude, influence and (where possible) statistical significance of interactions.

Interactions were placed in one of four categories:

- super-additive: where the effect of the combination is greater than the sum of the parts;
- sub-additive: where the effect of the combination is less than the sum of the parts, but the interaction does not change the direction of effects;
- qualitative: where at least one of the treatments under investigation changes *sign* (not just magnitude) depending on whether or not the other therapy is given; and
- mixed: we developed the “mixed” category to reflect situations where one factor decreases outcome while the other increases it, such that the interaction has the same sign as one treatment effect, but the opposite sign from the other.

To measure the magnitude of interactions relative to between-group differences, we developed the ***interaction:effect ratio,***^1^ which indicates both the size of interactions and whether the interaction is super-additive, sub-additive/mixed or qualitative. The interaction:effect ratio (*IER*_*AB*_) equals the interaction term (*I*_*AB*_ = *μ*_0_ − *μ*_*a*_ − *μ*_*b*_ + *μ*_*ab*_) divided by the simple effect of A (*δ*_*A*_):

1 $$ {IER}_{AB}={I}_{AB}/{\delta}_A=\left({\mu}_0-{\mu}_a-{\mu}_b+{\mu}_{ab}\right)/\left({\mu}_a-{\mu}_0\right) $$

Simple effects comprise the difference in means between the group receiving one treatment and the group not receiving that treatment (*δ*_*A*_ = *μ*_*a*_ − *μ*_0_). When all treatments have the same direction of effect (e.g. when A and B both increase cost, or both decrease cost), the factor defined as A is the one for which the simple effects has the smaller absolute magnitude (where |*μ*_*a*_ − *μ*_0_| < |*μ*_*b*_ − *μ*_0_|). For mixed interactions, factor A should be the factor for which *δ*_*A*_ has the opposite sign to *I*_*AB*_. These rules ensures that qualitative interactions (those changing the ranking of treatments) have interaction:effect ratios <− 1. In all cases, interaction:effect ratios <− 1 indicate qualitative interactions, ratios between − 1 and 0 indicate sub-additive or mixed interactions, ratios equal to 0 indicate additive effects, while interaction:effect ratios > 0 indicate super-additive interactions.

### Results

**Fig. 1:**
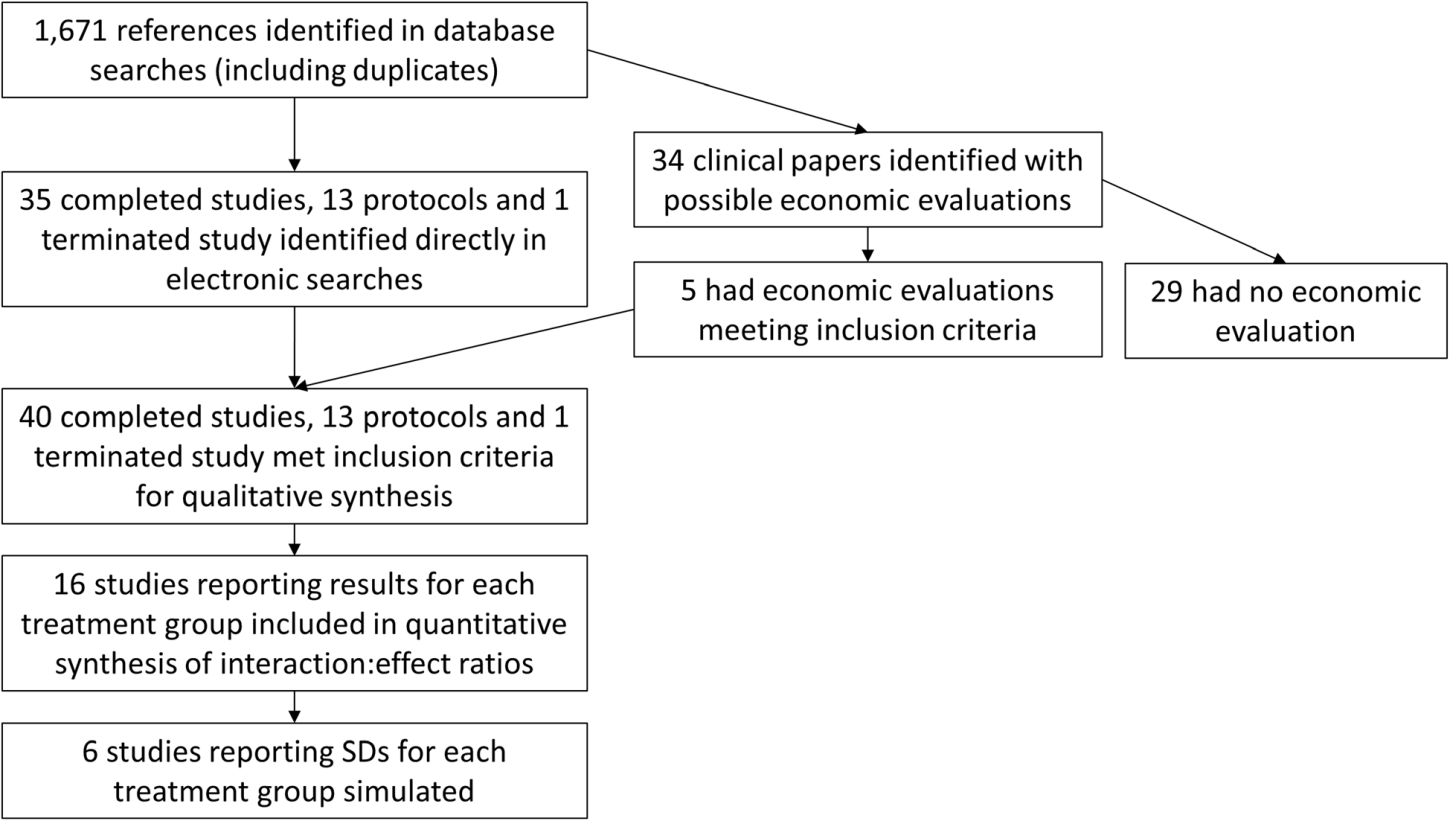
Flow diagram showing study identification

Searches identified 1671 references (Fig. 1, Additional files 1). Of these, 40 complete studies presenting economic evaluation results, 13 published protocols and one prematurely-terminated study^2^ met the inclusion criteria. Additional file 2 gives details of all included studies.

Of the completed studies, 23% (9/40) allowed for interactions between factors when analysing the primary clinical endpoint, 53% (21/40) assumed no interaction, while 25% (10/40) did not clearly state their methods (Table 1). Twenty studies (50%) used regression methods for the primary endpoint, of which five included interaction terms, seven did not and eight did not clearly describe their methods. Four studies used inside-the-table analysis and 14 used at-the-margins. Only three studies (8%) observed statistically significant interactions for the primary endpoint, although nine others (23%) observed large or qualitative interactions that did not reach statistical significance or for which significance was not reported. Interaction results were not clearly reported for 15 studies.

**Table 1:** Characteristics of the studies meeting inclusion criteria

			Completed studies	Protocols and terminated studies	Overall
Publication year^a^	2009		18% (7/40)	50% (7/14)	26% (14/54)
	2008		10% (4/40)	14% (2/14)	11% (6/54)
	2007		5% (2/40)	29% (4/14)	11% (6/54)
	2006		10% (4/40)	0% (0/14)	7% (4/54)
	2005		15% (6/40)	0% (0/14)	11% (6/54)
	2000–2004		28% (11/40)	7% (1/14)	6% (3/54)
	Pre-2000		15% (6/40)	0% (0/14)	9% (5/54)
Mainly/entirely publicly funded			75% (30/40)	100% (14/14)	81% (44/54)
Country where research done	United Kingdom		28% (11/40)	43% (6/14)	31% (17/54)
	United States		38% (15/40)	7% (1/14)	30% (16/54)
	Other individual country		23% (9/40)	29% (4/14)	24% (13/54)
	Multinational		13% (5/40)	21% (3/14)	15% (8/54)
Disease area	Cardiovascular disease		45% (18/40)	0% (0/14)	33% (18/54)
	Musculoskeletal		13% (5/40)	14% (2/14)	13% (7/54)
	Cancer		15% (6/40)	0% (0/40)	11% (6/54)
	Smoking, drugs or alcohol		13% (5/40)	29% (4/14)	17% (9/54)
	Other		25% (10/40)	57% (8/14)	33% (18/54)
Factors target different diseases			3% (1/40)	14% (2/14)	6% (3/54)
Type of intervention^b^	Pharmaceutical		45% (18/40)	36% (5/14)	43% (23/54)
	Physiotherapy, exercise and related		15% (6/40)	29% (4/14)	19% (10/54)
	Nutrition		8% (3/40)	14% (2/14)	9% (5/54)
	Surgery		13% (5/40)	7% (1/14)	11% (6/54)
	Training, counselling, incentives and logistical interventions		50% (20/40)	43% (6/14)	48% (26/54)
Sample size^c^	Mean (range)		3420 (126, 20,536)	1371 (132, 4733)	3002 (126, 20,536)
	< 200 patients		13% (5/39)	7% (1/14)	11% (6/53)
	200–999 patients		38% (15/39)	36% (5/14)	38% (20/53)
	> 1000 patients		49% (19/39)	29% (4/14)	43% (23/53)
Type of economic evaluation	Cost-effectiveness analysis		68% (27/40)	0% (0/14)	50% (27/54)
	Cost-effectiveness and cost-utility analysis		18% (7/40)	43% (6/14)	24% (13/54)
	Cost-utility analysis		8% (3/40)	29% (4/14)	13% (7/54)
	Cost-consequence analysis		8% (3/40)	0% (0/14)	6% (3/54)
	Cost-utility and cost-benefit analysis		0% (0/40)	7% (1/14)	2% (1/54)
	Depends on results		0% (0/40)	7% (1/14)	2% (1/54)
	Not stated		0% (0/40)	14% (2/14)	4% (2/54)
Cluster-randomised			13% (5/40)	43% (6/14)	20% (11/54)
Type of design	Full factorial		88% (35/40)	86% (12/14)	87% (47/54)
	Partial factorial		10% (4/40)	7% (1/14)	9% (5/54)
	Incomplete factorial		3% (1/40)	7% (1/14)	4% (2/54)
Factorial matrix size	2 factors & 2 levels2 (e.g. 2 × 2, 2 × 2 + 1, or double 2 × 2)		78% (31/40)	71% (10/14)	76% (41/54)
	> 2 factors each with 2 levels (e.g. 2x2x2)		10% (4/40)	0% (0/14)	7% (4/54)
	2 factors with > 2 levels on ≥1 factor (e.g. 3 × 2)		10% (4/40)	21% (3/14)	13% (7/54)
	> 2 factors with > 2 levels on ≥1 factor (e.g. 3x2x2)		3% (1/40)	7% (1/14)	4% (2/54)
Primary clinical endpoint	Assumed no interaction		53% (21/40)	29% (4/14)	46% (25/54)
	Included interaction term		23% (9/40)	7% (1/14)	19% (10/54)
	Unclear		25% (10/40)	64% (9/14)	35% (19/54)
Base case economic evaluation	Assumed no interaction		40% (16/40)	7% (1/14)	31% (17/54)
	Included interaction term		53% (21/40)	0% (0/14)	39% (21/54)
	Unclear		8% (3/40)	93% (13/14)	30% (16/54)
Presentation of uncertainty	None		48% (19/40)	–	48% (19/40)
	Present using confidence intervals and/or scatter graphs		15% (6/40)	–	15% (6/40)
	Cost-effectiveness acceptability curves	Pair-wise comparison(s)	23% (9/40)	–	23% (9/40)
		Multiple comparison(s)	8% (3/40)	–	8% (3/40)
		Pair-wise and multiple comparison(s)	8% (3/40)	–	8% (3/40)

^a^ Completed studies are categorised by the year when the first paper describing the results of the economic evaluation was published, while protocols are categorised by the date the protocol was published

^b^ Numbers add up to > 100% since 16 studies/protocols evaluated two or more different types of intervention

^c^ Excludes one cluster-randomised study for which the number of patients was not reported

By contrast, 53% (21/40) of completed studies allowed for interactions in their base case economic evaluation: more than twice the number allowing for interactions in the primary endpoint. Studies were also more likely to report sufficient information to identify whether interactions were taken into account for cost-effectiveness than primary endpoints, although in most cases it was necessary to infer the methods used from the tables reported. Only five studies analysed economic results using regression analyses, while two used event-based cost-effectiveness analysis, 17 inside-the-table and 14 at-the-margins; this may reflect the difficulties associated with regression-based economic evaluation identified previously [1].

Fifteen completed studies (38%) presented the probability of treatment being cost-effective within the text or as cost-effectiveness acceptability curves. Of these: nine studies presented pair-wise comparisons giving the probability that one treatment is cost-effective compared with a single comparator; three studies presented figures showing how the probability of each treatment evaluated in the trial having highest NMB varies with ceiling ratio; and a further three studies presented acceptability curves for both pair-wise and multiple comparisons. Six further studies quantified uncertainty in other ways (e.g. scatter graphs or confidence intervals). One study also presented the value of information [11–13].

Sixteen studies (40%) reported results inside-the-table in sufficient detail that interactions for both costs and health benefits could be directly evaluated (See Additional file 3).^3^ Large interactions arose frequently: 33% (24/72) of interactions had an absolute magnitude larger than one or more simple effect (interaction:effect ratios > 1 or < − 1; Table 2). Interaction:effect ratios varied between − 44 and 232. Overall, 33% of interactions were super-additive (23/72), 49% (35/72) were sub-additive or qualitative, while 17% (12/72) were mixed (Table 2). Large and qualitative interactions occurred at least as commonly for health benefits as for costs and NMB. Among the studies measuring health in units other than QALYs, 50% (7/14) of interactions were larger than simple effects. However, although 29% (7/24) of studies had qualitative interactions for NMB, the interaction changed the treatment adoption decision in only one case [15].

**Table 2:** Magnitude of interactions for the 16 studies reporting mean costs and mean health benefits for each cell within the factorial design

	Interaction: effect ratio	Proportion of interactions in different categories		
		Cost	Health benefit	Net monetary benefit^**a**^
Qualitative	<−1	16.7% (4/24)	33.3% (8/24)	29.2% (7/24)
Sub-additive	Between −1 and 0	41.7% (10/24)	25.0% (6/24)	25.0% (6/24)
Mixed non-qualitative^b^		4.2% (1/24)	12.5% (3/24)	12.5% (3/24)
Additive	0	0.0% (0/24)	8.3% (2/24)	0.0% (0/24)
Super-additive	> 0	37.5% (9/24)	20.8% (5/24)	33.3% (8/24)
Interaction:effect ratio > 1 or < −1		29% (7/24)	38% (9/24)	38% (9/24)
Range of interaction:effect ratios		-2, 232	−23, 2	−44, 13

^a^ A ceiling ratio of £20,000 (or $20,000 or €20,000) was used for quality-adjusted life-years (QALYs) [14] and life-years gained, while a ceiling ratio of £5000 (or $5000 or €5000) per unit of benefit was arbitrarily used for all other health benefits

^b^ Mixed interaction: one factor increases the outcome of interest, while the other decreases it. The interaction therefore cannot be classified as either sub-additive or super-additive

Six studies (reporting nine interactions) reported standard deviations around both costs and health benefits in each group [15–20]. Within these studies, 56% (5/9) of interactions for cost were statistically significant (*p* < 0.05), although there were no statistically significant interactions for health benefits or NMB.

## Simulation study

### Methods

The six studies reporting standard deviations for each group [15–20] were used in simulation work to evaluate the different criteria for identifying which interactions should be included in economic analyses. Using simulated data means that: (a) whereas for a real trial, we only see one sample, for simulated data, we can generate multiple samples and see how performance varies; (b) we specify the true data-generating mechanism and can compare the conclusions of each individual sample against the true answer; (c) we can vary the characteristics of the data-generating mechanism (e.g. interaction size and sample size) and see the impact on the results. For simplicity, simulations focused on balanced 2 × 2 full factorial designs with no covariates or missing data. We therefore only included the first two levels for each factor evaluated by Hollis et al. [20] and the Alexander Technique, Exercise And Massage (ATEAM) trial [17].

In addition to the original studies, five variants of each trial were simulated using interaction terms that were 0, 50% or 200% of the size observed in the original study, and using double the sample size with either the original interaction or zero interaction (See Additional file 3). The analysis used Stata version 12 (College Station, Texas) to simulate and analyse 300 samples of each of the 36 scenarios from the six trials. The data-generation methods and Stata code are shown in Additional file 3 and use the data in Additional file 5.

The costs and benefits for each sample were analysed using four mixed models with different combinations of interaction terms: no interactions; interaction for costs only; interaction for health benefits only; and interactions for costs and benefits. The mixed models implemented seemingly-unrelated regression allowing for correlations between costs and benefits by predicting outcomes (which could be either costs or benefits) with random effects by patient. However, separate constants, treatment effects and (where appropriate) interactions were estimated for costs and benefits and unstructured residuals were used. This approach gives identical results to the sureg command [21]. The log-likelihood, degrees of freedom and coefficients and their standard errors were recorded for each model.

The coefficients estimated in mixed models were used to calculate NMB. For simplicity, all costs were interpreted as though they were in pounds Sterling. Results focus on ceiling ratios of £20,000/QALY [14] for the five studies measuring benefits in QALYs, and £5000 per unit of benefit for other studies.

We evaluated 15 criteria for determining which interactions should be taken into account (Table 3) and applied these to each simulated trial sample. We compared the results of each analysis against the “true” results for each dataset, which (for the purposes of this simulation study) were assumed to equal the mean values for treatment effects and interactions shown in Additional file 3, Table 3.3 The sensitivity and specificity for identifying interactions, the probability of adopting the best treatment and the opportunity cost of making the wrong decision [1] were evaluated for each of the 15 criteria (Table 4).

**Table 3:** List of the criteria for determining which interactions are taken into account that were evaluated in the study

	Name of criterion	Rationale	Details of how it was applied
1	Always include all interactions	Sometimes referred to as “never pool” [3, 22]. Avoids bias, but has lower power unless interactions are very large [3–6]. May be particularly appropriate for economic evaluation [1] since this focuses on maximising expected net benefit subject to current information [8]. This approach is statistically consistent, in that we would always adopt the treatment that truly had the highest NMB if the sample size were infinite, although results may be more sensitive to chance than approaches excluding some interactions.	Interactions were included in analyses on all trial samples.
2	Never include any interactions	Sometimes referred to as “always pool” [3, 22]. Maximises statistical power unless interactions are very large, but is biased unless the true interaction is zero [3–6]. This approach is not statistically consistent and would cause us to adopt a suboptimal treatment whenever there is a qualitative interaction in NMB that changes which treatment had highest NMB, even with an infinite sample size.	No interactions were included analyses on any trial samples.
3	Include interactions where p < 0.05	Reflects standard practice for clinical endpoints, where only interactions that are statistically significant in an initial test are included in the main analysis [3, 4, 7]. Significance levels > 0.05 are sometimes used for the test on interactions [23]. However, most studies are underpowered for main effects in costs and QALYs [24–30], which are likely to have variances a quarter of the size of those found interaction terms [31–33]. Statistical inference may be irrelevant for decision-making, as health gains from the budget are maximised by adopting the treatment with the highest expected net benefit [8].	Interaction for cost [or benefits] was included if it was statistically significantly different from zero in the model that included interactions for both costs and benefits.
4	Include interactions where *p* < 0.10		
5	Include interactions where *p* < 0.25		
6	Include interactions decreasing AIC	Information criteria trade efficiency against bias, taking account of sample size [34], although this trade-off is based on information theory, rather than decision analysis. These measures have been used outside of healthcare to decide whether to include interactions in factorial experiments [35].	Results are based on the mixed model with lowest AIC/BIC.
7	Include interactions decreasing BIC		
8	Include qualitative interactions in cost or benefits	Interactions that change the ranking of treatments for cost or benefits may also have a high chance of changing the ranking of treatments for net benefits and therefore could also change the conclusions. This approach is simpler to implement than the criteria based on interactions for net benefit as it does not depend on the ceiling ratio. However, at ceiling ratios other than zero and infinity, the conclusions of economic evaluation could be sensitive to interactions even if this criterion does not pick up qualitative interactions for either costs or benefits.	Includes interactions for cost [benefits] that change rankings of treatments for cost [benefits]: i.e. those that are larger than and have the opposite sign from one or both of the simple effects (which will have interaction:effect ratios <− 1).
9	Include interactions for cost or benefits if >simple effect	This criterion includes super-additive interactions for cost or benefits that are larger than as the smaller of the two simple effects, as well as the qualitative interactions included in criterion 8. However, like 8, it may not identify all qualitative interactions for net benefit.	All interactions with an absolute magnitude larger than the smaller of the two simple effects (i.e. all those with interaction:effect ratios <− 1 or > 1) are included.
10	Include interactions for cost or benefits if p < 0.05 or > simple effect	This approach takes account of statistical significance and interactions that are larger than main effects.	As for 9, but also including smaller interactions that are statistically significantly different from zero.
11	Include qualitative interactions for cost, benefits or NMB	Allowing for interactions will have no effect on the conclusions about which treatment is adopted unless the interactions are qualitative on a NMB scale (i.e. change the ranking of treatments) at the ceiling ratio(s) of interest. However, since the true shadow price of a QALY is unknown, this approach requires arbitrary judgements about the ceiling ratio(s) at which the interactions are assessed. Including all interactions that are qualitative at any ceiling ratio would generally result in inclusion of all interactions, since any quantitative interaction in either costs or QALYs will produce a qualitative interaction in NMB at some ceiling ratio whenever the treatment lies in the north-east or south-west quadrants [1].	Interactions were calculated at a series of 8 evenly-spaced ceiling ratios between £5000 and £40,000 per unit benefit using the coefficients for all 4 mixed models. Interactions for costs [or benefits] were included if the interaction for cost [or benefits] was qualitative, or if the coefficients from the mixed model that included an interaction for cost [or benefits] but not benefits [or cost] produced a qualitative interaction for NMB at any of the ceiling ratios.
12	Include interactions for cost, benefit or NMB if >simple effect		Includes all qualitative interactions in cost, benefits or NMB and any super-additive interactions that are larger than the smaller of the two simple effects. Calculated as for 11, but also including large super-additive interactions.
13	Include |interactions| ≥0.25 or ≥ £250	An absolute limit for the size of interaction that can safely be ignored could be pre-specified. However, there is no general rule for how large this limit should be and it may vary between applications. The size thresholds used were chosen arbitrarily.	Only interactions above the designated size threshold were taken into account. For example, criterion 13 includes interactions in benefits that are ≥0.25 (or ≤ −0.25) units in size and interactions in cost that are ≥£250 (or ≤ −£250) in size.
14	Include |interactions| ≥0.5 or ≥ £500		
15	Include |interactions| ≥1 or ≥ £1000		

*Abbreviations: AIC* Akaike information criterion, *BIC* Bayesian information criterion, *NMB* net monetary benefit, *QALY* quality-adjusted life-year

**Table 4:** The measures used to assess performance of the criteria for deciding which interactions are considered

Measure	Rationale	Details of how it was calculated
Sensitivity for including non-zero interactions	Sensitivity and specificity evaluate the extent to which criteria identify non-zero interactions, but do not reflect the consequences of ignoring them.	The proportion of samples in which interactions in cost [or benefit] were taken into account in the analysis when the true interaction was not zero
Specificity for excluding interactions equal to zero		The proportion of samples in which interactions in cost [or benefit] were excluded from the analysis when the true interaction equalled zero
Probability of adopting treatment with highest NMB	This focuses on the purpose of economic evaluation: namely to inform a treatment adoption decision regarding which treatment has highest expected NMB and to thereby maximise health gain from the budget. It assumes that inference is irrelevant to decision-making [8], but nonetheless acknowledges that inefficient analysis and small sample sizes may cause us to adopt the wrong treatment by chance. The probability of making the wrong decision may be relevant risk-averse decision-makers. However, it does not take account of the consequences of making the wrong decision.	The treatment arm with highest expected NMB was identified at the ceiling ratio of interest for (a) the “true” parameters used to generate the data and (b) based on the mixed model coefficients estimated on each sample. The proportion of samples in which the treatment predicted to have highest NMB (b) was the same as the “true” best treatment (a) was calculated for each scenario.
Opportunity cost associated with adopting a suboptimal treatment	This measure takes account of the opportunity cost of adopting the wrong treatment, as well as the probability of adopting the wrong treatment [1]. It is similar to the opportunity cost of ignoring interactions [1] but is based on a contrast between the genuine best treatment and the treatment predicted to be best, rather than a comparison between two imperfect analyses on finite samples. As such, the opportunity cost estimated here takes account of situations where allowing for spurious interactions causes us to adopt the wrong treatment by chance, as well as situations where ignoring interactions biases the analysis.	For each sample, the opportunity cost was defined as the NMB for the “true” best treatment (a) minus the NMB for the treatment predicted to have highest NMB in that analysis of that sample (b). In both cases, NMB for each treatment was calculated using the “true” parameters used for data generation. Opportunity cost was therefore zero for all samples in which the “true” best treatment was adopted and positive in all other cases. Opportunity cost was then averaged across samples and scenarios.

*Abbreviations: NMB* net monetary benefit

We used the opportunity cost as the primary measure of which criterion works best, since it focuses on the central question of economic evaluation: namely maximising health gains from a finite budget. Coverage, statistical power and bias were also calculated (Additional file 4).

### Results

The 15 criteria differed in the proportion and type of interactions that were correctly identified (Table 5). Other than the “always include interactions” criterion (criterion 1), including interactions where *p* < 0.25 (criterion 5) and including interactions that are statistically significant or greater than simple effects (criterion 10) resulted in the largest number of cost interactions being included. By contrast, criteria 5 and 9–12 included the largest number of benefit interactions. In general, specificity and sensitivity were inversely proportional; measures based on information criteria or statistical significance at alpha = 0.05 tended to have high specificity and low sensitivity.

**Table 5:** Comparison of performance of the different criteria with regards the probability and the opportunity cost associated with adopting a treatment that does not have highest true NMB. The values shown in bold represent the most favourable of all criteria for this measure

Criterion	Proportion of samples in which interactions are included: % (n)^**a**^	Sensitivity: proportion of any non-zero interactions taken into account: % (n)^**b**^	Specificity: proportion of interactions equal to 0 that are excluded: % (n)^**c**^	Mean opportunity cost of adopting a suboptimal treatment	Probability of adopting treatment with highest NMB
1: Always include all interactions	100.00% (21,600)	**100.00% (14,400)**	0.00% (0)	£472	81.70%
2: Never include any interactions	0.00% (0)	0.00% (0)	**100.00% (7200)**	£793	76.60%
3: Include interactions where p < 0.05	34.44% (7439)	46.59% (6709)	89.86% (6470)	£556	80.61%
4: Include interactions where p < 0.10	44.86% (9689)	56.87% (8189)	79.17% (5700)	£515	81.32%
5: Include interactions where p < 0.25	68.45% (14,785)	77.18% (11,114)	49.01% (3529)	£491	81.58%
6: Include interactions decreasing AIC	40.74% (8800)	52.91% (7619)	83.60% (6019)	£529	81.11%
7: Include interactions decreasing BIC	19.79% (4275)	28.88% (4158)	98.38% (7083)	£646	78.32%
8: Include qualitative interactions in cost or benefits	42.02% (9076)	47.54% (6846)	69.03% (4970)	£503	81.71%
9: Include interactions for cost or benefits if >simple effect	56.04% (12,104)	63.77% (9183)	59.43% (4279)	£474	**83.31%**
10: Include interactions for cost or benefits if p < 0.05 or > simple effect	61.78% (13,344)	71.42% (10,285)	57.51% (4141)	£475	83.14%
11: Include qualitative interactions for cost, benefits or NMB	57.01% (12,314)	60.76% (8749)	50.49% (3635)	£480	81.21%
12: Include interactions for cost, benefit or NMB if >simple effect	67.85% (14,656)	73.40% (10,569)	43.24% (3113)	£475	81.55%
13: Include interactions ≥0.25 or ≥ £250	31.92% (6894)	37.02% (5331)	78.29% (5637)	**£472**	81.22%
14: Include interactions ≥0.5 or ≥ £500	21.73% (4694)	24.69% (3555)	84.18% (6061)	£476	82.33%
15: Include interactions ≥1 or ≥ £1000	16.65% (3596)	18.51% (2666)	87.08% (6270)	£488	82.01%

*Abbreviations: AIC* Akaike information criterion, *BIC* Bayesian information criterion, *NMB* net monetary benefit, *QALY* quality-adjusted life-year

^a^ Percentages are out of 21,600 (300 samples of 6 trials, each with six scenarios across two endpoints [costs and QALYs])

^b^ Percentages are out of 14,400 (300 samples of 6 trials, each with four scenarios with non-zero interactions across two endpoints [costs and QALYs])

^c^ Percentages are out of 7200 (300 samples of 6 trials, each with two scenarios with zero interactions across two endpoints [costs and QALYs])

Averaging across all 36 scenarios from the six trials, including interactions ≥0.25 or ≥ £250 minimised the opportunity cost from adopting treatments that do not in fact maximise true NMB, while the opportunity cost of “always include interactions” was £0.04 larger (Table 5). “Never include interactions” performed worst, while criteria 3–7 (based on statistical significance and information criteria) also performed poorly.

However, the criterion with lowest opportunity cost differed between individual scenarios (See Additional file 4). As expected, “never include interactions” was, *on average*, the best criterion for the scenarios that did not have qualitative interactions, although no criteria had high opportunity costs when interactions were zero. Across the 13 scenarios with qualitative interactions, “always include interactions” performed best, although criteria 11–13 also performed well (including qualitative interactions, including interactions >simple effects or including interactions ≥0.25 or ≥ £250).

Across all scenarios, criterion 9 (including interactions >simple effects) had the highest probability of adopting the treatment that has highest true NMB (Table 5). “Never include interactions” performed worst overall on this measure, but performed best in scenarios without qualitative interactions for NMB. “Always include interactions” performed best when there were qualitative interactions. However, results differed substantially between scenarios (not shown).

Doubling the sample size reduced the opportunity cost and the probability of adopting the wrong treatment for all criteria. However, criteria based on statistical significance or information criteria (which explicitly take account of sample size) did not appear to perform any better relative to other criteria in larger studies. Furthermore, criterion 11 (including qualitative interactions for cost., benefits or NMB) performed best in scenarios with double the original sample size, whereas “always include interactions” performed best with a smaller sample size.

Including all interactions was also the only criterion for which the 95% confidence intervals gave 95% coverage and also had no bias (See Additional file 4). Excluding all interactions had lowest coverage and highest bias. Including all interactions had lowest statistical power, while criteria 2, 8, 14 and 15 had highest statistical power (never include interactions, include qualitative interactions, include interactions ≥0.5 or ≥ £500 and include interactions ≥1 or ≥ £1000).

## Discussion

Between-treatment interactions that can change the treatment adoption decision need to be taken into account in healthcare decision-making, model-based economic evaluations and economic evaluations based on factorial RCTs [1, 2]. However, to our knowledge, this is the first study to evaluate the magnitude of interactions within published economic evaluations or compare different criteria for determining which interactions should be included in economic analysis.

This systematic review found that 26% of all interactions in factorial trial-based economic evaluations published before 2010 were qualitative (i.e. change the ranking of treatments and render at-the-margins estimates misleading [5, 36]), although interactions changed the treatment adoption decision in only one study. This provides empirical evidence on the importance of taking account of interactions within economic evaluations based on factorial trials [1] and within decision-analytical models and health technology assessment [2]. Our results may also be useful for researchers defining informative priors for Bayesian analyses: one previous study assumed that the probability of a qualitative interaction is just 2.5% [12]: less than a tenth of the frequency that we observed in our review.

However, 60% of studies did not report mean costs and benefits for each group inside-the-table; such presentation is important to allow readers to assess the impact of interactions and the extent to which they may bias the results [1]. Furthermore, the 16 studies reporting costs and benefits inside-the-table may not be typical: studies may have reported results inside-the-table *because* interactions were large. Of the completed studies, 53% allowed for interactions in their base case economic evaluation, whereas only 23% considered interactions for the primary clinical endpoint; these figures are similar to those reported previously [10, 37]. The higher figure for economic evaluations could be due to interactions being smaller for the primary clinical analysis than the endpoint used in economic evaluation, or interactions being smaller when analysed on the logarithmic scale, which may be appropriate for many clinical endpoints but not economic evaluation [1]. Alternatively, the greater use of inside-the-table analysis within economic evaluation could reflect economic thinking: particularly the view that inference is irrelevant [8], or that treatment-combinations should be evaluated as mutually-exclusive alternatives.

Our review aimed to assess the magnitude of interactions in a representative sample of studies and provide data inputs for simulation work. Our literature search was conducted in 2010 and a separate systematic review of economic evaluations of factorial trials conducted in 2013 identified seven studies published since our search date but used a different search strategy [37]. However, there is no reason to expect the incidence of interactions or the performance of different criteria to have changed over time. Systematic identification of factorial trials is hindered by the absence of a medical subject heading (MeSH) term specific to this type of design. Our review may therefore have missed studies that did not mention the factorial design in the abstract, particularly if they presented results for only one factor; as result, the review may underestimate the proportion of studies that have ignored interactions. However, our literature searches nonetheless identified four times as many pre-2010 papers than the review by Frempong et al. [37]: probably by using more general search terms, which yielded 10 times as many hits in bibliographic databases.

In four studies [38–41], the interaction between factors was confounded as the treatment given to the *ab* group was not equal to the sum of the treatments given to the *a* and *b* groups.^4^ Giving an additional intervention (e.g. advice or training) to the control group or to the three active treatment groups or varying how treatment is administered means that all estimates of the AB interaction are confounded by differences in treatment and makes analyses ignoring any interactions questionable. Future studies should avoid such confounding. If it is essential to give an additional treatment (e.g. for ethical reasons), papers should justify this decision and discuss what effect this is likely to have had on outcomes and interactions and (arguably) should not describe the study as factorial if the additional treatment is likely to influence outcomes.

Across all 36 scenarios, strategies of including all interactions, or including interactions larger than an arbitrary but relatively low threshold minimised the average opportunity cost associated with adopting the wrong treatment. Excluding all interactions, or using information criteria or statistical significance generally performed poorly on the measures most relevant to economic evaluation. However, the best criterion depended on how criteria were evaluated. For example, the probability of adopting the treatment with the highest NMB was slightly higher for criterion 9 (including interactions larger than simple effects) than for “always include interactions”. There were also substantial variations in the relative performance of different criteria between trials and scenarios. In particular, criteria that excluded most interactions performed well in scenarios where interactions equalled zero or did not change the ranking of treatments. The performance of different criteria varied little with sample size and the best-performing criteria take no account of sample size, suggesting that avoiding bias is more important than avoiding inefficiency even when sample size is limited.

The simulation study was based on six factorial trials and a small range of variants on each study. Since the most appropriate criterion differs between studies, different results could have been obtained with a different set of trials or scenarios. The analysis focused on 2 × 2 full factorial trials and interactions between two factors. Although the same principles are likely to apply to larger factorial designs, higher-order interactions between three or more treatments may be harder to detect. Furthermore, all trials were simulated and analysed as though they measured health benefits on continuous scales and all costs and health benefits were analysed on a natural scale using arbitrary ceiling ratios. The data-generating mechanism also simulated trials with complete, uncensored data, equal numbers in each arm, gamma-distributed costs with predictable patterns of heteroskedasticity and Gaussian, homoskedastic health benefits. Interactions were assumed to affect all patients in the A + B group equally (which may not be the case for rare events). Mixed models and the criteria based on statistical significance may perform less well in real trials where these idealised data characteristics do not apply. The optimal choice of criteria may also be sensitive to these features common to all simulated datasets (see Additional file 3).

## Conclusions

Large and qualitative interactions occur relatively commonly for costs, QALYs and net benefits. Future systematic review updates may help assess whether the conduct of economic evaluations of factorial trials has changed and quantify interactions in a wider sample of trials.

The simulation study demonstrated that it is better to include interactions that may have arisen by chance than risk ignoring genuine interactions that could change the conclusions. Researchers planning an economic evaluation based on a factorial trial should pre-specify and justify the criterion used to determine which interactions will be taken into account in the base case analysis [1]: e.g. in a health economics analysis plan [42]. The chosen criterion should balance the risk of bias from ignoring interactions against the loss of power from including interactions and the risk of drawing the wrong conclusions by chance. Although the criteria that performed best in our study depended on the magnitude of the true interaction, minimising the risk of bias by including all interactions or excluding only small/quantitative interactions tended to perform best. Criteria relying on statistical significance or information criteria performed poorly. This differs from the approach currently used by statisticians, although at least one published economic evaluation has used a pre-specified rule that interactions larger than main effects would be taken into account [43]. Any prior evidence or beliefs about the size of interactions could be used to select the appropriate criteria or as informative priors in a Bayesian analysis. In particular, a strategy of including all interactions above a certain size may perform better if the threshold is based on the expected treatment effects or the amount of bias that is acceptable in a particular setting. In addition to the criteria considered here, researchers could exclude all interactions not hypothesised a priori, or those that do not have plausible biological explanations. Whenever the base case analysis excludes any interactions, researchers should always present a sensitivity analysis including all interactions to assess the risk of bias [1].

## Supplementary information

**Additional file 1.** Protocol for the systematic review. Includes search strings and numbers of hits.

**Additional file 2.** Data extraction table for the systematic review of studies conducting economic evaluations of factorial design studies. Includes full details on each study meeting inclusion criteria.

**Additional file 3.** Additional methods on data simulation and analysis. Includes data on the magnitude of interactions for each of the studies reporting mean costs and mean health benefits for each cell within the factorial design, data inputs used in the simulation study and the Stata code used for statistical analysis.

**Additional file 4.** Additional results of the simulation study. Presents data on the performance of different criteria in different types of scenario and for each individual scenario of each trial, as well as data on bias and coverage.

**Additional file 5.** Data inputs used in the simulation study. Data are provided in a format that is imported by the Stata code given in Additional file 3.

## Data Availability

The datasets supporting the conclusions of this article are included within the article and its additional files. The data extraction table for the systematic review is available in Additional file 2. The data and Stata code used in the simulation study are available in Additional files 3 and 5.

## Abbreviations

ATEAM: Alexander Technique, Exercise and Massage [trial]
MeSH: medical subject heading
NIHR: National Institute for Health Research
NMB: net monetary benefit
QALY: quality-adjusted life-year
RCT: randomised controlled trial

## Footnotes

1 The interaction:effect ratio differs from the “interaction ratio” used by McAlister et al. [10]. McAlister’s interaction ratio is simply the relative effect (e.g. odds ratio) of A vs. not-A for patients also receiving B, divided by the relative effect of A vs. not-A for patients not receiving B (interaction ratio = (*odds*_*ab*_/*odds*_*b*_) ÷ (*odds*_*a*_/*odds*_0_)) and therefore equals the interaction on a logarithmic scale (interaction on log-scale = exp[(ln[*odds*_*ab*_] − ln[*odds*_*b*_]) − (ln[*odds*_*a*_] − ln[*odds*_0_])]). Unlike our interaction:effect ratio, McAlister’s interaction ratio is appropriate only for data interpreted on a multiplicative scale and does not distinguish between qualitative and non-qualitative interactions. At least one previous paper has used the interaction divided by simple effect to describe the ranges of interaction magnitude in which different analytical approaches performed best [3]. However, this study did not include any adjustment for mixed interactions, did not link the ranges of ratio values with different types of interaction and did not propose this ratio as a method for describing interactions in general.

2 One study meeting inclusion criteria was terminated early due to poor recruitment but was published as a monograph without analysis of economic results; this is considered in the review alongside protocols.

3 Since four of these studies were larger than 2 × 2 or reported results by subgroup, this gave 24 interactions for each of three outcomes (cost, QALYs and NMB): 72 interactions in total.

4 Three of these studies were included in the review as the authors described them as factorial [38–40]. In one study [40], general practitioners randomised to one of the three active treatment groups received a training session not given to the control group. Conversely, two trials gave patients in the control group an additional intervention not given to the other three groups. A fourth trial, in which the second factor compared physiotherapy against reinforcement of the advice given as part of factor 1 (a whiplash book or usual advice), was excluded from the review as the authors did not describe it as factorial [41]. A further study that was included in the review allowed information sharing between practitioners within the *ab* group that was not possible within the groups receiving < 2 interventions [19].
