# Supplementary material for: Which interactions matter in economic evaluations? A systematic review and simulation study
Source: BMC Med Res Methodol. 2020 May 7;20:109. doi: 10.1186/s12874-020-00978-0 (PMC7203889; doi:10.1186/s12874-020-00978-0)
Supplement: Supplementary file 1 — Additional file 1. Protocol for the systematic review. Includes search strings and numbers of hits. [file 12874_2020_978_MOESM1_ESM.docx]

**Additional file 1: Protocol for the systematic review**

**Aims**

- To identify all examples of full economic evaluations conducted on patient-level data from factorial design RCTs that were published before 31^st^ December 2009.
- To review the characteristics, study design and statistical methods used in these studies, identify any issues raised by their conduct or results, assess the strengths and weaknesses of each approach and identify data suitable for the simulation study.

**Inclusion criteria**

- Described the methods and/or results of a full economic evaluation quantifying the costs *and* benefits of interventions designed to improve health or affect healthcare systems. Cost-effectiveness analyses (CEA), cost-utility analyses (CUA), cost-consequence analyses (CCA) and cost-benefit analyses (CBA) were classed as full economic evaluations.
  - Cost minimisation analyses (CMAs) and costing analyses were excluded as they consider only one dimension of outcome (i.e. costs but not benefits) and therefore do not raise the same issues as full economic evaluations that consider both costs and outcomes.
  - Although the primary focus was on analyses estimating incremental cost-effectiveness ratios, CBAs of factorial design studies were included (although it was considered highly unlikely that any such studies would be identified).
  - CCAs were included since some such studies do calculate cost-effectiveness ratios and therefore raise similar issues to CEAs and CUAs.
- Used patient-level data from a factorial RCT.
  - Studies with individual-level randomisation were only included if they used patient-level data (as opposed to model-based economic evaluations based on secondary data from factorial trials). However, we also included economic evaluations on cluster-randomised trials that collected only one set of data on costs and/or benefits for each *cluster* (e.g. measuring cost per general practice, rather than per patient).
  - Based on a conceptual literature review [1], factorial RCTs were defined as studies in which two or more factors (each with two or more levels) are deliberately varied at the same time, with different groups of participants and/or centres being randomly allocated to receive different combinations of levels for each factor in such a way that interactions between two or more factors can be evaluated. Three-arm trials (e.g. studies comparing 0 vs. A vs. B, or A vs. B vs. A+B) were excluded from the systematic review since they do not permit evaluation of interactions. However, incomplete or fractional factorial design studies of larger or higher-order designs and partial factorial designs were included. Studies in which the interaction between factors was, to some extent, confounded by treating the group assigned to no treatment or combination therapy differently were included only if the authors described their study as factorial.
- Published at least brief details of the methods and/or results of the factorial trial-based economic evaluation before 31^st^ December 2009.
  - Full protocols of studies for which results were not yet available but for which a full economic evaluation was planned were included in the review if they were published in a journal article. Protocols available only on the NIHR HTA website were excluded. Protocols that mentioned collection of costs as well as clinical outcomes but did not explicitly discuss assessment of cost-effectiveness were excluded.
  - Papers that described the clinical results of a factorial study and stated that a full economic evaluation was planned were identified but were not included in the count of studies meeting inclusion criteria unless the economic paper was subsequently identified. Basic details on these studies were extracted into a separate table to aid identification of the full economic evaluation. Additional targeted literature searches were conducted to find the economic evaluations to which these clinical papers referred (see below). However, no specific searches were conducted to identify clinical papers of this type and papers describing studies with economic evaluations described elsewhere were only identified if they were picked up by the main literature searches AND if the paragraph(s) that included the term “cost-effect*” or “economic” explicitly mentioned that an economic evaluation was planned (as opposed to simply making general statements that “cost-effectiveness of X should be assessed” or “X may be cost-effective”). Clinical trials identified in searches were also flagged and economic papers were sought if the reviewer (HD) already knew that an economic evaluation had been done.

**Exclusion criteria**

- Studies were not excluded from the review on the basis of language, providing that at least some text (e.g. an abstract) was available in English.
- Since the search strategy focussed on databases of studies relevant to medicine and the focus of this thesis is on economic evaluation of healthcare interventions, studies unrelated to health or healthcare were excluded.

**Search strategy**

**Table 1.1** Search strategy for the conceptual review on issues and methods for factorial design trials

| **Database** | **Search terms for study design** | | |  | **Search terms for economic evaluation** |
| --- | --- | --- | --- | --- | --- |
| - MEDLINE (Ovid) - EMBASE (Ovid) - Econlit (Ovid) - Journals@Ovid full textx - Bmj.com (full text of all Highwire journals) - Sciencedirect.com | - Factorial | | | AND | cost-effect* OR economic  [in either title or abstract (or key words if available)] |
|  | - - “2x2”   - “2 x 2”   - “2 by 2”   - “two x two”   - “two by two”   - “2 x 3”   - “3 x 3”   - “3 x 2”   - “2 x 2 x 2” | | |  |  |
|  | - - Matrix | AND | Randomised OR randomized |  |  |
| - CRD (NHS EED and HTA only) - Tufts CEA registry (<https://research.tufts-nemc.org/cear/Default.aspx>) - NIHR HTA publications list (<http://www.hta.ac.uk/>) | - Factorial   - “2x2”   - “2 x 2”   - “2 by 2”   - “two x two”   - “two by two”   - “2 x 3”   - “3 x 3”   - “3 x 2”   - “2 x 2 x 2”   - Interaction [not used for searches of CRD due to excessive number of irrelevant hits] | | | - | - |

In cases where the primary literature searches identified a clinical paper that explicitly mentioned in the abstract (or in the paragraph(s) of the paper that used the words “cost-effect*” or “economic”) that a full economic evaluation was planned, additional searches were conducted to identify the paper that described the economic evaluation. In the first instance, we searched Medline using EITHER the study name/acronym, OR the generic name of one of the treatments used, the name of the disease and “cost” as search terms. If this did not identify the study, searches were repeated without the name of the disease and with the word “economic” used in place of “cost” and the studies citing the clinical paper were then reviewed. If the study was not identified on this basis, it was assumed that the economic evaluation was not yet published or that the authors had decided not to conduct an economic evaluation and the study was excluded from the review.

The full text of studies identified in electronic searches that did not include an abstract was only examined in cases where the available details (e.g. title, authors and/or journal) suggested that the article was likely to meet the inclusion criteria. In particular, full text was not sought for articles with titles that made reference to a clinical trial but not to any economic analysis unless the abstract, authors or journal suggested that an economic evaluation was indeed conducted.

**Data extraction**

Studies were identified and extracted by one reviewer (HD). The analysis of interactions and the simulation study were not pre-specified and were planned after the systematic review searches and data extraction were completed.

**Results of searches**

**Table 1.2** Results of searches

| **Website** | **Databases searched** | **Terms searched** | | **Hits** |
| --- | --- | --- | --- | --- |
| Ovid | Journals@Ovid, Econlit, EMBASE, Ovid MEDLINE(R), Ovid MEDLINE(R) Daily Update, Ovid OLDMEDLINE(R)... | factorial.af. and cost-effect*.ab. | | 296 |
|  |  | factorial.af. and cost-effect*.ti. | | 61 |
|  |  | factorial.af. and economic evaluation*.ti. | | 10 |
|  |  | factorial.af. and economic evaluation.ab. | | 19 |
|  |  | "2x2".af. and cost-effect*.ab. | | 41 |
|  |  | "2x2".af. and cost-effect*.ti. | | 9 |
|  |  | "2x2".af. and economic evaluation*.ti. | | 1 |
|  |  | "2x2".af. and economic evaluation*.ab | | 1 |
|  |  | "2 x 2".af. and cost-effect*.ab. | | 51 |
|  |  | "2 x 2".af. and cost-effect*.ti | | 17 |
|  |  | "2 x 2".af. and economic evaluation*.ab | | 4 |
|  |  | "2 x 2".af. and economic evaluation*.ti | | 4 |
|  |  | "2 by 2".af. and cost-effect*.ab. | | 382 |
|  |  | "2 by 2".af. and cost-effect*.ti | | 95 |
|  |  | "2 by 2".af. and economic evaluation*.ab | | 24 |
|  |  | "2 by 2".af. and economic evaluation*.ti | | 16 |
|  |  | "two x two".af. and cost-effect*.ab. | | 0 |
|  |  | "two x two".af. and cost-effect*.ti | | 0 |
|  |  | "two x two".af. and economic evaluation*.ab | | 0 |
|  |  | "two x two".af. and economic evaluation*.ti | | 0 |
|  |  | "two by two".af. and cost-effect*.ab. and trial.af | | 33 |
|  |  | "two by two".af. and cost-effect*.ti. and trial.af | | 9 |
|  |  | "two by two".af. and economic evaluation*.ab. and trial.af | | 2 |
|  |  | "two by two".af. and economic evaluation*.ti. and trial.af | | 0 |
|  |  | "two by two".af. and cost-effect*.ab. | | 46 |
|  |  | "two by two".af. and cost-effect*.ti. | | 11 |
|  |  | "two by two".af. and economic evaluation*.ab. | | 7 |
|  |  | "two by two".af. and economic evaluation*.ti. | | 1 |
|  |  | "2 x 3".af. and cost-effect*.ab. and trial.af | | 1 |
|  |  | "2 x 3".af. and cost-effect*.ti. and trial.af | | 0 |
|  |  | "2 x 3".af. and economic evaluation.ab. and trial.af | | 0 |
|  |  | "2 x 3".af. and economic evaluation.ti. and trial.af | | 0 |
|  |  | "2 x 3".af. and cost-effect*.ab | | 4 |
|  |  | "2 x 3".af. and cost-effect*.ti. | | 0 |
|  |  | "2 x 3".af. and economic evaluation.ab. | | 0 |
|  |  | "2 x 3".af. and economic evaluation.ti | | 0 |
|  |  | "3 x 3".af. and cost-effect*.ab. | | 5 |
|  |  | "3 x 3".af. and cost-effect*.ti. | | 0 |
|  |  | "3 x 3".af. and economic evaluation.ab. | | 0 |
|  |  | "3 x 3".af. and economic evaluation.ti | | 0 |
|  |  | "3 x 2".af. and cost-effect*.ab. | | 7 |
|  |  | "3 x 2".af. and cost-effect*.ti | | 0 |
|  |  | "3 x 2".af. and economic evaluation.ab. | | 0 |
|  |  | "3 x 2".af. and economic evaluation.ti. | | 0 |
|  |  | "2 x 2 x 2".af. and cost-effect*.ab. | | 1 |
|  |  | "2 x 2 x 2".af. and cost-effect*.ti. | | 0 |
|  |  | "2 x 2 x 2".af. and economic evaluation.ab. | | 0 |
|  |  | "2 x 2 x 2".af. and economic evaluation.ti. | | 0 |
|  |  | Matrix.af. and cost-effect*.ab. and randomized.ab | | 79† |
|  |  | Matrix.af. and cost-effect*.ab. and randomised.ab | | 25† |
|  |  | Matrix.af. and cost-effect*.ti. and randomized.ab | | 22† |
|  |  | Matrix.af. and cost-effect*.ti. and randomised.ab | | 5† |
|  |  | Matrix.af. and economic evaluation*.ab. and randomized.ab | | 9† |
|  |  | Matrix.af. and economic evaluation*.ab. and randomised.ab | | 5† |
|  |  | Matrix.af. and economic evaluation*.ti. and randomized.ab | | 4† |
|  |  | Matrix.af. and economic evaluation*.ti. and randomised.ab | | 4† |
| Bmj.com – advanced search | | cost-effective  (exact phrase in title or abstract)  factorial  (exact phrase anywhere in article) | | 5 |
|  |  | cost-effectiveness  (all words in title or abstract)  factorial  (all words anywhere in article) | | 5 |
|  |  | economic evaluation  (all words in title or abstract)  factorial  (all words anywhere in article) | | 1 |
|  |  | cost-effective (as phrase) in title or abstract and matrix randomised (all words) in full text. | | 6† |
|  |  | cost-effective (as phrase) in title or abstract and matrix randomized (all words) in full text. | | 4† |
|  |  | economic evaluation (as phrase) in title or abstract and matrix randomised (all words) in full text. | | 1† |
|  |  | economic evaluation (as phrase) in title or abstract and matrix randomised (all words) in full text. | | 1† |
|  |  | economic evaluation  (all words in title or abstract) | 2x2 phrase anywhere in article | 0 |
|  |  |  | 2 x 2 phrase anywhere in article | 0 |
|  |  |  | 2 by 2 phrase anywhere in article | 0 |
|  |  |  | Two by two phrase anywhere in article | 0 |
|  |  |  | Two x two phrase anywhere in article | 0 |
|  |  |  | 2 x 3 phrase anywhere in article | 0 |
|  |  |  | 3 x 3 phrase anywhere in article | 0 |
|  |  |  | 3 x 2 phrase anywhere in article | 0 |
|  |  |  | 2 x 2 x 2 phrase anywhere in article | 0 |
|  |  | Cost-effective  (all words in title or abstract) | 2x2 phrase anywhere in article | 0 |
|  |  |  | 2 x 2 phrase anywhere in article | 1 |
|  |  |  | 2 by 2 phrase anywhere in article | 0 |
|  |  |  | two x two phrase anywhere in article | 0 |
|  |  |  | two by two phrase anywhere in article | 3 |
|  |  |  | 2 x 3 phrase anywhere in article | 1 |
|  |  |  | 3 x 3 phrase anywhere in article | 0 |
|  |  |  | 3 x 2 | 1 |
|  |  |  | 2 x 2 x 2 | 0 |
|  |  | Cost-effectiveness  (all words in title or abstract) | 2x2 phrase anywhere in article | 0 |
|  |  |  | 2 x 2 phrase anywhere in article | 1 |
|  |  |  | 2 by 2 phrase anywhere in article | 0 |
|  |  |  | two x two phrase anywhere in article | 0 |
|  |  |  | two by two phrase anywhere in article | 2 |
|  |  |  | 2 x 3 phrase anywhere in article | 0 |
|  |  |  | 3 x 3 phrase anywhere in article | 0 |
|  |  |  | 3 x 2 phrase anywhere in article | 0 |
|  |  |  | 2 x 2 x 2 phrase anywhere in article | 0 |
| Science direct – journals and books, all years | | Expert search  TITLE-ABSTR-KEY("cost-effective" OR "cost-effectiveness" OR "economic evaluation") and (factorial OR "2x2" OR "2 x 2" OR "2 by 2" OR "two x two" OR "two by two" OR "2 x 3" OR "3 x 3" OR "3 x 2" OR "2 x 2 x 2") | | 457 |
|  |  | matrix cost-effective and TITLE-ABSTR-KEY(randomised) | | 5† |
|  |  | matrix cost-effective and TITLE-ABSTR-KEY(randomized) | | 93† |
|  |  | matrix "economic evaluation" and TITLE-ABSTR-KEY(**randomized**) | | 30† |
|  |  | matrix "economic evaluation" and TITLE-ABSTR-KEY(**randomised**) | | 7† |
| CRD (NHS EED, HTA and DARE | | factorial OR "2x2" OR "2 x 2" OR "2 by 2" OR "two x two" OR "two by two" OR "2 x 3" OR "3 x 3" OR "3 x 2" OR "2 x 2 x 2" | | 499 |
|  |  | matrix | | 82† |
| Tufts Advanced search searching articles. No limits | | Factorial | | 8 |
|  |  | 2x2 | | 0 |
|  |  | 2 x 2 | | 1 |
|  |  | 2 by 2 | | 0 |
|  |  | Two by two | | 0 |
|  |  | Two x two | | 0 |
|  |  | 2 x 3 | | 0 |
|  |  | 3 x 3 | | 0 |
|  |  | 3 x 2 | | 0 |
|  |  | 2 x 2 x 2 | | 0 |
|  |  | Matrix | | 2† |
|  |  | Interaction | | 3 |
| NIHR HTA publications list (<http://www.hta.ac.uk/>) | | Factorial | | 15 |
|  |  | 2x2 | | 7 |
|  |  | 2 x 2 | | ‡ |
|  |  | 2 by 2 | | ‡ |
|  |  | Two by two | | 4 |
|  |  | Two x two | | 4 |
|  |  | 2 x 3 | | ‡ |
|  |  | 3 x 3 | | ‡ |
|  |  | 3 x 2 | | ‡ |
|  |  | 2 x 2 x 2 | | ‡ |
|  |  | Interaction | | 60 |
|  |  | Matrix | | 21 |
| HESG papers (searching titles only). No quotations used | | factorial | | 0 |
|  |  | 2x2 | | 0 |
|  |  | 2 x 2 | | 0 |
|  |  | Two by two | | 0 |
|  |  | Two x two | | 0 |
|  |  | 2 x 3 | | 0 |
|  |  | 3 x 3 | | 0 |
|  |  | 3 x 2 | | 0 |
|  |  | 2 x 2 x 2 | | 0 |
|  |  | Matrix | | 0† |

† Searched on 3^rd^ September 2010

‡ The query contained only ignored words

Across all searches, a total of 1,671 references were identified (including duplicates). The titles and abstracts of these were scrutinised to identify relevant studies.

**References**

1. Dakin HA: Economic evaluation of factorial randomised controlled trials. University of Oxford; 2015.
