# Supplementary material for: Which interactions matter in economic evaluations? A systematic review and simulation study
Source: BMC Med Res Methodol. 2020 May 7;20:109. doi: 10.1186/s12874-020-00978-0 (PMC7203889; doi:10.1186/s12874-020-00978-0)
Supplement: Supplementary file 2 — Additional file 2. Data extraction table for the systematic review of studies conducting economic evaluations of factorial design studies. Includes full details on each study meeting inclusion criteria. [file 12874_2020_978_MOESM2_ESM.docx]

**Additional file 2: Data extraction table for the systematic review of studies conducting economic evaluations of factorial design studies**

**Table 2.1.** Completed studies

| **Author/study acronym** | | **ACUITY [1-4]** | **ADAPT [5-7]** | **AFIST II [8, 9]** |
| --- | --- | --- | --- | --- |
| Year of publication for earliest paper presenting ***methods or results*** of economic evaluation | | 2008 | 2003 | 2004 |
| Year of publication for earliest paper presenting ***methods*** of economic evaluation | | 2008 | 2003 | 2004 |
| Year of publication for earliest paper presenting ***results*** of economic evaluation | | 2008 | 2009 | 2004 |
| Type of factorial design (e.g. partial/fractional) | | Full factorial | Full factorial | Full factorial |
| Total sample size (across all arms) | | 13819 | 316 | 160 |
| Size of factorial matrix (e.g. 2x2) | | 2x2 + 1 | 2x2 | 2x2 |
| Disease area | | Acute coronary syndromes | Knee osteoarthritis | Open-heart surgery for coronary artery disease |
| Sponsor | | Manufacturer | US public funding body | Manufacturer and other funding body |
| Country | | US, Europe, Australia, NZ and Canada. Economic evaluation focusses on US patients | US | US |
| Treatments (or treatment combinations) compared | | Factor 1: bivalirudin vs. unfractionated heparin or enoxaparin. Factor 2: "upstream" routine glycoprotein (GP) Iib/IIIa inhibitor (given to all pts immediately) vs. deferred selective GP Iib/IIIa (given immediately before PCI in those pts who underwent PCI). Arm 5: bivalirudin with no GP IIb/IIIa. | Factor 1: Exercise programme 3-times a week vs. no exercise programme. Factor 2: 18 sessions of dietary counselling vs. no dietary counselling. Patients in the control group had a healthy lifestyle group session monthly for first 3 months, with phone contact thereafter | Factor 1: Amiodarone vs. placebo. Factor 2: pacing vs. no pacing. |
| Type of comparisons | | A1 vs. A2 vs. B1 vs. B2 (≥2 dosing regimens of ≥2 interventions) | *0* vs. *a* vs. *b* vs. *ab* | 0 vs. a vs. b vs. ab |
| Type(s) of treatment evaluated | | Pharmaceutical | Nutritional supplement/diet + exercise | Pharmaceutical and surgery |
| Power calculation allowed for interaction effect and/or powered to test for interaction? | | Appears to have been powered for at-the-margins comparisons between bivalirudin alone vs. bivalirudin+GPIIb/IIIa vs. heparin+GPIIb/IIIa. Power calculation adjusts for multiple comparisons as 2 primary analyses involve comparison with common treatment arm. | Powered for main effects. "The main effects of weight reduction and physical activity interventions were felt to be additive or nearly additive. If the effect of one factor is additive by a factor p then the joint effect of weight reduction and lifestyle physical activity intervention would have a joint effect of 10% × (1 p). Sample size calculations were made to allow for 75–100% additivity." | Not stated. Interactions not mentioned |
| Type of economic evaluation (e.g. CUA) | | CCA | CEA | CEA |
| Other notable aspects of study design | Cluster randomised | No | No | No |
|  | Cross-over | No | No | No |
|  | Intentionally unbalanced | No | No | No |
|  | Interventions target different diseases | No | No | No |
|  | Other | Trial powered for sequential tests of non-inferiority and superiority |  |  |
| Form of statistical analysis to deal with factorial design (e.g. inside-the-table): including type of regression model if appropriate | For primary clinical outcome | At-the-margins | Linear regression/ANCOVA/ANOVA | Linear regression/ANCOVA/ANOVA |
|  |  |  | Not stated | No mention of interaction term |
|  |  | Main analysis treated the study as a 3-arm trial and compared bivalirudin+GPIIb/IIIa vs. heparin+GPIIb/IIIa and then compared bivalirudin alone vs. heparin+GPIIb/IIIa. Present subgroup analyses by timing of GP IIb/IIIa; comparions between upstream and deferred GP IIb/IIIa are secondary endpoint. | Unclear how ANCOVA was conducted or whether it controlled for both treatments. Results presented for each of the 4 groups; text reports results of comparison between each of the 3 active groups vs. healthy lifestyle sessions | "Power analysis was for a 2x2 fixed effects analysis of variance"; also describe methods for pair-wise comparisons |
|  | Base case economic evaluation | Inside-the-table (treating each cell separately) | Inside-the-table (treating each cell separately) | Inside-the-table (treating each cell separately) |
|  | Sensitivity analyses of economic evaluation | None | None | At-the-margins |
|  |  |  |  | Costs and cost-effectiveness presented on an at-the-margins basis |
| Presentation of economic results | | Present only costs not ICERs. | Each active group is compared with healthy lifestyle | Costs are compared on an at-the-margins basis, but cost-effectiveness results are presented on inside-the-table basis and at-the-margins basis |
| Presentation of uncertainty (e.g. pair-wise CEAC, EVPI) | | Conducted bootstrapping and present CEAC-like graphs plotting the cummulative proportion of bootstrap replicates against estimated incremental cost; these are presented for bivalirudin monotherapy vs. heparin+upstream GPIIb/IIIa and for bivalirudin monotherapy vs. heparin+deferred GPIIb/IIIa | Present SD around healthcare costs, but otherwise appear to give no indication of the precision around incremental costs or cost-effectiveness. | Cost-effectiveness results are presented on inside-the-table basis and at-the-margins basis as the proportion of bootstrap replicates lying in each quadrant relative to placebo group |
| Statistical significance of differences of each comparison individually | | No significant difference between bivalirudin alone, bivalirudin+GPIIb/IIIa and heparin +GPIIb/IIIa. | Exercise+diet significantly superior to healthy lifestyle. No significant differences between exercise only or diet only groups and the healthy lifestyle group | Clinical paper concludes that A is significantly superior to non-A but that pacing had no significant benefits. However, abstract of economic paper says that A+pacing is significantly superior to all three of the other treatment arms. |
| Significance of interaction effect | | Unclear. Present interactions or subgroup analyses for heparin+GPIIb/IIIa vs. "bivalirudin alone" that include subgrouping by upstream vs. deferred GPIIb/IIIa; unclear whether this is a typo or how interactions were assessed for this comparison, although Figure 3 of Stone 2007 suggests that there is a non-significant qualitative interaction between upstream/downstream and heparin+GPIIb/IIIa vs. bivalirudin alone for composite ischaemia in pts having PCI. Pinto et al note that there was no significant interaction for 30-day cost savings with bivalirudin monotherapy compared with heparin + upstream/deferred GPIIb/IIIa | Not stated | Interactions not mentioned, although discussion of an "apparent synergistic effect" was noted in the discussion of the economic paper. The differing conclusions of clinical and economic papers and the probability of pacing being in the NW or SW quadrants also imply that there may be a large *qualitative* interaction for efficacy and cost |
| Results of comparing different methods for statistical analysis | | N/A | N/A | Probability that treatment is cost-effective was substantially higher in at-the-margins analysis than inside-the-table analysis. Notably, the probability that pacing increased efficacy compared with placebo was 79% based on at-the-margins analysis and 41% based on inside-the-table analysis, suggesting a qualitative interaction |

| **Author/study acronym** | | **ASCOT 1 [10-17]** | **ATEAM [18, 19]** | **Bankhead et al 2001 [20, 21]** |
| --- | --- | --- | --- | --- |
| Year of publication for earliest paper presenting ***methods or results*** of economic evaluation | | 2005 | 2008 | 2006 |
| Year of publication for earliest paper presenting ***methods*** of economic evaluation | | 2005 | 2008 | 2006 |
| Year of publication for earliest paper presenting ***results*** of economic evaluation | | 2005 | 2008 | 2006 |
| Type of factorial design (e.g. partial/fractional) | | Partial factorial | Full factorial | Full factorial |
| Total sample size (across all arms) | | 19,257 | 579 | 1158 |
| Size of factorial matrix (e.g. 2x2) | | 2x2 | 2x4 | 2x2 |
| Disease area | | Cardiovascular disease | Back pain | Breast cancer screening |
| Sponsor | | Manufacturer | MRC | MRC |
| Country | | UK, Ireland and Scandinavia | UK | UK |
| Treatments (or treatment combinations) compared | | Factor 1 (all patients): amlodipine adding perindopril as required vs. atenolol adding bendroflumethiazide. Factor 1 (pts with low cholesterol): atorvastatin vs. placebo | Factor 1: Normal care vs. massage vs. 6 lessons in Alexander technique vs. 24 lessons in the Alexander technique. Factor 2: exercise prescription from a doctor plus behavioural counselling from a nurse vs. no exercise prescription and counselling. | Factor 1: letter from GP reminding about screening vs. no letter. Factor 2: flag in women's notes prompting discussion of screening, vs. no flag. |
| Type of comparisons | | A0 vs. AX vs. B0 vs. BX (Factor 1: interventions A and B; Factor 2: X vs. 0) | 0 vs. a vs. b vs. ab | 0 vs. a vs. b vs. ab |
| Type(s) of treatment evaluated | | Pharmaceutical | Physiotherapy and related techniques | Logistical intervention targetted at health service |
| Power calculation allowed for interaction effect and/or powered to test for interaction? | | No | "Powered for only moderately large interactions" | Not stated |
| Type of economic evaluation (e.g. CUA) | | CEA and CUA | CEA and CUA | CEA |
| Other notable aspects of study design | Cluster randomised | No | No | No |
|  | Cross-over | No | No | No |
|  | Intentionally unbalanced | No | No | No |
|  | Interventions target different diseases | No | No | No |
|  | Other | Lipid comparison was prematurely terminated two years before the blood pressure comparison. Results of each comparison published entirely separately |  |  |
| Form of statistical analysis to deal with factorial design (e.g. inside-the-table): including type of regression model if appropriate | For primary clinical outcome | At-the-margins | Linear regression/ANCOVA/ANOVA | Logistic regression |
|  |  | No mention of interaction term | Interaction term included regardless of significance | No interaction term included |
|  |  | Analysed using survival data. Each comparison was reported entirely separately |  | Adjusted for practice with FE model. "The adjusted odds ratios (ORs) for attendance and their associated 95% CIs are presented, and the interaction between the two interventions was also investigated. Also, crude relative risks (RRs) and 95% CIs for the effects of the two interventions were calculated, with the interaction effect for RRs (95% CIs) estimated from simple stratified analyses." Table 4 states that the OR for main effects are based on a model that did not contain an interaction term. |
|  | Base case economic evaluation | Event-based CEA | Inside-the-table (treating each cell separately) | Logistic regression |
|  |  | Interaction term included regardless of significance |  | Interaction term included regardless of significance |
|  |  | Model-based economic evaluation using IPD, in which risk of event was based on survival analysis model including dummies for 3 of the 4 treatment groups (amlodipine-based therapy plus atorvastatin, atenolol-based therapy plus atorvastatin or amlodipine-based therapy plus placebo, with atenolol plus placebo as the reference category) [12]. | Present outcomes for the 8 arms separately and calculate ICERs vs. specific comparisons. These comprised the "main" results. | Logistic regression model in WinBUGS, adjusting for clustering by practice and an interaction term. However, interaction term was assigned an informative prior that it is unlikely (with 95% probability) to exceed the magnitude of the main effects |
|  | Sensitivity analyses of economic evaluation | At-the-margins | At-the-margins | Linear regression/ANCOVA/ANOVA |
|  |  | 2008 paper used within-trial and event-based model approach to compare atenolol and amlodipine [11], while 2005 paper used within trial approach to compare atorvastatin vs. no statin [10], although neither considered factorial nature of trial. |  | Economic evaluation in primary clinical paper presented only point estimate for ICER (mean incremental cost/mean incremental effect) for flag or letter vs. control. |
| Presentation of economic results | | 2009 paper presented regression coefficients and costs separately for each of the 4 arms and calculated ICERs along the frontier [12]. 2005 and 2008 papers presented results separately for one pair-wise comparison [10, 11]. | Present outcomes for each of the 8 groups separately "to provide the most relevant information for policy makers" and conducted at-the-margins analysis "in line with the convention for a factorial design [referencing Fox et al 2006]". The authors compared each intervention group with "the most appropriate comparator group" [which generally comprised the group that would be expected to be the next most effective] | Brown et al presented incremental cost and additional numbers of attendences for each active treatment arm vs. control group and presented incremental outcomes along the frontier, although the original paper gave only CER vs. control. |
| Presentation of uncertainty (e.g. pair-wise CEAC, EVPI) | | 2009 paper conducted PSA and presented CEACs for multiple comparisons [12]. 2005 and 2008 papers presented CEACs for single comparisons from PSA and/or bootstrapping [10, 11]. | Present pariwise CEACs for a number of pair-wise comparisons between individual arms (based on bootstrapping) | Presented incremental costs & effects vs. control on CE and CEAC frontier and EVPI allowing for multiple comparisons. These were calculated in WinBUGS. |
| Statistical significance of differences of each comparison individually | | Atenolol was non-significantly superior to amlodipine for primary endpoint and significantly superior for mortality. Atorvastatin significantly superior to placebo for primary endpoint but not mortality. | Exercise and lessons in the Alexander technique, but not massage, remained effective at one year compared with control. | Letter had a significant effect. Flag had no significant effect |
| Significance of interaction effect | | Significant interaction was observed for CHD events [16]. Interactions not mentioned in economic papers. Interactions for costs and QALYs were not reported in papers, although I estimated there to be a small quantitative sub-additive interaction for costs and tiny super-additive interaction for QALYs. | Not significant, although authors commented that "the effect of exercise and 24 Alexander technique lessons combined is less than the sum of the two individual effects" | Not significant. "the interaction OR of less than 1 suggests that there would be little to gain from combining the two interventions." Not clear what they mean by interaction OR [presumably exponent of the coefficient for interaction term |
| Results of comparing different methods for statistical analysis | | Comparing CUA results for UK from 2008 and 2009 papers, the two papers produce analogous conclusions (that amlodipine is cost-effective vs. atenolol [11] or that amlodipine+atorvastin maximises NB [12]), although estimated costs & effects for all arms are slightly different due to different methodology, making direct comparisons difficult. | ICERs differed substantially between at-the-margins and inside-the-table analyses - especially for massage, which was dominated by control based on inside-the-table analysis of the non-exercise groups, but cost £10,800/QALY based on at-the-margins approach. | ICER for letter vs. control was £41/attendance in Welton analysis that allowed for uncertainty and included interaction term, vs. £35/attendance in original clinical paper. By taking an incremental analysis, Brown et al concluded that the flag was inefficient (being extendedly dominated by the other 2 interventions), whereas the primary clinical paper concluded only that flag was less cost-effective than the letter based on CERs for each intervention vs. control |

| **Author/study acronym** | | **BARI 2D [22-24]** | **Barnett/Sorensen [25, 26]** | **BELLS [27-29]** |
| --- | --- | --- | --- | --- |
| Year of publication for earliest paper presenting ***methods or results*** of economic evaluation | | 2009 | 2006 | 2009 |
| Year of publication for earliest paper presenting ***methods*** of economic evaluation | | 2009 | 2006 | 2009 |
| Year of publication for earliest paper presenting ***results*** of economic evaluation | | 2009 | 2006 | 2009 |
| Type of factorial design (e.g. partial/fractional) | | Full factorial | Full factorial | Full factorial |
| Total sample size (across all arms) | | 2368 | 126 | 496 |
| Size of factorial matrix (e.g. 2x2) | | 2x2 | 2x2 | 2x2 |
| Disease area | | Patients with both type 2 diabetes and coronary artery disease | Drug abuse | Bell's Palsy |
| Sponsor | | Manufacturer and other funding body | US public funding body | NIHR HTA |
| Country | | Multinational | US | UK (Scotland) |
| Treatments (or treatment combinations) compared | | Factor 1: prompt coronary revascularisation or medical therapy. Factor 2: insulin sensitisation therapy or insulin provision therapy | Factor 1: voucher for free methadone treatment vs. no voucher. Factor 2: 6 months' case management services vs. no such services. | Factor 1: prednisolone vs. placebo. Factor 2: aciclovir vs. placebo. |
| Type of comparisons | | 0 vs. a vs. b vs. ab | 0 vs. a vs. b vs. ab | 0 vs. a vs. b vs. ab |
| Type(s) of treatment evaluated | | Pharmaceutical and surgery | Other | Pharmaceutical |
| Power calculation allowed for interaction effect and/or powered to test for interaction? | | Not stated. | Not stated | Assumed no interaction between factors in power calculation, alhtough power to detect main effects in the presence of a negative interaction was also estimated |
| Type of economic evaluation (e.g. CUA) | | CEA and CUA | CCA | CEA and CUA |
| Other notable aspects of study design | Cluster randomised | No | No | No |
|  | Cross-over | No | No | No |
|  | Intentionally unbalanced | No | No | No |
|  | Interventions target different diseases | No | No | No |
|  | Other | Population was divided into 2 subgroups basedon whether the responsible physician felt that CABG or PCI was more apporpriate |  |  |
| Form of statistical analysis to deal with factorial design (e.g. inside-the-table): including type of regression model if appropriate | For primary clinical outcome | At-the-margins | At-the-margins | At-the-margins |
|  |  | No interaction term included |  | Interaction term excluded as it was not statistically significant |
|  |  | Protocol states that the two randomised comparisons will be evaluated separately, with no adjustment for multiple comparisons. Methods not clear in main papers presenting results: presentation of results implies at-the-margins analysis, although also comments that: "In addition, the statistical interactions between the cardiac study groups and the glycemic study groups for rates of death and major cardiovascular events were tested overall and within the PCI and CABG strata at a two-sided alpha level of 0.05". Also mention results of what appears to be a inside-the-table analysis. | Defined 4 primary hypotheses, of which (a), (b) and (c ) appear to have been based on at-the-margins analysis, and (d) was based on inside-the-table comparison between cell 00 and cell AB. | Tested data for any interaction prior to evaluating primary endpoint |
|  | Base case economic evaluation | At-the-margins | Inside-the-table (treating each cell separately) | At-the-margins |
|  |  |  |  | Interaction term excluded as it was not statistically significant |
|  |  | Assumed to be at-the-margins analysis based on the way in which results are presented, although this is not explicitly stated | Costs and outcomes are first compared between individual cells of the design. Statistical tests presented comparing costs & effects between pairs of arms | Estimated using two decision-tree models (one for each factor) based largely on trial data that compared costs and outcomes with and without that factor |
|  | Sensitivity analyses of economic evaluation | None | At-the-margins | Inside-the-table (treating each cell separately) |
|  |  |  | Costs also compared on at-the-margins basis+BC55 | Estimated using a decision-tree model with 4 treatment strategies (1 for each group in the trial) |
| Presentation of economic results | | Presented costs and benefits for medical therapy vs. revascularisation (stratified by whether PCI or CABG were most suitable) and for insulin sensitisation vs. insulin provision. | No ICERs presented; both costs and effects are presented but not combined. No clear indication of whether inside-the-table or at-the-margins is base case, although abstract focuses on former | For base case, presented outcomes of at-the-margins analysis |
| Presentation of uncertainty (e.g. pair-wise CEAC, EVPI) | | Presented pair-wise probability cost-effective at 2 thresholds, but no CEACs | No ICERs presented | Presented CEACs for pair-wise comparisons for the base case at-the-margins analyses. For sensitivity analysis comparing 4 arms individually, also presented CEACs for multiple comparisons and probability that each treatment is optimal. |
| Statistical significance of differences of each comparison individually | | No significant differences between revascularisation and medical therapy or between insulin sensitisation and insulin provision | Both case management and vouchers significantly increase costs in at-the-margins analysis. Vouchers significantly increased enrollment on methadone, although other differences were not significantly different. | Prednisolone found to significantly increase chance of recovery, although aciclovir had no significant effect. |
| Significance of interaction effect | | Interaction not significant in overall group, although interaction was borderline significance (p=0.07 for those patients who were more suitable for CABG than PCI. Methods to test for interaction unclear. | Interactions not mentioned in Barnett et al and results of clinical analyses including interaction term were not stated | No significant difference |
| Results of comparing different methods for statistical analysis | | N/A | At-the-margins and inside-the-table give same conclusions. | Inside-the-table analysis (which showed prednisolone without aciclovir to strongly dominate other strategies) largely confirmed findings of at-the-margins analysis (which showed prednisolone to dominate no prednisolone and no aciclovir to dominate aciclovir). |

| **Author/study acronym** | | **Boyle 2007 [30, 31]** | **Brandon et al 2000 [32]** | **Brandon et al 2004 [33]** |
| --- | --- | --- | --- | --- |
| Year of publication for earliest paper presenting ***methods or results*** of economic evaluation | | 2007 | 2000 | 2004 |
| Year of publication for earliest paper presenting ***methods*** of economic evaluation | | 2007 | 2000 | 2004 |
| Year of publication for earliest paper presenting ***results*** of economic evaluation | | 2007 | 2000 | 2004 |
| Type of factorial design (e.g. partial/fractional) | | Full factorial | full factorial | full factorial |
| Total sample size (across all arms) | | 161 | 446 | 431 |
| Size of factorial matrix (e.g. 2x2) | | 2x2 + 1 | 2x2 | 2x2 |
| Disease area | | Language impairment in children | Smoking cessation | Smoking cessation |
| Sponsor | | NIHR HTA | US public funding body | US public funding body |
| Country | | UK | US | US |
| Treatments (or treatment combinations) compared | | Factor 1: Direct vs. indirect therapy. Factor 2: individual vs. group therapy. There was also a control group receiving usual care | Factor 1: telephone hotline vs. no telephone hotline. Factor 2: repeated relapse prevention mailings vs. no mailings | Factor 1: 1 mailing of material to prevent relapse of smoking vs. 8 mailings. Factor 2: 1 booklet vs. 8 booklets |
| Type of comparisons | | Other | 0 vs. a vs. b vs. ab | Dose-ranging for 1 drug (e.g. freq x dose) |
| Type(s) of treatment evaluated | | Other | Other | Other |
| Power calculation allowed for interaction effect and/or powered to test for interaction? | | Yes (if interaction effect was 0.50, compared with power to detect a 0.40 difference for main effects) | No power calculation | No power calculation |
| Type of economic evaluation (e.g. CUA) | | CEA | CEA | CEA and CUA |
| Other notable aspects of study design | Cluster randomised | No | no | No |
|  | Cross-over | No | no | No |
|  | Intentionally unbalanced | No | no | No |
|  | Interventions target different diseases | No | no | No |
|  | Other |  |  |  |
| Form of statistical analysis to deal with factorial design (e.g. inside-the-table): including type of regression model if appropriate | For primary clinical outcome | Linear regression/ANCOVA/ANOVA | Linear regression/ANCOVA/ANOVA | Logistic regression |
|  |  | Interaction term excluded as it was not statistically significant | Interaction term excluded as it was not statistically significant | Interaction term included regardless of significance |
|  |  | Controlled for covariates | Analysed various outcomes using ANOVA. Found hotline to have no significant effect and no interaction with mailings, so focus on mailings comparison. No explicit statement about whether or not the interaction term was or was not included in the final model, but I am assuming that it was omitted on grounds that it was non-significant. | "Interaction term, Contact x Content, which was entered as a second step in the logistic regression equation" |
|  | Base case economic evaluation | Inside-the-table (treating each cell separately) | At-the-margins | Inside-the-table (treating each cell separately) |
|  |  |  | No mention of interaction term |  |
|  |  | Compared total costs using ANOVA. Pair-wise comparisons conducted using t-tests. Means and 95% CIs for each group appear to have been calculated using bootstrapping and analytical methods. Main method of presentation appears to be inside-the-table analysis | Simple comparison of cost/incremental benefits for mailings vs. no mailings. | Each group is compared with control group |
|  | Sensitivity analyses of economic evaluation | At-the-margins | None | None |
|  |  | Costs, outcomes and cost-effectiveness also compared on an at-the-margins basis |  |  |
| Presentation of economic results | | Gave outcomes separately for each arm and at-the-margin. Compared costs for all direct therapy groups vs. control and calculates **average** cost-effectiveness ratios for each of the 5 groups. In Dickinson, 2009, the authors focus on costing results and present "cost ratios" for pair-wise comparisons equal to the cost in one group divided by the cost in a comparator group; some of these ratios appear to compare cells inside-the-table and others appear to be at-the-margins. | Cost and ICER mentioned in text only | Present incremental costs, QALYs, abstinence rates and ICERs compared with control group |
| Presentation of uncertainty (e.g. pair-wise CEAC, EVPI) | | Two-way sensitivity analysis | None | None |
| Statistical significance of differences of each comparison individually | | No significant differences in primary endpoint; direct therapy superior in some secondary endpoints. Significant differences in cost | Hotline not significant. Mailing significantly superior to no mailings on several endpoints. | Giving 8 booklets rather than 1 significantly reduced relapse rates, although frequency of booklet mailing didn’t affect outcome |
| Significance of interaction effect | | Not significant | Not significant | Not significant, although Table 3 implies that there is a qualitative interaction, with repeated letters producing lower abstinence rates than control group, but with repeated mailings increasing abstinence rates compared with sending all 8 booklets at once. |
| Results of comparing different methods for statistical analysis | | Inside-the-table analysis suggests that therapist-led group therapy dominates all treatments except control. At-the-margins suggests that group dominates individual therapy, but that therapist-led therapy is more costly and more effective than assistant-led therapy | N/A | N/A |

| **Author/study acronym** | | **CADILLAC [34, 35]** | **Cantor/Morisky/Green [36-38]** | **CELL [39, 40]** |
| --- | --- | --- | --- | --- |
| Year of publication for earliest paper presenting ***methods or results*** of economic evaluation | | 2003 | 1985 | 1996 |
| Year of publication for earliest paper presenting ***methods*** of economic evaluation | | 2003 | 1985 | 1996 |
| Year of publication for earliest paper presenting ***results*** of economic evaluation | | 2003 | 1985 | 1996 |
| Type of factorial design (e.g. partial/fractional) | | Full factorial | Full factorial | Full factorial |
| Total sample size (across all arms) | | 2082 | 400 | 681 |
| Size of factorial matrix (e.g. 2x2) | | 2x2 | 2x2x2 | 2x3 |
| Disease area | | Acute myocardial infarction | Hypertension | Primary prevention of cardiovascular events |
| Sponsor | | Manufacturer | US public funding body | Manufacturer and other funding body |
| Country | | US (Multinational trial, although economic evaluation focusses on US patients) | US | Sweden |
| Treatments (or treatment combinations) compared | | Factor 1: PTCA vs. stenting with MultiLink stent. Factor 2: abciximab vs. no abciximab | Factor 1: Exit interview encouraging compliance immediately after physician appointment (E1) vs. no exit interview (C1). Factor 2: Interview with spouse to encourage compliance (E2) vs. no interview (C2). Factor 3: series of up to 3 group sessions with a social worker (E3) vs. no sessions (C3) | Factor 1: Intensive advice (group sessions) vs. usual advice. Factor 2: pravastatin vs. usual care vs. no drug. |
| Type of comparisons | | 0 vs. a vs. b vs. ab | 0 vs. a vs. b vs. ab | Other |
| Type(s) of treatment evaluated | | Pharmaceutical and surgery | Other | Pharmaceutical + other |
| Power calculation allowed for interaction effect and/or powered to test for interaction? | | Not stated. Appears to have been powered for a inside-the-table analysis | No power calculation? (no apparent mention of rationale for sample size in Morisky 1983 or Green 1975) | Appears to be powered for inside-the-table analysis, although not entirely clear |
| Type of economic evaluation (e.g. CUA) | | CUA | CEA | CEA |
| Other notable aspects of study design | Cluster randomised | No | No | No |
|  | Cross-over | No | No | No |
|  | Intentionally unbalanced | No | No | No |
|  | Interventions target different diseases | No | No | No |
|  | Other | Designed for non-inferiority wrt the comparison between stent alone and PTCA+abciximab | Interventions given at different times: factor 1 first, then factor 2 a few weeks later and then factor 3 |  |
| Form of statistical analysis to deal with factorial design (e.g. inside-the-table): including type of regression model if appropriate | For primary clinical outcome | Inside-the-table (treating each cell separately) | Inside-the-table (treating each cell separately) | Inside-the-table (treating each cell separately) |
|  |  | "Two primary hypotheses were prespecified: stenting without abciximab is superior to PTCA without abciximab, and stenting without abciximab is not inferior to PTCA plus abciximab." The majority of the results are presented separately for each of the 4 arms, with p-values for the difference between any of the groups being calculated from one-way ANOVA. However, the OR for all pts receiving stent vs. all patients receiving PTCA was shown in a figure with subgroup analyses. | Appear to have compared each group with the control group |  |
|  | Base case economic evaluation | At-the-margins | Inside-the-table (treating each cell separately) | Inside-the-table (treating each cell separately) |
|  |  |  | Compare each arm with control | Excludes intensive advice+drug arm as it is dominated (no more effective and more costly) and compares costs & calculates ICERs for drug only, intensive advice only and no treatment (although it is unclear whether the no treatment arm is based on the usual advice only arm or assuming no cost and no change in risk from baseline). Based on patient numbers, the economic evaluation appears to have excluded patients randomised to placebo and include only men on active drug or no drug. |
|  | Sensitivity analyses of economic evaluation | None | None | None |
| Presentation of economic results | | Presented costs and effects in at-the-margins format | Present CERs for each arm vs. control based on a wide range of outcomes. Don't present ICERs along the frontier, but do present results on CE plane and identify strongly dominated but not extendedly dominated options and present marginal costs and effects excluding strongly dominated options. | Present costs for all 4 arms, plus incremental costs, life-years gained and ICERs for intensive advice vs. no treatment and usual advice+drug vs. no treatment and drug+usual advice vs. intensive advice |
| Presentation of uncertainty (e.g. pair-wise CEAC, EVPI) | | Estimated probability cost-effective for stenting vs. PTCA, but did not present CEACs | None | Scenario analysis only |
| Statistical significance of differences of each comparison individually | | Significant differences between groups when all 4 groups compared [in 1-way ANOVA]. Stenting alone significantly superior to PTCA alone but non-inferior to PTCA+abciximab. In economic evaluation, abciximab was found to non-significantly reduce number of QALYs accrued when mortality difference were ignored, and non-significantly increase QALYs when mortality differences were included. | Authors conclude that the programmes significantly improved appointment keeping, weight control, blood pressure control and mortality. | Pravastatin significantly reduces Framingham risk score. No difference between placebo and no drug. Advice has small but statistically significant effect in pts not having active drug, but no effect in patients on placebo or no drug |
| Significance of interaction effect | | Not stated | not stated | Significance of interaction not stated, though a qualitative interaction clearly exists at 12 and 18 months based on Figure 1. Advice has small but statistically significant effect in pts not having active drug, but no effect in patients on placebo or no drug |
| Results of comparing different methods for statistical analysis | | N/A | N/A | N/A |

| **Author/study acronym** | | **COPE II [41]** | **ERUPT [42]** | **Exercise Intervention Trial [43]** |
| --- | --- | --- | --- | --- |
| Year of publication for earliest paper presenting ***methods or results*** of economic evaluation | | 2009 | 2008 | 2002 |
| Year of publication for earliest paper presenting ***methods*** of economic evaluation | | 2009 | 2008 | 2002 |
| Year of publication for earliest paper presenting ***results*** of economic evaluation | | 2009 | 2008 | 2002 |
| Type of factorial design (e.g. partial/fractional) | | Full factorial | Full factorial | full factorial |
| Total sample size (across all arms) | | 159 | 2833 | 4999 |
| Size of factorial matrix (e.g. 2x2) | | 2x2 | 2x2 | 2x2 |
| Disease area | | asthma | Dentistry | Breast cancer prevention |
| Sponsor | | Other public funding body | Other UK public funding body | US public funding body |
| Country | | Netherlands | UK (Scotland) | US |
| Treatments (or treatment combinations) compared | | Factor 1: self-treatment of exacerbations (and self-management programme) vs. self-management programme only; Factor 2: COPE-active physiotherapy exercise programme vs. no COPE-active | Factor 1: fee (financial incentive for applying sealant) vs. no fee. Factor 2: 1-day education workshop on evidence-based practice vs. no workshop. | Factor 1: inclusion/exclusion of personal invitation letter. Factor 2: first class stamps vs. bulk mail |
| Type of comparisons | | 0 vs. a vs. b vs. ab | 0 vs. a vs. b vs. ab | A0 vs. AX vs. B0 vs. BX (Factor 1: interventions A and B; Factor 2: X vs. 0) |
| Type(s) of treatment evaluated | | Physiotherapy and related techniques | Logistical intervention targetted at health service | Other |
| Power calculation allowed for interaction effect and/or powered to test for interaction? | | Appears to have been powered based on no interaction. Not explicitly stated in sample size section, although it is noted at the beginning of the methods that "this design assumes that both interventions do not interact with each other" | Not powered to detect clinically-significant interaction, and appears to have been powered based on at-the-margins analysis "(fee vs. no fee; education vs. no education)" | No power calculation described (primary aim was to randomise women to RCT) |
| Type of economic evaluation (e.g. CUA) | | CEA | CEA | CEA |
| Other notable aspects of study design | Cluster randomised | No | Yes | no |
|  | Cross-over | No | No | no |
|  | Intentionally unbalanced | No | No | no |
|  | Interventions target different diseases | Not stated | No | no |
|  | Other | Factors administered sequentially: physiotherapy given in months 2-12, whereas self-management and self treatment training given in month 1 |  |  |
| Form of statistical analysis to deal with factorial design (e.g. inside-the-table): including type of regression model if appropriate | For primary clinical outcome | At-the-margins | Linear regression/ANCOVA/ANOVA | Logistic regression |
|  |  | Interaction term excluded as it was not statistically significant | No mention of interaction term | Interaction term excluded as it was not statistically significant |
|  |  | Chi-square tests used to compared proportion of patients with an individual value for the exacerbation days/year larger than the median, the 75th and the 90th percentile. However, results are only presented for self treatment vs. control (not for COPE physiotherapy) | Means presented as inside-the-table and at-the-margins, although differences generally only presented for at-the-margins analysis. |  |
|  | Base case economic evaluation | At-the-margins | Inside-the-table (treating each cell separately) | Unclear |
|  |  |  |  | Not stated |
|  |  | Cost-effectiveness evaluated using a decision-analytical model employing patient-level data on resource use. Economic evaluation only presented on self treatment vs. control (not on COPE physiotherapy) | Simply presented fees and outcomes for each group | Appears to be at-the-margins. Simply present ACERs of cost/pt randomised for first-class mail and for bulk rate mail |
|  | Sensitivity analyses of economic evaluation |  | None | None |
|  |  |  | N/A |  |
| Presentation of economic results | |  | Presented ICER for each active treatment group vs. control. Costs not reported. | Simply present ACERs of cost/pt randomised for first-class mail and for bulk rate mail |
| Presentation of uncertainty (e.g. pair-wise CEAC, EVPI) | | Cost-effectiveness planes for pair-wise comparison between self-treatment and no self-treatment | None | None |
| Statistical significance of differences of each comparison individually | | Self-treatment vs. control significant and some outcomes but not others. No results presented for COPE this year therapy. Study doesn't explicitly state which is the primary endpoint, although number of exacerbation days appears to be main endpoint, while being statistically tested in three different ways. | Fee significantly increased sealant use, while education had no significant effect. | First class mail non-significantly increased responses vs. bulk mail. Including personal letter had no effect |
| Significance of interaction effect | | Non-significant? ("Before the final analysis we checked for interactions between both interventions and concluded that no interaction was present") | **Non-significant qualitative interaction for primary endpoint.** | Non-significant |
| Results of comparing different methods for statistical analysis | | N/A | N/A | N/A |

| **Author/study acronym** | | **FRISC II [44-49]** | **GISSI-3 [50, 51]** | **GISSI-P [52, 53]** |
| --- | --- | --- | --- | --- |
| Year of publication for earliest paper presenting ***methods or results*** of economic evaluation | | 2003 | 1998 | 2001 |
| Year of publication for earliest paper presenting ***methods*** of economic evaluation | | 2003 | 1998 | 2001 |
| Year of publication for earliest paper presenting ***results*** of economic evaluation | | 2003 | 1998 | 2001 |
| Type of factorial design (e.g. partial/fractional) | | Partial factorial | full factorial | full factorial |
| Total sample size (across all arms) | | 3469 | 19394 | 11324 |
| Size of factorial matrix (e.g. 2x2) | | 2x2 | 2x2 | 2x2 |
| Disease area | | Myocardial infarction | Acute myocardial infarction | 2ndary prevention of myocardial infarction |
| Sponsor | | Manufacturer and other funding body | manufacturer | Manufacturer |
| Country | | Scandinavia | Italy | Italy |
| Treatments (or treatment combinations) compared | | Factor 1: Dalteparin vs. placebo. Factor 2: Invasive therapy vs. non-invasive. Patients were randomised to both comparisons unless they were contraindicated to invasive therapy or were recruited after the invasive therapy comparison had stopped recruiting | Factor 1: Lisinopril vs. open-label control. Factor 2: nitrates (IV nitrates for first 24 hours, then transdermal GTN) vs. open-label control. | Factor 1: n-3 polyunsaturated fatty acids (PUFA) vs. placebo. Factor 2: vitamin E vs. placebo. |
| Type of comparisons | | A0 vs. AX vs. B0 vs. BX (Factor 1: interventions A and B; Factor 2: X vs. 0) | 0 vs. a vs. b vs. ab | 0 vs. a vs. b vs. ab |
| Type(s) of treatment evaluated | | Pharmaceutical and surgery | Pharmaceutical | Nutritional supplement |
| Power calculation allowed for interaction effect and/or powered to test for interaction? | | Not clearly stated, although power calculations appear to have been done separately for each comparison, suggesting that was powered for at-the-margins. Authors state that main reason for using factorial design was efficiency not to estimate interactions. | Not stated | Powered for inside-the-table analysis |
| Type of economic evaluation (e.g. CUA) | | CEA | CEA | CEA |
| Other notable aspects of study design | Cluster randomised | No | No | No |
|  | Cross-over | No | No | No |
|  | Intentionally unbalanced | No | No | No |
|  | Interventions target different diseases | No | No | No |
|  | Other |  |  |  |
| Form of statistical analysis to deal with factorial design (e.g. inside-the-table): including type of regression model if appropriate | For primary clinical outcome | At-the-margins | At-the-margins | Inside-the-table and at-the-margins co-primary analyses |
|  |  | Interaction test conducted as secondary analysis | N/A | N/A |
|  |  | Comparisons reported in separate paper. Main clinical paper on dalteparin vs. placebo [49] conducted purely at-the-margins with no mention of interaction (although this analysis was done solely on patients with non-invasive treatment). Main clinical paper on intensive vs. non-intensive [48] was done at-the-margins for primary analysis, but also evaluates interactions in secondary analyses (using logistic regression) and presents some results inside-the-table. | Protocol-specified comparisons were L vs. no L, nitrates vs. no nitrates and N+L vs. neither | "2-way" analysis done first, then 4-way analysis comparing 3 active arms with control and combination therapy with 2 monotherapy arms. Analysis done by KM survival curves and Cox regression. Multivariate analyses with interaction terms also investigated. |
|  | Base case economic evaluation | At-the-margins | At-the-margins | Inside-the-table (treating each cell separately) |
|  |  | No mention of interaction term | N/A |  |
|  |  |  |  | Although the paper covers only n3 PUFA not vitamin E, base case results use the results of 4-way comparison as the study was designed to give a comparison between the 4 groups |
|  | Sensitivity analyses of economic evaluation |  | none | At-the-margins |
| Presentation of economic results | | Results for each comparison presented in separate papers with very little mention of the other comparison, although this weakness was pointed out in a letter by Moller et al [47]. | Present at-the-margins comparison of costs and efficacy for lisinopril vs. no lisinopril | Costs for n3 PUFA and control presented for both 2-way and 4-way analysis |
| Presentation of uncertainty (e.g. pair-wise CEAC, EVPI) | | Calculate 95% CI around cost difference, but give no indication of uncertainty around ICERs. | Present 95% around the ICER that are based only on the 95% CI for lives saved, taking no account of costs. | Present CIs around ICERs and life-years but not costs. No clear indication of how 95% CI were derived and no CEACs |
| Statistical significance of differences of each comparison individually | | No significant difference in clinical outcomes or costs between dalteparin and placebo at 3 months. Invasive treatment significantly reduced incidence of MI or death combined at 6 months and 1 year and significantly increased costs at 1 year. | Lisinopril significantly reduced events compared with no lisinopril. Nitrates had no signifiant effect. | n3-PUFA significantly reduced events based on both 2-way and 4-way analysis. Vitamin E had no significant effect |
| Significance of interaction effect | | No significant interaction observed in sample of pts included in invasive vs. non-invasive treatment. Interactions not assessed (and cannot be assessed) in the sample included in the main paper on dalteparin vs. placebo. | Not stated | Not significant, but relatively large: RR for PUFA vs. placebo was 10% by 2-way analysis and 15% by 4-way analysis |
| Results of comparing different methods for statistical analysis | | N/A | None | Results differ by about 50% between inside-the-table and at-the-margins analysis (both in terms of relative risks and ICERs), though this wouldn't change the conclusions at a 40,000 Euro/QALY ceiling ratio |

| **Author/study acronym** | | **Halpern et al 2002 [54]** | **Hollis 2007 [55]** | **HPS [56-61]** |
| --- | --- | --- | --- | --- |
| Year of publication for earliest paper presenting ***methods or results*** of economic evaluation | | 2002 | 2007 | 2005 |
| Year of publication for earliest paper presenting ***methods*** of economic evaluation | | 2002 | 2007 | 2005 |
| Year of publication for earliest paper presenting ***results*** of economic evaluation | | 2002 | 2007 | 2005 |
| Type of factorial design (e.g. partial/fractional) | | full factorial | Full factorial | Full factorial |
| Total sample size (across all arms) | | 1200 | 4614 | 20,536 |
| Size of factorial matrix (e.g. 2x2) | | 2x2x2 | 2x3 | 2x2 |
| Disease area | | Physicians | Smoking cessation | Cardiovascular disease |
| Sponsor | | Manufacturer | US public funding body | MRC |
| Country | | US | US | UK |
| Treatments (or treatment combinations) compared | | Factor 1: $5 vs. $10 incentive mailed in envelope with questionnaire. Factor 2: large envelope vs. standard small envelopes. Factor 3: inclusion of a peppermint sweet vs. no sweet. | Factor 1: Brief, moderate or intensive telephone counselling. Factor 2: offer of free nicotine replacement therapy (NRT) patches. | Factor 1: Simvastatin vs. placebo. Factor 2: vitamin supplement vs. placebo. |
| Type of comparisons | | A0 vs. AX vs. B0 vs. BX (Factor 1: interventions A and B; Factor 2: X vs. 0) | A0 vs. AX vs. B0 vs. BX (Factor 1: interventions A and B; Factor 2: X vs. 0) | 0 vs. a vs. b vs. ab |
| Type(s) of treatment evaluated | | Logistical intervention targetted at health service | Pharmaceutical + other | Pharmaceutical + nutritional supplement |
| Power calculation allowed for interaction effect and/or powered to test for interaction? | | Appears to have been powered to detect large interactions | Not stated | Interactions not mentioned in any publication. However, study was highly powered to detect significant effects for each factor in patient subgroups, so is likely to have been powerful enough to detect moderate interaction effects. |
| Type of economic evaluation (e.g. CUA) | | CEA | CEA | CEA |
| Other notable aspects of study design | Cluster randomised | No | No | No |
|  | Cross-over | No | No | No |
|  | Intentionally unbalanced | yes | Yes | No |
|  | Interventions target different diseases | No | No | No |
|  | Other |  |  |  |
| Form of statistical analysis to deal with factorial design (e.g. inside-the-table): including type of regression model if appropriate | For primary clinical outcome | Logistic regression | Unclear | At-the-margins |
|  |  | Not stated | No mention of interaction term | No mention of interaction term |
|  |  | Used chi squared tests to evaluate main effects and logistic regression to evaluate two-way interactions between main effects | "We used multiple logistic regression to compare the moderate and intensive interventions to brief intervention, the NRT offer to no NRT offer and an NRT offer with the moderate compared to intensive arms." In results, present OR for NRT vs. no NRT and for moderate and intensive vs. brief+NoNRT, but also refer to moderate*NRT and intensive*NRT interaction terms | Log-rank tests used for primary endpoints. No explicit comments on how interactions will be investigated, but does comment that secondary analyses will assess treatmetn effects of each therapy with/without the other (1999) |
|  | Base case economic evaluation | At-the-margins | Inside-the-table (treating each cell separately) | Event-based CEA |
|  |  |  |  | No interaction term included |
|  |  |  | Compared each arm with brief counselling and no NRT, but used OLS to calculate mean total costs and SD for each arm | BMJ 2006: event-based analysis using regression equations predicting event rates, cost of events and life expectancy within a Markov model. Made no allowance for vitamin treatment |
|  | Sensitivity analyses of economic evaluation | None |  | Other |
|  |  |  |  | Lancet 2005: Effect of statins on incidence and cost of vascular events assumed to be proportional and drug costs assumed to be additive. Vitamin allocation ignored; study treated as 2-arm trial on statins |
| Presentation of economic results | | Present costs and ICERs for $10 incentive vs. $5 incentive, based on costs and effects averaged over all other comparisons. Also presented ACERs (total cost/total responses) for $5 and $10. | Present ICERs for each arm vs. brief+NoNRT arm | Presented costs, effects and cost-effectiveness for statin vs. non-statin overall and in subgroups. |
| Presentation of uncertainty (e.g. pair-wise CEAC, EVPI) | | Present 95% CI around ICERs based on bootstrapping | Present "ranges" for each ICERs "calculated using standard deviations and 12-month abstinence" | 95% CI and extensive subgroup analyses but no CEACs |
| Statistical significance of differences of each comparison individually | | $10 incentive significantly increased response rate vs. $5 incentive, but envelope size and mints had no significant effect. | NRT, intensive counselling and moderate counselling showed significant benefits | Vitamins had no significant effects on vascular events or mortality, whereas statins significantly reduced all vascular events and deaths |
| Significance of interaction effect | | However, since either a large envelope OR a mint increases postal costs, but having both rather than just 1 has no effect on postage cost, there is authomatically an interaction for cost | Not significant for primary endpoint. Not reported for other endpoints | Not significant on multiplicative scale (χ-squared for "heterogeneity" between pts in the other allocation=0.03) |
| Results of comparing different methods for statistical analysis | | N/A | N/A | N/A |

| **Author/study acronym** | | **NC STRIDES [62]** | **NIMH-MTA [63-66]** | **PREVEND IT [67, 68]** |
| --- | --- | --- | --- | --- |
| Year of publication for earliest paper presenting ***methods or results*** of economic evaluation | | 2009 | 2005 | 2006 |
| Year of publication for earliest paper presenting ***methods*** of economic evaluation | | 2009 | 2005 | 2006 |
| Year of publication for earliest paper presenting ***results*** of economic evaluation | | 2009 | 2005 | 2006 |
| Type of factorial design (e.g. partial/fractional) | | Full factorial | Full factorial | Full factorial |
| Total sample size (across all arms) | | 825 | 579 | 864 |
| Size of factorial matrix (e.g. 2x2) | | 2x2 | 2x2 | 2x2 |
| Disease area | | Healthy eating in general public and colorectal cancer survivors | ADHD | Prevention of cardiovascular disease; recruited patients with albuminuria |
| Sponsor | | US public funding body | US public funding body | Manufacturer and other funding body |
| Country | | US | US | Netherlands |
| Treatments (or treatment combinations) compared | | Factor 1: tailored print communications (TPC) vs. control. Factor 2: brief telephone-based motivational interviewing (TMI) vs. control. | Factor 1: intensive medication management vs. not. Factor 2: multi-component behavioural treatment vs. not | Factor 1: Fosinopril vs. placebo. Factor 2: pravastatin vs. placebo |
| Type of comparisons | | 0 vs. a vs. b vs. ab | 0 vs. a vs. b vs. ab | 0 vs. a vs. b vs. ab |
| Type(s) of treatment evaluated | | Other | Pharmaceutical + other | Pharmaceutical |
| Power calculation allowed for interaction effect and/or powered to test for interaction? | | No power calculation | Yes: powered based on inside-the-table comparisons | Not stated. Wording of sample size calculation implies that sample size was based on at-the-margins approach only. |
| Type of economic evaluation (e.g. CUA) | | CEA | CEA | CEA |
| Other notable aspects of study design | Cluster randomised | No | no | No |
|  | Cross-over | No | no | No |
|  | Intentionally unbalanced | No | no | No |
|  | Interventions target different diseases | No | no | No |
|  | Other | This study is also stratified by cancer status, since some pts were colorectal cancer survivors and others were matched controls. | In the combined treatment group, the two treatments were integrated by information sharing between teacher-consultant and pharmacotherapist that was used to guide overall decisions. As result, combination treatment was not the simple addition of the two unimodal treatments | RCT nested within an observational study. Economic evaluation is partly model-based and evaluates the cost-effectiveness of screening for albuminuria and treatment with fosinopril, rather than explicitly being limited to the comparisons in the factorial design. |
| Form of statistical analysis to deal with factorial design (e.g. inside-the-table): including type of regression model if appropriate | For primary clinical outcome | Linear regression/ANCOVA/ANOVA | Linear regression/ANCOVA/ANOVA | At-the-margins |
|  |  | No mention of interaction term | Interaction term included regardless of significance | Not stated |
|  |  | Not clearly stated whether both interventions were considered in the same model. Results section appears to be based on pair-wise comparisons between each of the 3 active treatments and control | "When omnibus RR [random-effects regression] analyses comparing all 4 groups were significant, 3 sets of pairwise comparisons were performed, each set addressing 1 of the principal study questions: (1) medication management vs. behavioral treatment (2 tailed); (2) combined treatment vs. medication management and combined treatment vs. behavioral treatment (1 tailed, assuming the superiority of combined treatment); and (3) community care vs. medication management, community care vs. behavioral treatment, and community care vs. combined treatment (1 tailed, assuming the superiority of MTA treatments)." Adjusted for multiple comparisons |  |
|  | Base case economic evaluation | Inside-the-table (treating each cell separately) | Inside-the-table (treating each cell separately) | At-the-margins |
|  |  | Present pair-wise comparisons between arms. Methods used unclear |  |  |
|  | Sensitivity analyses of economic evaluation | Not stated | None | None |
|  |  |  | No sensitivity analyses compared different ways of dealing with factorial design |  |
| Presentation of economic results | | Present pair-wise comparisons between arms. Methods used unclear | Present costs and outcomes separately for each group | Presented costs, effects and cost-effectiveness for screen and treat with fosinopril vs. no screening or treatment |
| Presentation of uncertainty (e.g. pair-wise CEAC, EVPI) | | Unclear: no explicit mention of uncertainty, although present table with a column entitled "Cost-effective" with values between 0.822-0.992 that might be probability that treatment is cost-effective compared with a single comparator, although it is impossible to tell since these values are not mentioned in the paper. | Uncertainty dealt with using bootstrapping. Jensen et al show CE plane with separate clusters for each of the 4 groups. Foster et al show CEACs for multiple comparisons with the 4 strategies shown, based on bootstrapping and show NHB and 95% CI for each arm based on different outcome measures. | Presented pair-wise CEACs and CE planes for screen and treat with fosinopril vs. no screening or treatment |
| Statistical significance of differences of each comparison individually | | Group receiving TPC+TMI produced significant improvements for fruit and vegetable consumption but not exercise; it is unclear whether this conclusion is based on comparison with baseline or between groups | Medication and combined groups showed significantly greater improvement than behavioural and community care groups. Combined treatment was not significantly better than medication alone | Fosinopril showed significant benefits, although pravastatin did not lower risk of CV mortality significantly. |
| Significance of interaction effect | | Not stated. | Not stated | Not stated |
| Results of comparing different methods for statistical analysis | | N/A | N/A | N/A |

| **Author/study acronym** | | **Richards [21, 69, 70]** | **Rodewald [71]** | **Saillour-Glenisson et al 2005 [72]** |
| --- | --- | --- | --- | --- |
| Year of publication for earliest paper presenting ***methods or results*** of economic evaluation | | 2006 | 1999 | 2005 |
| Year of publication for earliest paper presenting ***methods*** of economic evaluation | | 2006 | 1999 | 2005 |
| Year of publication for earliest paper presenting ***results*** of economic evaluation | | 2006 | 1999 | 2005 |
| Type of factorial design (e.g. partial/fractional) | | Full factorial | Full factorial | Full factorial |
| Total sample size (across all arms) | | 6133 | 3015 | 1306 |
| Size of factorial matrix (e.g. 2x2) | | 2x2 | 2x2 | 2x2 |
| Disease area | | Breast cancer screening | Complete birth cohort | Thyroid tests |
| Sponsor | | MRC | US public funding body | Other public funding body |
| Country | | UK | US | France |
| Treatments (or treatment combinations) compared | | Factor 1: letter from GP reminding about screening vs. no letter. Factor 2: flag in women's notes prompting discussion of screening, vs. no flag. | Factor 1: tracking with outreach to bring underimmunised children to primary care provider. Factor 2: prompting (primary care office policy change to identify and reduce missed immunisation opportunities | Factor 1: pocket card aide memoir [PAM] vs. no card. Factor 2: test request form giving information on when thyroid tests are necessary vs. no form [FPC] |
| Type of comparisons | | 0 vs. a vs. b vs. ab | 0 vs. a vs. b vs. ab | 0 vs. a vs. b vs. ab |
| Type(s) of treatment evaluated | | Logistical intervention targetted at health service | Logistical intervention targetted at health service | Logistical intervention targetted at health service |
| Power calculation allowed for interaction effect and/or powered to test for interaction? | | Not stated. Comment that the interaction was investigated with "only limited power" | No power calculation conducted | Unclear. Comments that sample size calculations took into consideration the comparision of the efficacy of FPC and PAM with respect to the control group, although interactions were not explicitly mentioned |
| Type of economic evaluation (e.g. CUA) | | CEA and CUA | CEA | CEA |
| Other notable aspects of study design | Cluster randomised | Yes | No | Yes |
|  | Cross-over | No | No | No |
|  | Intentionally unbalanced | No | No | No |
|  | Interventions target different diseases | No | No | No |
|  | Other |  |  | FPC was cluster-randomised at the level of hospitals, while PAM was cluster-randomised at the level wards |
| Form of statistical analysis to deal with factorial design (e.g. inside-the-table): including type of regression model if appropriate | For primary clinical outcome | Logistic regression | Linear regression/ANCOVA/ANOVA | Logistic regression |
|  |  | No interaction term included | Not stated | Interaction term excluded as it was not statistically significant |
|  |  | "the interaction between the two interventions was also investigated, although with only limited power." Interaction terms included in regression model in a secondary analysis. Table 5 states that the OR for main effects are based on a model that did not contain an interaction term. | "Two-way ANOVA was used to test for effects of each of the interventions on the outcome measures, and for interactions between the interventions." no clear indication of whether interaction terms were included in the base case analysis | An interaction between FPC and PAM was evaluated. In the absence of an interaction, the proper effect of each intervention was studied using a "generalised linear mixed model" to account for cluster randomisation and stratification by pair of hospitals, although odds ratios were presented, implying that logistic regression was used. Results text implies that the efficacy of each intervention was based on simple effects (i.e. a comparison between cells A0 and 00), although Table II suggests that treatment effects were based on logistic regression with no interaction term |
|  | Base case economic evaluation | Logistic regression | Unclear | Inside-the-table (treating each cell separately) |
|  |  | Interaction term included regardless of significance | Not stated |  |
|  |  | Logistic regression model in WinBUGS, adjusting for clustering by practice and an interaction term. In the original publication (Brown 2006), the interaction term was assigned an informative prior that it is unlikely (with 95% probability) to exceed the magnitude of the main effects. The logistic regression model estimated the probability of attending screening for each patient and the cost of screening and the cost of the intervention was added on (allowing for the uncertainty around the cost parameters). | Appear to have taken the total cost per child of the outreach intervention and divided it by the "main effect for tracking/outreach in the ANOVA results". No estimation of uncertainty around this figure. Unclear whether the main effect in question was from an analysis with or without interaction terms. | ICERs were calculated relative to cell 00 (neither FPC nor PAM) on a inside-the-table basis. |
|  | Sensitivity analyses of economic evaluation | Various | None | None |
|  |  | 1) Economic evaluation in primary clinical paper presented only point estimate for ICER (mean incremental cost/mean incremental effect) for flag or letter vs. control. (2) Welton et al estimated EVPPI prior to the start of the Richards study. (3) Welton et al estimated outcomes with an informative, evidence-based prior around treatment effect. (4) Welton et al estimated outcomes with no interaction. |  |  |
| Presentation of economic results | | Brown et al presented incremental cost and additional numbers of attendences for each active treatment arm vs. control group and presented incremental outcomes along the frontier, although the original paper gave only CER vs. control. | Cost-effectiveness only estimated for outreach/tracking vs. no outreach/tracking. Simply estimated point estimates for the ICERs by dividing costs by incremental benefits. | Present incremental cost relative to cell 00 and total and incremental outcomes; ICERs calculated relative to cell 00 |
| Presentation of uncertainty (e.g. pair-wise CEAC, EVPI) | | Presented incremental costs & effects vs. control on CE and CEAC frontier and EVPI allowing for multiple comparisons. These were calculated in WinBUGS. EVPPI estimated by Welton et al (both before and after the trial was conducted) | None | No presentation of uncertainty around economic results besides showing heterogeneity between types of hospitals |
| Statistical significance of differences of each comparison individually | | Both flag and letter had a significant effect | Tracking/outreach had significant effect. Prompting "did not have a measurable effect" | FPC showed significantly superiority, but PAM had no significant effect |
| Significance of interaction effect | | No significant interaction. "The findings ruled out any marked disadvantage from using both interventions simultaneously." | No significant interaction | No significant interaction for clinical outcome (p=0.21) |
| Results of comparing different methods for statistical analysis | | ICER for letter vs. control was £27/attendance in Welton analysis that allowed for uncertainty and included interaction term, vs. £26/attendance in original clinical paper. By taking an incremental analysis, Brown et al concluded that the flag was inefficient (being dominated by letter), whereas the primary clinical paper concluded only that flag was less cost-effective than the letter based on CERs for each intervention vs. control. The difference was even more stark for the comparison between flag+letter and letter alone, which was £171/attendance in Welton et al and £41/attendance in the clinical paper. In the additional analyses presented by Welton et al (Table 4), the total NMB of the 4 interventions differs depending on whether conclusions are based on evidence before the Richards study, the evidence after Richards study with an evidence-based prior, or whether or not an interaction effect was included in an analysis after Richards study with a vague prior for main effect. | N/A | N/A |
| **Author/study acronym** | | **Salize/Twardella [73, 74]** | **SAPPHIRE [75, 76]** | **Thomas et al [77, 78]** |
| Year of publication for earliest paper presenting ***methods or results*** of economic evaluation | | 2009 | 2009 | 2005 |
| Year of publication for earliest paper presenting ***methods*** of economic evaluation | | 2009 | 2009 | 2005 |
| Year of publication for earliest paper presenting ***results*** of economic evaluation | | 2009 | 2009 | 2005 |
| Type of factorial design (e.g. partial/fractional) | | full factorial | Partial factorial | Incomplete factorial |
| Total sample size (across all arms) | | 577 | 200 | 786 |
| Size of factorial matrix (e.g. 2x2) | | 2x2 | 2x2 | 2x2x2 |
| Disease area | | Smoking cessation | Shoulder pain | Knee pain |
| Sponsor | | Other public funding body | MRC | Other UK public funding body |
| Country | | Germany | UK | UK |
| Treatments (or treatment combinations) compared | | Factor 1: Training and incentive (TI) for GPs. Factor 2: Training and medication (TM: I.e. GPs were trained and had opportunity to prescribe NRT or buproprion at no cost to patient). Control group appear to have received no training. | Factor 1 (cluster randomised): GP training vs. no training. Factor 2 (individually randomised): lignocaine or cortisone | Factor 1 (all pts): exercise programme vs. no exercise programme. Factor 2 (all pts): Monthly telephone contact giving advice on pain management. Factor 3 (00 and AB groups only): placebo health food tablet vs. no placebo. |
| Type of comparisons | | 0 vs. a vs. b vs. ab | A0 vs. AX vs. B0 vs. BX (Factor 1: interventions A and B; Factor 2: X vs. 0) | 0 vs. a vs. b vs. ab |
| Type(s) of treatment evaluated | | Pharmacological + logistical intervention targetted at health service | Pharmaceutical + other | Physiotherapy and related techniques |
| Power calculation allowed for interaction effect and/or powered to test for interaction? | | Unclear. Comment that "power calculations were based on the expected point prevalence of abstinence of 5% in usual care, 10% in interventions TI and TM, and 15% in the intervention TI+TM in a 12-month follow-up", which would imply no interaction on additive scale, but a large super-additive interaction on the logarithmic scale that was used to analyse results. Interactions not mentioned. | Not stated, although discussion of sample size considerations implies that separate power calculations were conducted for each factor | Not stated |
| Type of economic evaluation (e.g. CUA) | | CEA | CUA | CEA |
| Other notable aspects of study design | Cluster randomised | yes | Cluster-randomised for 1 factor but not all | No |
|  | Cross-over | no | No | No |
|  | Intentionally unbalanced | no | No | yes |
|  | Interventions target different diseases | no | No | No |
|  | Other | Since it appears that no training was given to the control group and the TI+TM group appear to have only attended one training session, this study has confounded the interaction term and therefore cannot assess interactions and it is debatable whether it is truly factorial |  |  |
| Form of statistical analysis to deal with factorial design (e.g. inside-the-table): including type of regression model if appropriate | For primary clinical outcome | Logistic regression | At-the-margins | Linear regression/ANCOVA/ANOVA |
|  |  | No mention of interaction term | Interaction term excluded as it was not statistically significant | Interaction term excluded as it was not statistically significant |
|  |  | Used logistic regression, adjusting for clustering. Present OR for TI vs. not TI and TM vs. not TM | "The first analysis was tested for an interaction between substance and training. An interaction term between substance and training was added to the model. This interaction was not significant, hence the analysis focused on two separable trials, training vs. no training and lignocaine vs. cortisone." | Authors decided to present main effects not simple main effects as interaction was significant only at 6 months. |
|  | Base case economic evaluation | Inside-the-table (treating each cell separately) | Linear regression/ANCOVA/ANOVA | At-the-margins |
|  |  |  | No interaction term included |  |
|  |  |  | Used seemingly-unrelated regression to allow for clustering, but conducted analyses on the two factors separately, without controlling for the other factor or including interaction terms. Bayesian analsyis in MLwiN. "As it would not be expected that whether or not a GP is trained in delivering a shoulder injection would impact on the relative cost effectiveness of alternative drugs (which is supported by the absence of statistically significant interactions between treatment and training [10]), we compared treatment separately from GP training" | Presented cost-effectiveness only for with/without exercise intervention |
|  | Sensitivity analyses of economic evaluation | None | None | None |
| Presentation of economic results | | Presented ICERs for TM vs. control and TI+TM vs. control and TI+TM vs. TM. Excluded TI arm as the prevalence of abstinence did not differ significantly from that in control arm | Compared costs and effects between all trained vs. all not trained and for all lignocaine vs. all cortisone; results for individual cells not presented | Present detailed costs and cost-effectiveness for exercise vs. no exercise. Present total cost of telephone intervention, but no detailed information or ICERs |
| Presentation of uncertainty (e.g. pair-wise CEAC, EVPI) | | Pair-wise CEACs for TM vs. control and TI+TM vs. control and TI+TM vs. TM (based on bootstrapping) | Presented pair-wise CEACs for training vs. no training and for lignocaine vs. cortisone, but not multiple comparison CEACs | Pair-wise CEACs calculated using bootstrapping |
| Statistical significance of differences of each comparison individually | | TI had no significant effect, but TM significantly increased the odds of cessation | No significant difference between lidocaine and cortisone or between training and no training | Exercise showed significant improvement. Telephone counselling and placebo had no significant effect |
| Significance of interaction effect | | Not stated | Not significant | Significant quantitative interaction was observed between exercise and telephone contact at 6 months, but not at any other timepoint; this isolated significant interaction was interpreted as being due to chance |
| Results of comparing different methods for statistical analysis | | N/A | N/A | N/A |

| **Author/study acronym** | | **Thompson et al 1986 [79]** | **TIMI 3B [80, 81]** | **Tjan-Heijnen et al [82, 83]** |
| --- | --- | --- | --- | --- |
| Year of publication for earliest paper presenting ***methods or results*** of economic evaluation | | 1986 | 2003 | 2003 |
| Year of publication for earliest paper presenting ***methods*** of economic evaluation | | 1986 | 2003 | 2003 |
| Year of publication for earliest paper presenting ***results*** of economic evaluation | | 1986 | 2003 | 2003 |
| Type of factorial design (e.g. partial/fractional) | | Full factorial | full factorial | full factorial |
| Total sample size (across all arms) | | 507 | 1473 | 163 |
| Size of factorial matrix (e.g. 2x2) | | 2x2x2 + 2 | 2x2 | 2x2 |
| Disease area | | Colorectal cancer screening | Angina and myocardial infarction | Lung cancer |
| Sponsor | | US public funding body | US public funding body | Manufacturer |
| Country | | US | US and Canada | Europe |
| Treatments (or treatment combinations) compared | | Factor 1: Physician talk about test vs. nurse talk vs. no talk; the nurse talk comparison was added later and not fully crossed with others, such that double combinations of nurse talk were not considered. Factor 2: Reminder postcard vs. no postcard; Factor 3: Telephone follow-up to those not returning test in 10 days vs. no telephone follow-up. | Factor 1: Tissue plasminogen activator (TPA) vs. placebo. Factor 2: early invasive strategy (early coronary arteriography then revascularisation when appropriate) vs. early conservative strategy (coronary arteriography then revascularisation after initial medical therapy) | Factor 1: standard triple chemotherapy (CDE) vs. intensified CDE + G-CSF. Factor 2: antibiotics (ciprofloxacin+roxithromcyin) vs. placebo. |
| Type of comparisons | | 0 vs. a vs. b vs. ab | A0 vs. AX vs. B0 vs. BX (Factor 1: interventions A and B; Factor 2: X vs. 0) | A0 vs. AX vs. B0 vs. BX (Factor 1: interventions A and B; Factor 2: X vs. 0) |
| Type(s) of treatment evaluated | | Logistical intervention targetted at health service | Pharmaceutical and surgery | Pharmaceutical |
| Power calculation allowed for interaction effect and/or powered to test for interaction? | | No power calculation | Not stated, although wording implies that power calculation was based on at-the-margins analysis and the study was not powered for subgroup analyses unless there were large differences | Not powered to detect interaction, although unclear whether this is because antibiotics vs. placebo comparison stopped early on ethical grounds. |
| Type of economic evaluation (e.g. CUA) | | CCA | CEA | CEA |
| Other notable aspects of study design | Cluster randomised | no | No | No |
|  | Cross-over | no | No | No |
|  | Intentionally unbalanced | no | No | No |
|  | Interventions target different diseases | no | no | yes |
|  | Other |  |  |  |
| Form of statistical analysis to deal with factorial design (e.g. inside-the-table): including type of regression model if appropriate | For primary clinical outcome | Inside-the-table (treating each cell separately) | At-the-margins | At-the-margins |
|  |  |  | Interaction term excluded as it was not statistically significant |  |
|  |  | Results are presented inside-the-table, although at-the-margins analysis is used to test overall effect of physician talk and adjusted binary regression is also conducted (which appears to have been evaluated with dummies for each of the 9 active treatment groups) | Two-stage testing procedure: tested for interaction using Breslow-Day test; "If treatment by strategy interaction was not detected for the initial therapy end point, results were pooled across the two strategies to provide a single TPA vs. placebo group comparison. Likewise, if a treatment by strategy interaction was not detected for the strategy primary end point, results were pooled across the two treatment assignments (TPA and placebo) to provide a single early invasive strategy vs. early conservative strategy comparison. | Present both at-the-margins analysis and logistic regression (with interaction term omitted as it was not significant) |
|  | Base case economic evaluation | At-the-margins | Logistic regression | At-the-margins |
|  |  | No interaction term included | Unclear |  |
|  |  | Compare crude costs of all 4 interventions; conclude that postcard is best as it is cheapest and gives comparable results to other arms. Conduct more detailed costing analysis to assess incremental cost of postcard vs. no postcard (including savings from early detection) and show postcard to be cost-saving (and therefore dominant). | Logistic regression model was used to explore how outcomes and treatment effects varied between risk groups; economic evaluation focussed only on invasive vs. conservative comparison and is highly unlikely to have included interactions between treatment strategies |  |
|  | Sensitivity analyses of economic evaluation | None | None |  |
|  |  |  |  |  |
| Presentation of economic results | | see above | Presented only incremental outcomes and costs for invasive and conservative management (at-the-margins presentation) | Simply compare costs and effects for antibiotics vs. placebo for Germany and Netherlands, concluding that antibiotics dominate placebo |
| Presentation of uncertainty (e.g. pair-wise CEAC, EVPI) | | None | None | threshold and 3-way sensitivity analyses |
| Statistical significance of differences of each comparison individually | | Postcard and phone call alone are significantly better than control; when analysed at-the-margins, physician talk significantly increases compliance, with no signficant difference between nurse and physician talk. | No significant difference in clinical endpoints for either factor, although TPA-treated patients had significantly more Mis | Antibiotic chemotherapy significantly reduced incidenceof febrile leucopaenia; results for standard vs. intensive chemo not yet reported |
| Significance of interaction effect | | Not stated; authors conclude effect is additive and that all treatments have equal effect based on comparison of crude compliance data. | Not significant | Non-significant qualitative interaction for incidence of febrile leukopenia (p=0.130) inferred from table 2, not discussed in paper. |
| Results of comparing different methods for statistical analysis | | N/A | None | N/A |

| **Author/study acronym** | | **UK BEAM [84, 85]** | **UKPDS [86-95]** |
| --- | --- | --- | --- |
| Year of publication for earliest paper presenting ***methods or results*** of economic evaluation | | 2004 | 1998 |
| Year of publication for earliest paper presenting ***methods*** of economic evaluation | | 2004 | 1998 |
| Year of publication for earliest paper presenting ***results*** of economic evaluation | | 2004 | 1998 |
| Type of factorial design (e.g. partial/fractional) | | Full factorial | Partial factorial |
| Total sample size (across all arms) | | 1,334 | 4,209 |
| Size of factorial matrix (e.g. 2x2) | | 2x3 | 4x3x2 |
| Disease area | | Low back pain | Diabetes and cardiovascular disease |
| Sponsor | | MRC | Various public and private organisations |
| Country | | UK | UK |
| Treatments (or treatment combinations) compared | | Factor 1: spinal manipulation in NHS clinic, spinal manipulation in private clinic, or best care with GP (no manipulation). Factor 2: exercise programme, vs. best care. | **Factor 1 (UKPDS):** Diet vs. insulin vs. sulphonylurea (vs. metformin in obese patients only); patients randomised to diet were randomised to active treatments if diet failed; patients randomised to sulphonylurea were randomised to receive chlorpropamide rather than glibenclamide (centres started pre-1987) or glipizide (centres started 1987-8); additional randomisations for 2nd line or add-in therapy made later on. **Factor 2 (HDS, hypertensive pts only):** Tight BP control (pts randomised to captopril OR atenolol) or less tight BP control. **Factor 3 (UKPDS Acarbose Study)**: Acarbose vs. placebo (in 1946 patients) |
| Type of comparisons | | 0 vs. a vs. b vs. ab | Other |
| Type(s) of treatment evaluated | | Physiotherapy and related techniques | Pharmaceutical |
| Power calculation allowed for interaction effect and/or powered to test for interaction? | | Not stated | Not stated |
| Type of economic evaluation (e.g. CUA) | | CUA | CEA and CUA |
| Other notable aspects of study design | Cluster randomised | No | No |
|  | Cross-over | no | No |
|  | Intentionally unbalanced | yes | Yes |
|  | Interventions target different diseases | No | No |
|  | Other | 1:1:1:1 randomisation between GP care, exercise, manipulation and manipulation+exercise, with 50% of the pts randomised to the latter 2 categories being allocated to private rather than NHS care | Additional randomisations within different cells of the factorial design |
| Form of statistical analysis to deal with factorial design (e.g. inside-the-table): including type of regression model if appropriate | For primary clinical outcome | Linear regression/ANCOVA/ANOVA | At-the-margins |
|  |  | Interaction term included as reached statistical significance | No mention of interaction term |
|  |  | "We analysed the data in steps. Firstly, we used multilevel modelling to make allowance for the innate clustering of participants by centre, exercise class, manipulator, and practice. Secondly, we tested the effect of exercise without manipulation by comparing participants allocated to best care with those allocated to best care plus exercise (table 2). Thirdly, we tested the effect of manipulation without exercise by comparing participants allocated to best care with those allocated to best care plus manipulation (table 3). If this was significant, we tested for differences between manipulation in NHS and private premises. Finally, if either exercise or manipulation gave significant results, we tested for interactions between exercise and manipulation—that is, whether the estimated improvement in participants allocated to best care, manipulation, and exercise (table 4) differed significantly from the sum of the estimated improvement due to manipulation (table 3) and that due to exercise (table 2)." | For Factors 1 & 2: Cox regression used in papers 33, 34, 38 and 39, with each comparison analysed separately. For Factor 3: mean outcomes across factor 1 groups (at-the-margins) and results disaggregated by factor 1 allocation (inside-the-table) are both presented. |
|  | Base case economic evaluation | Other | At-the-margins |
|  |  | Interaction term included as reached statistical significance | No mention of interaction term |
|  |  | "Because the accompanying clinical paper found statistically significant interactions between manipulation and exercise,1 it compares four distinct treatments within the factorial design.19 Although costs show no interaction between treatments, this paper also compares these four treatments for three reasons. Firstly, as costs vary much more than clinical outcomes, this is prudent. Secondly, those people responsible for allocating resources need to choose between these four treatments. Finally, this epitomises the Bayesian statistical approach adopted in this paper." Private vs. NHS manipulation comparison is ignored; in base case, all pts were assumed to have NHS care. Adjusted for clustering in economic evaluation using bivariate multi-level Bayesian analysis in MLwiN | 40, 41, 51 compared mean costs & QALYs and calculated ICERs separately for a single comparison, ignoring all others. |
|  | Sensitivity analyses of economic evaluation |  | At-the-margins, including event-based CEA conducted at-the-margins |
|  |  | Conducted a sensitivity analysis using private costs for manipulation and one in which 50% of pts were assumed to use private premises | Various sensitivity analyses presented, although these all appear to be conducted at-the-margins |
| Presentation of economic results | | NHS vs. private care comparison is ignored. Costs and effects presented for the 4 remaining arms in a inside-the-table approach. CE frontier was identified and ICERs calculated vs. next most costly treatment on frontier | 40, 41, 51 reported mean costs QALYs and ICER separately for a single comparison, ignoring all others. 72 reported mean costs, QALYs and ICERs for each of the 3 main comparisons at-the-margins |
| Presentation of uncertainty (e.g. pair-wise CEAC, EVPI) | | Presented CEACs for multiple comparisons and those for manipulation alone vs. best care and for exercise vs. best care | CEACs for pair-wise comparisons |
| Statistical significance of differences of each comparison individually | | Exercise significantly superior to no exercise. Manipulation significantly superior to no manipulation. No significant difference between NHS and private manipulation | Intensive glucose control significantly reduced diabetic events in non-overweight (33) and overweight (34) patients. Tight BP control significantly reduced diabetic events (38). Captopril and atenolol were equally effective (39). Acarbose significantly reduced HbA1c (44). |
| Significance of interaction effect | | Significant negative interaction between manipulation and exercise at 3 months, but not at 12 months. No interaction observed between treatments was observed for costs. | Not stated |
| Results of comparing different methods for statistical analysis | | N/A | N/A |

| **Author/study acronym** | | **VIP [96]** | **Wesson et al 2008 [97]** |
| --- | --- | --- | --- |
| Year of publication for earliest paper presenting ***methods or results*** of economic evaluation | | 2005 | 2008 |
| Year of publication for earliest paper presenting ***methods*** of economic evaluation | | 2005 | 2008 |
| Year of publication for earliest paper presenting ***results*** of economic evaluation | | 2005 | 2008 |
| Type of factorial design (e.g. partial/fractional) | | full factorial | Full factorial |
| Total sample size (across all arms) | | 391 | 45 clinics |
| Size of factorial matrix (e.g. 2x2) | | 2x2 | 2x2 |
| Disease area | | Falls in people with visual impairment | Family planning |
| Sponsor | | Other public funding body | Other public funding body |
| Country | | New Zealand | Kenya |
| Treatments (or treatment combinations) compared | | Factor 1: home safety programme vs. no home safety programme. Factor 2: exercise programme vs. no exercise programme. Control group received two social home visits. | Factor 1: Educational outreach intervention ("detailing") for clinic providers vs. no detailing for clinic providers. Factor 2: Detailing for community based distribution agents (CBD) vs. no detailing for CBD |
| Type of comparisons | | 0 vs. a vs. b vs. ab | 0 vs. a vs. b vs. ab |
| Type(s) of treatment evaluated | | Physiotherapy and related techniques | Logistical intervention targetted at health service |
| Power calculation allowed for interaction effect and/or powered to test for interaction? | | Not stated | No - assumed no interaction |
| Type of economic evaluation (e.g. CUA) | | CEA | CEA |
| Other notable aspects of study design | Cluster randomised | No | Yes |
|  | Cross-over | No | No |
|  | Intentionally unbalanced | No | No |
|  | Interventions target different diseases | No | No |
|  | Other |  |  |
| Form of statistical analysis to deal with factorial design (e.g. inside-the-table): including type of regression model if appropriate | For primary clinical outcome | Poisson regression | Linear regression/ANCOVA/ANOVA |
|  |  | Interaction term included as reached statistical significance | Not stated |
|  |  | Since interaction was statistically significant, authors "presented pre-planned combined group comparisons plus appropriate single group comparisons" | Used linear regression although primary outcome was the percentage or absolute number of IUDs implanted. Authors comment that "The interaction effect of detailing at a study site for both target groups and the main effects of detailing at study sites were examined for either providers or CBD agents." No other mention of interactions in the paper |
|  | Base case economic evaluation | At-the-margins | Inside-the-table (treating each cell separately) |
|  |  | Only presented cost-effectiveness for home safety vs. no home safety programme since exercise was not found to be effective. Appear to have based this on the at-the-margins estimate of the efficacy of home safety despite a qualitative interaction (ICER=total cost/difference in total falls with/without home safety programme) | Divided the total cost of the CBD+detailing intervention (plus the cost of the IUDs implanted) by the number of additional IUDs implanted compared with *baseline*. Compared this ICER with that for depot injections. |
|  | Sensitivity analyses of economic evaluation | None | None |
| Presentation of economic results | | Presented only cost of home safety programme and cost/fall prevented. Results for exercise programme were not presented as it was ineffective | Results only presented for CBD+detailing vs. pre-study baseline. |
| Presentation of uncertainty (e.g. pair-wise CEAC, EVPI) | | None besides calculation of SD for cost and one-way sensitivity analyses | None |
| Statistical significance of differences of each comparison individually | | Home safety programme significantly reduced falls. Exercise programme non-significantly increased falls. | Not stated - compared each intervention with baseline |
| Significance of interaction effect | | Statistically significant qualitative interaction for primary endpoint | Not stated |
| Results of comparing different methods for statistical analysis | | For clinical endpoints, there is a very large qualitative interaction that means that exercise programme increases fall rate in presence of home safety programme and reduces it when used alone; as result, at-the-margins analysis finds exercise to be non-significantly harmful, although it reduces falls when used alone. | N/A |

**Table 2.2.** Protocols and terminated studies

| **Author/study acronym** | | **ACCORD [98, 99]** | **AED study [100]** | **CENEX [101, 102]** |
| --- | --- | --- | --- | --- |
| Year of publication for earliest paper presenting ***methods*** of economic evaluation | | 2007 | 2009 | 2007 |
| Protocol or completed study? | | Protocol | Protocol | Protocol |
| Anticipated publication date of ec anal (for protocols only) | | Not stated | Not stated | Not stated |
| Type of factorial design (e.g. partial/fractional) | | Partial factorial | Full factorial | Full factorial |
| Projected total sample size (across all arms) | | 4733 | 1179 | 2800 |
| Size of factorial matrix (e.g. 2x2) | | double 2x2 | 3x3 | 2x2 |
| Disease area | | Diabetes | Alcohol abuse | Nutrition and exercise in older people |
| Sponsor | | US public funding body | Department of Health | Wellcome Trust |
| Country | | US and Canada | UK | Chile |
| Treatments (or treatment combinations) compared | | Factor 1 (all patients): Intensive glycaemic control targetting HbA1c<6.0%, vs. standard glycaemic control targetting HbA1c<7.0-7.9%. Factor 2 differed between groups: For the 5518 pts with moderate dyslipidemia, Factor 2 comprised fenofibrate vs. placebo. For the remaining 4733 pts, Factor 2 comprised intensive blood pressure therapy targeting systolic BP<120 mmHg, vs. standard BP therapy targeting systolic BP<140 mmHg. | Factor 1: screening method (M-SASQ vs. FAST vs. SIPS-PAT questionnaires). Factor 2: intervention (patient information leaflet [PIL]; brief advice [BA]; or brief lifestyle counselling [BLC]) | Factor 1: Nutritional supplement vs. no supplement. Factor 2: exercise vs. no exercise |
| Type of comparisons | | 0 vs. a vs. b vs. ab | A1 vs. A2 vs. B1 vs. B2 (≥2 dosing regimens of ≥2 interventions) | 0 vs. a vs. b vs. ab |
| Type(s) of treatment evaluated | | Pharmaceutical | Other | Nutritional supplement/diet + exercise |
| Power calculation allowed for interaction effect and/or powered to test for interaction? | | Interactions not mentioned in main protocol, although protocol does mention that power calculations on each treatment assumed that the other 2 interventions would achieve the effect sizes for which they were powered | Not stated: comments that: "The sample size calculation is designed to account primarily for intervention level outcomes. Powering the study in this way will also account statistically for appropriate outcomes for screening approach and screening method." | No - assumes no interactions |
| Type of economic evaluation (e.g. CUA) | | CEA and CUA | CEA and CUA | CEA and CUA |
| Other notable aspects of study design | Cluster randomised | No | Yes | Yes |
|  | Cross-over | No | No | No |
|  | Intentionally unbalanced | No | No | Yes |
|  | Interventions target different diseases | No | No | Yes |
| Form of statistical analysis to deal with factorial design (e.g. inside-the-table): including type of regression model if appropriate | For primary clinical outcome | Cox regression | Logistic regression | Not stated |
|  |  | No mention of interaction term | Not stated | No interaction term included |
|  |  |  | Comment that "Due to the nested factorial nature of the study, we will use multilevel modelling to explore potential interactions between each of the levels nested within the trial" |  |
|  | Base case economic evaluation | At-the-margins | Unclear | Not stated |
|  |  | Describe primary hypotheses as "main effects" and describe 3 hypotheses | "The economic analysis will calculate the incremental cost-effectiveness of the control condition with the AHW condition under study" |  |
|  | Sensitivity analyses of economic evaluation | Inside-the-table (treating each cell separately) | Not stated | Not stated |
|  |  | Define two secondary hypotheses comparing AB group with 00 group for each half of the factorial design |  |  |
| Presentation of economic results | | Not stated | Not stated | Not stated |
| Presentation of uncertainty (e.g. pair-wise CEAC, EVPI) | | Plan to use bootstrapping and calculate 95% CI around ICERs | Plan to construct CEACs showing "the probability that the brief intervention is more cost-effective than usual care" | Not stated |

| **Author/study acronym** | | **CJS [103]** | **Coombes [104]** | **IMPAC3T [105]** |
| --- | --- | --- | --- | --- |
| Year of publication for earliest paper presenting ***methods*** of economic evaluation | | 2009 | 2009 | 2007 |
| Protocol or completed study? | | Protocol | Protocol | Protocol |
| Anticipated publication date of ec anal (for protocols only) | | 30 month trial began May 2008 | Not stated |  |
| Type of factorial design (e.g. partial/fractional) | | Full factorial | Full factorial | Full factorial |
| Projected total sample size (across all arms) | | 480 | 132 | 400 patients; 20 practices |
| Size of factorial matrix (e.g. 2x2) | | 2x3 | 2x2 | 2x2 |
| Disease area | | Alcohol abuse | Lateral epicondylagia | Lower respiratory tract infections |
| Sponsor | | Department of Health | Other public funding body | Other public funding body |
| Country | | UK | Australia | Netherlands |
| Treatments (or treatment combinations) compared | | Factor 1: screening method (FAST or M-SASQ questionnaires). Factor 2: intensity of brief intervention (client information leaflet [CIL]; brief advice [BA]; or brief lifestyle counselling [BLC]). | Factor 1: corticosteroid injection vs. placebo. Factor 2: Physiotherapy vs. no physiotherapy | Factor 1: communication skills training for GPs. Factor 2: C-reactive protein test at point of care |
| Type of comparisons | | A1 vs. A2 vs. B1 vs. B2 (≥2 dosing regimens of ≥2 interventions) | 0 vs. a vs. b vs. ab | 0 vs. a vs. b vs. ab |
| Type(s) of treatment evaluated | | Other | Pharmaceutical and physiotherapy | Logistical intervention targetted at health service |
| Power calculation allowed for interaction effect and/or powered to test for interaction? | | Not stated. Comments that "The sample size calculation is designed to account primarily for intervention level outcomes. Powering the study in this way will also account statistically for appropriate outcomes for screening approach and screening method." | No - "The power calculation is based on previous findings that […] the combination of the two will be additive, but not synergistic in effect" | Not stated, although authors comment that interactions can affect power |
| Type of economic evaluation (e.g. CUA) | | CEA and CUA | CUA and CBA | Not stated |
| Other notable aspects of study design | Cluster randomised | Yes | No | Yes |
|  | Cross-over | No | No | No |
|  | Intentionally unbalanced | No | No | No |
|  | Interventions target different diseases | No | No | No |
| Form of statistical analysis to deal with factorial design (e.g. inside-the-table): including type of regression model if appropriate | For primary clinical outcome | Logistic regression | Linear regression/ANCOVA/ANOVA | Linear regression/ANCOVA/ANOVA |
|  |  | Not stated | Not stated | Interaction term included regardless of significance |
|  |  | Comment that "Due to the nested factorial nature of the study, we will use multi-level modelling to explore potential interactions between each of the levels nested within the trial (screening method and intervention)" | "The outcomes measured at 4, 8, 12, 26 and 52 weeks will be used to generate efficacy and recurrence indices that will be analysed using linear mixed and logistic regression models. […] Alpha will be set at 0.01 to compensate for the possible increase in type I error rates that may result from multiple testing." | Patients clustered within GPs within practices |
|  | Base case economic evaluation | Unclear | Not stated | Unclear |
|  |  | "The economic analysis will calculate the incremental cost-effectiveness of the control condition with the AHW condition under study" |  | Protocol not clear on methods for economic evaluation: Secondary outcomes will be compared using regression analysis. Regarding economic evaluation, simply state that "The statistical uncertainty regarding the cost-effectiveness of the different strategies will be assessed using non-parametric bootstrap resampling techniques and result in estimates of net monetary benefit values" |
|  | Sensitivity analyses of economic evaluation | Not stated | Not stated | Not stated |
| Presentation of economic results | | N/A | N/A | N/A |
| Presentation of uncertainty (e.g. pair-wise CEAC, EVPI) | | Plan to construct CEACs showing "the probability that the brief intervention is more cost-effective than usual care" | N/A | N/A |

| **Author/study acronym** | | **MOA [106]** | **OPTIMA [107]** | **OPTIMAL [108]** |
| --- | --- | --- | --- | --- |
| Year of publication for earliest paper presenting ***methods*** of economic evaluation | | 2009 | 2003 | 2009 |
| Protocol or completed study? | | Protocol | Protocol | Protocol |
| Anticipated publication date of ec anal (for protocols only) | | 1-year data anticipated in June 2010; 2-year data in 2011 | Clinical results were published in 2011 |  |
| Type of factorial design (e.g. partial/fractional) | | Full factorial | Full factorial | Full factorial |
| Projected total sample size (across all arms) | | 224 | 1700 planned | 340 |
| Size of factorial matrix (e.g. 2x2) | | 2x2 | 2x2 | 2x2 |
| Disease area | | Osteoarthritis | HIV | Prolapse and stress incontinence symptoms |
| Sponsor | | Other public funding body | Other UK public funding body | US public funding body |
| Country | | New Zealand | US, Canada and UK | US |
| Treatments (or treatment combinations) compared | | Factor 1: multi-modal exercise vs. no exercise. Factor 2: individualised manual therapy vs. no manual therapy | Factor 1: mega-ART (antiretroviral therapy) vs. standard ART. Factor 2: 3-month antiretroviral drug-free period (ARDFP). | Factor 1: sacrospinous ligament fixation vs. uterosacral vaginal vault suspension (two different surgical interventions). Factor 2: perioperative behavioural and pelvic floor muscle training vs. usual perioperative care |
| Type of comparisons | | 0 vs. a vs. b vs. ab | 0 vs. a vs. b vs. ab | A0 vs. AX vs. B0 vs. BX (Factor 1: interventions A and B; Factor 2: X vs. 0) |
| Type(s) of treatment evaluated | | Physiotherapy and related techniques | Pharmaceutical | Surgery and physiotherapy |
| Power calculation allowed for interaction effect and/or powered to test for interaction? | | 46% power to detect interaction; no interaction is expected | Unclear. Power calculation section in Kyriakides 2003 mentions only mega vs. standard, not ARDFP | Power calculation assumes no interaction and was based on at-the-margins analysis |
| Type of economic evaluation (e.g. CUA) | | CUA | CUA | CUA |
| Other notable aspects of study design | Cluster randomised | No | No | No |
|  | Cross-over | No | No | No |
|  | Intentionally unbalanced | No | No | No |
|  | Interventions target different diseases | No | No | Yes |
| Form of statistical analysis to deal with factorial design (e.g. inside-the-table): including type of regression model if appropriate | For primary clinical outcome | Linear regression/ANCOVA/ANOVA | At-the-margins | Linear regression/ANCOVA/ANOVA |
|  |  | Interactions will be ignored unless statistically significant |  | Not stated |
|  |  | Secondary analyses will assess interaction by including interaction terms in the models. The coefficients for interaction effects and their 95% CI will be reported. | "Primary comparisons will be the main effects: standard ART vs. mega-ART and ARDFP vs. no ARDFP". "If the primary analyses of main effects show a significant difference between the strategies, secondary analyses will compare the pair-wise treatment strategy comparisons [adjusting for multiple tests]." "Qualitative interactions between the main effects (ART and ARDFP) are not anticipated. If, however, such interactions are found, then no main effects will be reported. Quantitative interactions, which are more likely to be present, will be assessed prior to the analysis of the main effects; these main effects will nonetheless be reported." | Logistic regression will be used for surgical success and GLMs will be used for outcomes for periopreative interventions. Both models will include both treatment assignments. Although it is not explicitly stated whether interactions will be taken into account, the protocol states that they will test for interactions. |
|  | Base case economic evaluation | Not stated | Unclear | Linear regression/ANCOVA/ANOVA |
|  |  | Only very brief details of economic evaluation given in protocol | Only brief details given | "The incremental cost-effectiveness ratio (ICER) will be calculated as the differential costs between the perioperative BPMT group and the usual care group, divided by the differential QALYs between the two groups. General linear regression analysis will be conducted to estimate the differential mean costs and differential mean QALYs between the BPMT group and the usual care group, while adjusting for surgical treatment (SSLF or ULS), hysterectomy (concomitant or prior), surgeon, and other baseline measures identified as unbalanced between the two groups. The measures of costs and QALYs may be transformed before analysis if the variables demonstrate skewed distributions." |
|  | Sensitivity analyses of economic evaluation | Not stated |  | Not stated |
| Presentation of economic results | | Not stated |  | Cost-effectiveness of perioperative behavioural intervention vs. usual care will be assessed based on regression analysis. Cost-effectiveness of surgical interventions does not appear to be assessed |
| Presentation of uncertainty (e.g. pair-wise CEAC, EVPI) | | Not stated | Protocol indicates that authors plan to use bootstrapping and calculate CEACs. Only brief details given, although the comment that CEACs plot "the probability of one strategy being more cost-effective than the other two" suggests that the authors plan to generate CEACs for multiple comparisons, but ignore one of the 4 arms. | Not stated. 95% CI around ICER will be based on bootstrapping |

| **Author/study acronym** | | **PHC study [109]** | **PORTSSS [110]** | **SIGNET [111]** |
| --- | --- | --- | --- | --- |
| Year of publication for earliest paper presenting ***methods*** of economic evaluation | | 2009 | 2009 | 2007 |
| Protocol or completed study? | | Protocol | Protocol | Protocol |
| Anticipated publication date of ec anal (for protocols only) | | 30 month trial began April 2008 | Not stated | Summer 2009 |
| Type of factorial design (e.g. partial/fractional) | | Full factorial | Full factorial | Full factorial |
| Projected total sample size (across all arms) | | 744 | 2576 | 500 |
| Size of factorial matrix (e.g. 2x2) | | 3x2x2 | 2x2 | 2x2 |
| Disease area | | Alcohol abuse | Smoking cessation | Parenteral nutrition |
| Sponsor | | Department of Health | Department of Health | MRC |
| Country | | UK | UK | UK |
| Treatments (or treatment combinations) compared | | Factor 1: intensity of brief intervention (patient information leaflet [PIL]; brief advice [BA]; and brief lifestyle counselling [BLC]). Factor 2: screening approach (targeted vs. universal screening). Factor 3: screening method (FAST or M-SASQ questionnaires) | Factor 1: Proactive vs. reactive support. Factor 2: the offer of NRT vs. no offer. | Factor 1: glutamine vs. no additional glutamine. Factor 2: selenium vs. no additional selenium |
| Type of comparisons | | A1 vs. A2 vs. B1 vs. B2 (≥2 dosing regimens of ≥2 interventions) | 0 vs. a vs. b vs. ab | 0 vs. a vs. b vs. ab |
| Type(s) of treatment evaluated | | Other | Pharmaceutical + other | Nutritional supplement |
| Power calculation allowed for interaction effect and/or powered to test for interaction? | | Not stated. Comments that "The sample size calculation was designed to account primarily for intervention level outcomes. Powering the study in this way will also account statistically for appropriate outcomes for screening approach and screening method." | No - assumes no interactions based on previous evidence | Not stated - protocol comments that "evidence will be sought for an interaction between glutamine and selenium" but power calculation appears to be based on at-the-margins approach with no interaction. |
| Type of economic evaluation (e.g. CUA) | | CEA and CUA | CEA and CUA | CUA |
| Other notable aspects of study design | Cluster randomised | Yes | No | No |
|  | Cross-over | No | No | No |
|  | Intentionally unbalanced | No | No | No |
|  | Interventions target different diseases | No | No | No |
| Form of statistical analysis to deal with factorial design (e.g. inside-the-table): including type of regression model if appropriate | For primary clinical outcome | Logistic regression | At-the-margins | At-the-margins |
|  |  | Not stated | Interactions will be ignored unless statistically significant | Not stated |
|  |  | Comment that "we will use multi-level modelling to explore potential interactions between each of the three levels nested within the trial (screening approach, screening method and intervention)" |  | The first two "principal comparisons" comprised at-the-margins comparisons, while the third comprised "all randomised glutamine and selenium vs. all randomised glutamine alone vs. all randomised selenium alone vs. all randomised placebo". Authors stated that "Dichotomous endpoints will be analysed using statistical tests such as the Chi-squared test" |
|  | Base case economic evaluation | Unclear | Not stated | Not stated |
|  |  | "The economic analysis will calculate the incremental cost-effectiveness of the control condition with each of the screening and brief intervention conditions under study" | In addition to within trial analysis (cost/additional quitter), plan to use model to calculate lifetime cost/QALY | Outline of economic evaluation in the protocol simply says: “The type of economic evaluation performed will depend upon the findings |
|  | Sensitivity analyses of economic evaluation | Not stated | Not stated | Not stated |
| Presentation of economic results | | N/A | N/A | N/A |
| Presentation of uncertainty (e.g. pair-wise CEAC, EVPI) | | Plan to construct CEACs showing "the probability that the brief intervention is more cost-effective than usual care" | State that plan to conduct PSA, but give no other details | N/A |

| **Author/study acronym** | | **STOOL [112]** | **ZAMSTAR [113]** |
| --- | --- | --- | --- |
| Year of publication for earliest paper presenting ***methods*** of economic evaluation | | 2008 | 2008 |
| Protocol or completed study? | | Terminated early, so no results; treated as protocol | Protocol |
| Anticipated publication date of ec anal (for protocols only) | | N/A | Not stated |
| Type of factorial design (e.g. partial/fractional) | | Incomplete factorial | Full factorial |
| Projected total sample size (across all arms) | | 1002 planned; 19 recruited; 7 completed trial taking intervention as allocated | 24 communities covering approx 120,000 individuals |
| Size of factorial matrix (e.g. 2x2) | | 3x3 | 2x2 |
| Disease area | | Constipation | Tuberculosis and HIV |
| Sponsor | | NIHR HTA | Other public funding body |
| Country | | UK | Zambia and South Africa |
| Treatments (or treatment combinations) compared | | Factor 1: bulk laxative, stimulant laxative or osmotic laxative. Factor 2: adding in either a bulk laxative, stimulant laxative or osmotic laxative. Cells of the design where patients would have added in the same type of drug as used first-line were omitted from the design. | Factor 1: enhanced case finding intervention. Factor 2: household intervention |
| Type of comparisons | | Other | 0 vs. a vs. b vs. ab |
| Type(s) of treatment evaluated | | Pharmaceutical | Other |
| Power calculation allowed for interaction effect and/or powered to test for interaction? | | Not stated | No: Powered based on no interaction on multiplicative scale and not powered to detect interactions |
| Type of economic evaluation (e.g. CUA) | | Planned to conduct CMA if no "statistically significant evidence that one strategy is more effective than another", and otherwise CCA and CEA; CUA was also mentioned in Appendix 5 | Not stated |
| Other notable aspects of study design | Cluster randomised | No | yes |
|  | Cross-over | No | No |
|  | Intentionally unbalanced | No | No |
|  | Interventions target different diseases | No | No |
|  | Other |  | Both interventions primarily target TB but also increase awareness of HIV |
| Form of statistical analysis to deal with factorial design (e.g. inside-the-table): including type of regression model if appropriate | For primary clinical outcome | Poisson regression | Linear regression/ANCOVA/ANOVA |
|  |  | No mention of interaction term | Not stated |
|  |  |  | GLMs on log-incidence that allow for clustering. No clear indication of whether interaction terms will be included |
|  | Base case economic evaluation | Not stated | Not stated |
|  |  |  | Not stated: methods indicate that costs will be evaluated using linear regression and that cost-effectiveness will be assessed, but methods not stated |
|  | Sensitivity analyses of economic evaluation | None | Not stated |
| Presentation of economic results | | N/A | N/A |
| Presentation of uncertainty (e.g. pair-wise CEAC, EVPI) | | N/A | Not stated |

**References**

1. Pinto DS, Stone GW, Shi C, Dunn ES, Reynolds MR, York M, Walczak J, Berezin RH, Mehran R, McLaurin BT *et al*. Economic Evaluation of Bivalirudin With or Without Glycoprotein IIb/IIIa Inhibition Versus Heparin With Routine Glycoprotein IIb/IIIa Inhibition for Early Invasive Management of Acute Coronary Syndromes. Journal of the American College of Cardiology 2008, 52(22):1758-1768.

2. Stone GW, Bertrand M, Colombo A, Dangas G, Farkouh ME, Feit F, Lansky AJ, Lincoff AM, Mehran R, Moses JW *et al*. Acute Catheterization and Urgent Intervention Triage strategY (ACUITY) trial: study design and rationale. Am Heart J 2004, 148(5):764-775. https://doi.org/10.1016/j.ahj.2004.04.036.

3. Stone GW, White HD, Ohman EM, Bertrand ME, Lincoff AM, McLaurin BT, Cox DA, Pocock SJ, Ware JH, Feit F *et al*. Bivalirudin in patients with acute coronary syndromes undergoing percutaneous coronary intervention: a subgroup analysis from the Acute Catheterization and Urgent Intervention Triage strategy (ACUITY) trial. Lancet 2007, 369(9565):907-919. [https://doi.org/](https://doi.org/S0140-6736(07)60450-4)10.1016/S0140-6736(07)60450-4.

4. Stone GW, McLaurin BT, Cox DA, Bertrand ME, Lincoff AM, Moses JW, White HD, Pocock SJ, Ware JH, Feit F *et al*. Bivalirudin for patients with acute coronary syndromes. The New England journal of medicine 2006, 355(21):2203-2216. https://doi.org/10.1056/NEJMoa062437.

5. Sevick MA, Miller GD, Loeser RF, Williamson JD, Messier SP. Cost-effectiveness of exercise and diet in overweight and obese adults with knee osteoarthritis. Med Sci Sports Exerc 2009, 41(6):1167-1174. <https://doi.org/10.1249/MSS.0b013e318197ece7>.

6. Messier SP, Loeser RF, Miller GD, Morgan TM, Rejeski WJ, Sevick MA, Ettinger WH, Jr., Pahor M, Williamson JD. Exercise and dietary weight loss in overweight and obese older adults with knee osteoarthritis: the Arthritis, Diet, and Activity Promotion Trial. Arthritis Rheum 2004, 50(5):1501-1510. <https://doi.org/10.1002/art.20256>.

7. Miller GD, Rejeski WJ, Williamson JD, Morgan T, Sevick MA, Loeser RF, Ettinger WH, Messier SP. The Arthritis, Diet and Activity Promotion Trial (ADAPT): design, rationale, and baseline results. Control Clin Trials 2003, 24(4):462-480. <https://doi.org/S0197245603000631>.

8. Reddy P, Kalus JS, Caron MF, Horowitz S, Karapanos A, Coleman CI, Kluger J, White CM. Economic analysis of intravenous plus oral amiodarone, atrial septal pacing, and both strategies to prevent atrial fibrillation after open heart surgery. Pharmacotherapy 2004, 24(8):1013-1019.

9. White CM, Caron MF, Kalus JS, Rose H, Song J, Reddy P, Gallagher R, Kluger J. Intravenous plus oral amiodarone, atrial septal pacing, or both strategies to prevent post-cardiothoracic surgery atrial fibrillation: the Atrial Fibrillation Suppression Trial II (AFIST II). Circulation 2003, 108 Suppl 1:II200-206. <https://doi.org/10.1161/01.cir.0000087445.59819.6f>.

10. Lindgren P, Buxton M, Kahan T, Poulter NR, Dahlof B, Sever PS, Wedel H, Jonsson B. Cost-effectiveness of atorvastatin for the prevention of coronary and stroke events: an economic analysis of the Anglo-Scandinavian Cardiac Outcomes Trial--lipid-lowering arm (ASCOT-LLA). Eur J Cardiovasc Prev Rehabil 2005, 12(1):29-36. <https://doi.org/00149831-200502000-00005>.

11. Lindgren P, Buxton M, Kahan T, Poulter NR, Dahlof B, Sever PS, Wedel H, Jonsson B. Economic evaluation of ASCOT-BPLA: antihypertensive treatment with an amlodipine-based regimen is cost effective compared with an atenolol-based regimen. Heart 2008, 94(2):e4. https://doi.org/10.1136/hrt.2007.127217.

12. Lindgren P, Buxton M, Kahan T, Poulter NR, Dahlof B, Sever PS, Wedel H, Jonsson B. The lifetime cost effectiveness of amlodipine-based therapy plus atorvastatin compared with atenolol plus atorvastatin, amlodipine-based therapy alone and atenolol-based therapy alone: results from ASCOT1. Pharmacoeconomics 2009, 27(3):221-230.

13. Sever PS, Dahlof B, Poulter NR, Wedel H, Beevers G, Caulfield M, Collins R, Kjeldsen SE, McInnes GT, Mehlsen J *et al*. Rationale, design, methods and baseline demography of participants of the Anglo-Scandinavian Cardiac Outcomes Trial. ASCOT investigators. J Hypertens 2001, 19(6):1139-1147.

14. Dahlof B, Sever PS, Poulter NR, Wedel H, Beevers DG, Caulfield M, Collins R, Kjeldsen SE, Kristinsson A, McInnes GT *et al*. Prevention of cardiovascular events with an antihypertensive regimen of amlodipine adding perindopril as required versus atenolol adding bendroflumethiazide as required, in the Anglo-Scandinavian Cardiac Outcomes Trial-Blood Pressure Lowering Arm (ASCOT-BPLA): a multicentre randomised controlled trial. Lancet 2005, 366(9489):895-906. https://doi.org/10.1016/S0140-6736(05)67185-1.

15. Sever PS, Dahlof B, Poulter NR, Wedel H, Beevers G, Caulfield M, Collins R, Kjeldsen SE, Kristinsson A, McInnes GT *et al*. Prevention of coronary and stroke events with atorvastatin in hypertensive patients who have average or lower-than-average cholesterol concentrations, in the Anglo-Scandinavian Cardiac Outcomes Trial--Lipid Lowering Arm (ASCOT-LLA): a multicentre randomised controlled trial. Lancet 2003, 361(9364):1149-1158. [https://doi.org/](https://doi.org/S0140-6736(03)12948-0)10.1016/S0140-6736(03)12948-0.

16. Sever P, Dahlof B, Poulter N, Wedel H, Beevers G, Caulfield M, Collins R, Kjeldsen S, Kristinsson A, McInnes G *et al*. Potential synergy between lipid-lowering and blood-pressure-lowering in the Anglo-Scandinavian Cardiac Outcomes Trial. Eur Heart J 2006, 27(24):2982-2988. https://doi.org/10.1093/eurheartj/ehl403.

17. Bobadilla JF, Merikle E, Garcia M, Darba J, Sanchez C. Cost-effectiveness of atorvastatin plus amlodipine versus atorvastatin plus atenolol in hypertensive patients without previous coronary heart disease, normal to mildly elevated cholesterol levels and at least 3 cardiovascular risk factors [Abstract PCV34]. Value in Health 2007, 10(6):A415-A416.

18. Hollinghurst S, Sharp D, Ballard K, Barnett J, Beattie A, Evans M, Lewith G, Middleton K, Oxford F, Webley F *et al*. Randomised controlled trial of Alexander technique lessons, exercise, and massage (ATEAM) for chronic and recurrent back pain: economic evaluation. BMJ 2008, 337:a2656.

19. Little P, Lewith G, Webley F, Evans M, Beattie A, Middleton K, Barnett J, Ballard K, Oxford F, Smith P *et al*. Randomised controlled trial of Alexander technique lessons, exercise, and massage (ATEAM) for chronic and recurrent back pain. Bmj 2008, 337:a884.

20. Bankhead C, Richards SH, Peters TJ, Sharp DJ, Hobbs FD, Brown J, Roberts L, Tydeman C, Redman V, Formby J *et al*. Improving attendance for breast screening among recent non-attenders: a randomised controlled trial of two interventions in primary care. J Med Screen 2001, 8(2):99-105.

21. Brown J, Welton NJ, Bankhead C, Richards SH, Roberts L, Tydeman C, Peters TJ. A Bayesian approach to analysing the cost-effectiveness of two primary care interventions aimed at improving attendance for breast screening. Health Econ 2006, 15(5):435-445. <https://doi.org/10.1002/hec.1077>.

22. Hlatky MA, Boothroyd DB, Melsop KA, Kennedy L, Rihal C, Rogers WJ, Venkitachalam L, Brooks MM. Economic outcomes of treatment strategies for type 2 diabetes mellitus and coronary artery disease in the Bypass Angioplasty Revascularization Investigation 2 Diabetes trial. Circulation 2009, 120(25):2550-2558. <https://doi.org/CIRCULATIONAHA.109.912709>.

23. Frye RL, August P, Brooks MM, Hardison RM, Kelsey SF, MacGregor JM, Orchard TJ, Chaitman BR, Genuth SM, Goldberg SH *et al*. A randomized trial of therapies for type 2 diabetes and coronary artery disease. The New England journal of medicine 2009, 360(24):2503-2515. <https://doi.org/NEJMoa0805796>.

24. Brooks MM, Frye RL, Genuth S, Detre KM, Nesto R, Sobel BE, Kelsey SF, Orchard TJ. Hypotheses, Design, and Methods for the Bypass Angioplasty Revascularization Investigation 2 Diabetes (BARI 2D) Trial. The American Journal of Cardiology 2006, 97(12, Supplement 1):9-19.

25. Barnett PG, Masson CL, Sorensen JL, Wong W, Hall S. Linking opioid-dependent hospital patients to drug treatment: health care use and costs 6 months after randomization. Addiction 2006, 101(12):1797-1804.

26. Sorensen JL, Masson CL, Delucchi K, Sporer K, Barnett PG, Mitsuishi F, Lin C, Song Y, Chen T, Hall SM. Randomized trial of drug abuse treatment-linkage strategies. J Consult Clin Psychol 2005, 73(6):1026-1035. https://doi.org/10.1037/0022-006X.73.6.1026.

27. Sullivan FM, Swan IR, Donnan PT, Morrison JM, Smith BH, McKinstry B, Davenport RJ, Vale LD, Clarkson JE, Hammersley V *et al*. Early treatment with prednisolone or acyclovir in Bell's palsy. The New England journal of medicine 2007, 357(16):1598-1607. [https://doi.org/](https://doi.org/357/16/1598)10.1056/NEJMoa072006.

28. Sullivan FM, Swan IR, Donnan PT, Morrison JM, Smith BH, McKinstry B, Davenport RJ, Vale LD, Clarkson JE, Hernandez R *et al*. A randomised controlled trial of the use of aciclovir and/or prednisolone for the early treatment of Bell's palsy: the BELLS study. Health Technol Assess 2009, 13(47):iii-iv, ix-xi 1-130. <https://doi.org/10.3310/hta13470>.

29. Hernandez RA, Sullivan F, Donnan P, Swan I, Vale L, for the Bells Trial Group. Economic evaluation of early administration of prednisolone and/or aciclovir for the treatment of Bell's palsy. Family Practice 2009, 26(2):137-144.

30. Boyle J, McCartney E, Forbes J, O'Hare A. A randomised controlled trial and economic evaluation of direct versus indirect and individual versus group modes of speech and language therapy for children with primary language impairment. Health Technol Assess 2007, 11(25):iii-iv, xi-xii, 1-139. <https://doi.org/99/36/04>.

31. Dickson K, Marshall M, Boyle J, McCartney E, O'Hare A, Forbes J. Cost analysis of direct versus indirect and individual versus group modes of manual-based speech-and-language therapy for primary school-age children with primary language impairment. Int J Lang Commun Disord 2009, 44(3):369-381. https://doi.org/10.1080/13682820802137041.

32. Brandon TH, Collins BN, Juliano LM, Lazev AB. Preventing relapse among former smokers: a comparison of minimal interventions through telephone and mail. J Consult Clin Psychol 2000, 68(1):103-113.

33. Brandon TH, Meade CD, Herzog TA, Chirikos TN, Webb MS, Cantor AB. Efficacy and cost-effectiveness of a minimal intervention to prevent smoking relapse: dismantling the effects of amount of content versus contact. J Consult Clin Psychol 2004, 72(5):797-808. [https://doi.org/](https://doi.org/2004-19094-007)10.1037/0022-006X.72.5.797.

34. Bakhai A, Stone GW, Grines CL, Murphy SA, Githiora L, Berezin RH, Cox DA, Stuckey T, Griffin JJ, Tcheng JE *et al*. Cost-effectiveness of coronary stenting and abciximab for patients with acute myocardial infarction: results from the CADILLAC (Controlled Abciximab and Device Investigation to Lower Late Angioplasty Complications) trial. Circulation 2003, 108(23):2857-2863. <https://doi.org/10.1161/01.CIR.0000103121.26241.FA>.

35. Stone GW, Grines CL, Cox DA, Garcia E, Tcheng JE, Griffin JJ, Guagliumi G, Stuckey T, Turco M, Carroll JD *et al*. Comparison of angioplasty with stenting, with or without abciximab, in acute myocardial infarction. The New England journal of medicine 2002, 346(13):957-966. <https://doi.org/10.1056/NEJMoa013404>.

36. Cantor JC, Morisky DE, Green LW, Levine DM, Salkever DS. Cost-effectiveness of educational interventions to improve patient outcomes in blood pressure control. Prev Med 1985, 14(6):782-800.

37. Morisky DE, Levine DM, Green LW, Shapiro S, Russell RP, Smith CR. Five-year blood pressure control and mortality following health education for hypertensive patients. Am J Public Health 1983, 73(2):153-162.

38. Green LW, Levine DM, Deeds S. Clinical trials of health education for hypertensive outpatients: design and baseline data. Prev Med 1975, 4(4):417-425.

39. Johannesson M, Borgquist L, Jonsson B, Lindholm LH. The cost effectiveness of lipid lowering in Swedish primary health care. The CELL Study Group. J Intern Med 1996, 240(1):23-29.

40. Lindholm LH, Ekbom T, Dash C, Isacsson AKE, Schersten B. Changes in cardiovascular risk factors by combined pharmacological and nonpharmacological strategies: the main results of the CELL Study. Journal of Internal Medicine 1996, 240(1):13-22.

41. Effing T, Kerstjens H, van der Valk P, Zielhuis G, van der Palen J. (Cost)-effectiveness of self-treatment of exacerbations on the severity of exacerbations in patients with COPD: the COPE II study. Thorax 2009, 64(11):956-962.

42. Clarkson JE, Turner S, Grimshaw JM, Ramsay CR, Johnston M, Scott A, Bonetti D, Tilley CJ, Maclennan G, Ibbetson R *et al*. Changing clinicians' behavior: a randomized controlled trial of fees and education. J Dent Res 2008, 87(7):640-644. <https://doi.org/87/7/640>.

43. Tworoger SS, Yasui Y, Ulrich CM, Nakamura H, LaCroix K, Johnston R, McTiernan A. Mailing strategies and recruitment into an intervention trial of the exercise effect on breast cancer biomarkers. Cancer Epidemiol Biomarkers Prev 2002, 11(1):73-77.

44. Janzon M, Levin LA, Swahn E. Cost-effectiveness of an invasive strategy in unstable coronary artery disease; results from the FRISC II invasive trial. The Fast Revascularisation during InStability in Coronary artery disease. Eur Heart J 2002, 23(1):31-40. <https://doi.org/10.1053/euhj.2001.2695>.

45. Janzon M, Levin LA, Swahn E. Cost effectiveness of extended treatment with low molecular weight heparin (dalteparin) in unstable coronary artery disease: results from the FRISC II trial. Heart 2003, 89(3):287-292.

46. Wallentin L, Lagerqvist B, Husted S, Kontny F, Stahle E, Swahn E. Outcome at 1 year after an invasive compared with a non-invasive strategy in unstable coronary-artery disease: the FRISC II invasive randomised trial. FRISC II Investigators. Fast Revascularisation during Instability in Coronary artery disease. Lancet 2000, 356(9223):9-16.

47. Moller BH, Janzon M, Levin LA, Swahn E. Comment concerning 'cost-effectiveness of an invasive strategy in unstable coronary artery disease' [2] (multiple letters). European Heart Journal 2002, 23(20):1634-1635.

48. Invasive compared with non-invasive treatment in unstable coronary-artery disease: FRISC II prospective randomised multicentre study. FRagmin and Fast Revascularisation during InStability in Coronary artery disease Investigators. Lancet 1999, 354(9180):708-715. <https://doi.org/S0140673699073493>.

49. Long-term low-molecular-mass heparin in unstable coronary-artery disease: FRISC II prospective randomised multicentre study. FRagmin and Fast Revascularisation during InStability in Coronary artery disease. Investigators. Lancet 1999, 354(9180):701-707. <https://doi.org/S014067369907350X>.

50. Franzosi MG, Maggioni AP, Santoro E, Tognoni G, Cavalieri E. Cost-effectiveness analysis of early lisinopril use in patients with acute myocardial infarction. Results from GISSI-3 trial. Pharmacoeconomics 1998, 13(3):337-346.

51. GISSI-3: effects of lisinopril and transdermal glyceryl trinitrate singly and together on 6-week mortality and ventricular function after acute myocardial infarction. Gruppo Italiano per lo Studio della Sopravvivenza nell'infarto Miocardico. Lancet 1994, 343(8906):1115-1122.

52. Franzosi MG, Brunetti M, Marchioli R, Marfisi RM, Tognoni G, Valagussa F. Cost-effectiveness analysis of n-3 polyunsaturated fatty acids (PUFA) after myocardial infarction: results from Gruppo Italiano per lo Studio della Sopravvivenza nell'Infarto (GISSI)-Prevenzione Trial. Pharmacoeconomics 2001, 19(4):411-420.

53. Dietary supplementation with n-3 polyunsaturated fatty acids and vitamin E after myocardial infarction: results of the GISSI-Prevenzione trial. Gruppo Italiano per lo Studio della Sopravvivenza nell'Infarto miocardico. Lancet 1999, 354(9177):447-455. <https://doi.org/S0140673699070725>.

54. Halpern SD, Ubel PA, Berlin JA, Asch DA. Randomized Trial of $5 Versus $10 Monetary Incentives, Envelope Size, and Candy to Increase Physician Response Rates to Mailed Questionnaires. Medical Care 2002, 40(9):834-839.

55. Hollis JF, McAfee TA, Fellows JL, Zbikowski SM, Stark M, Riedlinger K. The effectiveness and cost effectiveness of telephone counselling and the nicotine patch in a state tobacco quitline. Tobacco Control 2007, 16 Supplement(1):i53-i59.

56. Heart Protection Study Collaborative Group. Statin Cost-Effectiveness in the United States for People at Different Vascular Risk Levels. Circulation: Cardiovascular Quality & Outcomes 2009, 2(2):65-72.

57. Mihaylova B, Briggs A, Armitage J, Parish S, Gray A, Collins R. Lifetime cost effectiveness of simvastatin in a range of risk groups and age groups derived from a randomised trial of 20,536 people. BMJ 2006, 333(7579):1145. <https://doi.org/bmj.38993.731725.BE>.

58. Mihaylova B, Briggs A, Armitage J, Parish S, Gray A, Collins R. Cost-effectiveness of simvastatin in people at different levels of vascular disease risk: economic analysis of a randomised trial in 20,536 individuals. Lancet 2005, 365(9473):1779-1785. https://doi.org/10.1016/S0140-6736(05)63014-0.

59. MRC/BHF Heart Protection Study of cholesterol-lowering therapy and of antioxidant vitamin supplementation in a wide range of patients at increased risk of coronary heart disease death: early safety and efficacy experience. Eur Heart J 1999, 20(10):725-741. <https://doi.org/S0195668X98913501>.

60. MRC/BHF Heart Protection Study of antioxidant vitamin supplementation in 20,536 high-risk individuals: a randomised placebo-controlled trial. Lancet 2002, 360(9326):23-33. [https://doi.org/](https://doi.org/S0140-6736(02)09328-5)10.1016/S0140-6736(02)09328-5.

61. MRC/BHF Heart Protection Study of cholesterol lowering with simvastatin in 20,536 high-risk individuals: a randomised placebo-controlled trial. Lancet 2002, 360(9326):7-22. https://doi.org/10.1016/S0140-6736(02)09327-3.

62. Campbell MK, Carr C, Devellis B, Switzer B, Biddle A, Amamoo MA, Walsh J, Zhou B, Sandler R, Campbell MK *et al*. A randomized trial of tailoring and motivational interviewing to promote fruit and vegetable consumption for cancer prevention and control. Annals of Behavioral Medicine 2009, 38(2):71-85.

63. Foster EM, Jensen PS, Schlander M, Pelham WE, Jr., Hechtman L, Arnold LE, Swanson JM, Wigal T. Treatment for ADHD: is more complex treatment cost-effective for more complex cases? Health Serv Res 2007, 42(1 Pt 1):165-182. <https://doi.org/HESR599>.

64. Jensen PS, Garcia JA, Glied S, Crowe M, Foster M, Schlander M, Hinshaw S, Vitiello B, Arnold LE, Elliott G *et al*. Cost-effectiveness of ADHD treatments: findings from the multimodal treatment study of children with ADHD. Am J Psychiatry 2005, 162(9):1628-1636. https://doi.org/10.1176/appi.ajp.162.9.1628.

65. Wells KC, Pelham WE, Kotkin RA, Hoza B, Abikoff HB, Abramowitz A, Arnold LE, Cantwell DP, Conners CK, Del Carmen R *et al*. Psychosocial treatment strategies in the MTA study: rationale, methods, and critical issues in design and implementation. J Abnorm Child Psychol 2000, 28(6):483-505.

66. A 14-month randomized clinical trial of treatment strategies for attention-deficit/hyperactivity disorder. The MTA Cooperative Group. Multimodal Treatment Study of Children with ADHD. Arch Gen Psychiatry 1999, 56(12):1073-1086.

67. Atthobari J, Asselbergs FW, Boersma C, de Vries R, Hillege HL, van Gilst WH, Gansevoort RT, de Jong PE, de Jong-van den Berg LT, Postma MJ. Cost-effectiveness of screening for albuminuria with subsequent fosinopril treatment to prevent cardiovascular events: A pharmacoeconomic analysis linked to the prevention of renal and vascular endstage disease (PREVEND) study and the prevention of renal and vascular endstage disease intervention trial (PREVEND IT). Clin Ther 2006, 28(3):432-444. [https://doi.org/](https://doi.org/S0149-2918(06)00077-4)10.1016/j.clinthera.2006.03.012.

68. Asselbergs FW, Diercks GF, Hillege HL, van Boven AJ, Janssen WM, Voors AA, de Zeeuw D, de Jong PE, van Veldhuisen DJ, van Gilst WH. Effects of fosinopril and pravastatin on cardiovascular events in subjects with microalbuminuria. Circulation 2004, 110(18):2809-2816. [https://doi.org/](https://doi.org/01.CIR.0000146378.65439.7A)10.1161/01.CIR.0000146378.65439.7A.

69. Richards SH, Bankhead C, Peters TJ, Austoker J, Hobbs FD, Brown J, Tydeman C, Roberts L, Formby J, Redman V *et al*. Cluster randomised controlled trial comparing the effectiveness and cost-effectiveness of two primary care interventions aimed at improving attendance for breast screening. J Med Screen 2001, 8(2):91-98.

70. Welton NJ, Ades AE, Caldwell DM, Peters TJ. Research prioritization based on expected value of partial perfect information: a case-study on interventions to increase uptake of breast cancer screening. J R Statist Soc A 2008, 171(Part 4):807-841.

71. Rodewald LE, Szilagyi PG, Humiston SG, Barth R, Kraus R, Raubertas RF. A randomized study of tracking with outreach and provider prompting to improve immunization coverage and primary care. Pediatrics 1999, 103(1):31-38.

72. Saillour-Glenisson F, Michel P, Daucourt V. [Medico-economic assessment of two methods for implementing thyroid testing guidelines]. Rev Epidemiol Sante Publique 2005, 53 Spec No 1:1S79-88. <https://doi.org/MDOI-RESP-09-2005-53-HS1-0398-7620-101019-200505046>.

73. Salize HJP, Merkel SD-R, Reinhard ID-M, Twardella DP, Mann KMD, Brenner HMDMPH. Cost-effective Primary Care-Based Strategies to Improve Smoking Cessation: More Value for Money. Archives of Internal Medicine 2009, 169(3):230-235.

74. Twardella D, Brenner H. Effects of practitioner education, practitioner payment and reimbursement of patients' drug costs on smoking cessation in primary care: a cluster randomised trial. Tob Control 2007, 16(1):15-21.

75. McKenna C, Bojke L, Manca A, Adebajo A, Dickson J, Helliwell P, Morton V, Russell I, Torgerson D, Watson J. Shoulder acute pain in primary health care: is retraining GPs effective? The SAPPHIRE randomized trial: a cost-effectiveness analysis. Rheumatology (Oxford) 2009, 48(5):558-563. https://doi.org/10.1093/rheumatology/kep008.

76. Watson J, Helliwell P, Morton V, Adebajo A, Dickson J, Russell I, Torgerson D. Shoulder acute pain in primary healthcare: is retraining effective for GP principals? SAPPHIRE--a randomized controlled trial. Rheumatology (Oxford) 2008, 47(12):1795-1802. [https://doi.org/](https://doi.org/ken360)10.1093/rheumatology/ken360.

77. Thomas KSP, Miller PM, Doherty MM, Muir KRP, Jones ACM, O'Reilly SCM. Cost Effectiveness of a Two-Year Home Exercise Program for the Treatment of Knee Pain. Arthritis & Rheumatism 2005, 53(3) Arthritis Care &(Research):388-394.

78. Thomas KS, Muir KR, Doherty M, Jones AC, O'Reilly SC, Bassey EJ. Home based exercise programme for knee pain and knee osteoarthritis: randomised controlled trial. Bmj 2002, 325(7367):752.

79. Thompson RS, Michnich ME, Gray J, Friedlander L, Gilson B. Maximizing compliance with hemoccult screening for colon cancer in clinical practice. Med Care 1986, 24(10):904-914.

80. Desai AS, Solomon DH, Stone PH, Avorn J. Economic consequences of routine coronary angiography in low- and intermediate-risk patients with unstable angina pectoris. Am J Cardiol 2003, 92(4):363-367. <https://doi.org/S0002914903006507>.

81. Effects of tissue plasminogen activator and a comparison of early invasive and conservative strategies in unstable angina and non-Q-wave myocardial infarction. Results of the TIMI IIIB Trial. Thrombolysis in Myocardial Ischemia. Circulation 1994, 89(4):1545-1556.

82. Tjan-Heijnen VCG, Caleo S, Postmus PE, Ardizzoni A, Burghouts JTM, Buccholz E, Biesma B, Gorlia T, Crott R, Giaccone G *et al*. Economic evaluation of antibiotic prophylaxis in small-cell lung cancer patients receiving chemotherapy: an EORTC double-blind placebo-controlled phase III study (08923). Annals of Oncology 2003, 14(2):248-257.

83. Tjan-Heijnen VC, Postmus PE, Ardizzoni A, Manegold CH, Burghouts J, van Meerbeeck J, Gans S, Mollers M, Buchholz E, Biesma B *et al*. Reduction of chemotherapy-induced febrile leucopenia by prophylactic use of ciprofloxacin and roxithromycin in small-cell lung cancer patients: an EORTC double-blind placebo-controlled phase III study. Ann Oncol 2001, 12(10):1359-1368.

84. United Kingdom back pain exercise and manipulation (UK BEAM) randomised trial: cost effectiveness of physical treatments for back pain in primary care. BMJ 2004, 329(7479):1381. <https://doi.org/bmj.38282.607859.AE>.

85. United Kingdom back pain exercise and manipulation (UK BEAM) randomised trial: effectiveness of physical treatments for back pain in primary care. BMJ 2004, 329(7479):1377. https://doi.org/10.1136/bmj.38282.669225.AE.

86. UK Prospective Diabetes Study (UKPDS). VIII. Study design, progress and performance. Diabetologia 1991, 34(12):877-890.

87. Tight blood pressure control and risk of macrovascular and microvascular complications in type 2 diabetes: UKPDS 38. UK Prospective Diabetes Study Group. Bmj 1998, 317(7160):703-713.

88. Effect of intensive blood-glucose control with metformin on complications in overweight patients with type 2 diabetes (UKPDS 34). UK Prospective Diabetes Study (UKPDS) Group. Lancet 1998, 352(9131):854-865.

89. Intensive blood-glucose control with sulphonylureas or insulin compared with conventional treatment and risk of complications in patients with type 2 diabetes (UKPDS 33). UK Prospective Diabetes Study (UKPDS) Group. Lancet 1998, 352(9131):837-853.

90. Cost effectiveness analysis of improved blood pressure control in hypertensive patients with type 2 diabetes: UKPDS 40. UK Prospective Diabetes Study Group. Bmj 1998, 317(7160):720-726.

91. Gray A, Raikou M, McGuire A, Fenn P, Stevens R, Cull C, Stratton I, Adler A, Holman R, Turner R. Cost effectiveness of an intensive blood glucose control policy in patients with type 2 diabetes: economic analysis alongside randomised controlled trial (UKPDS 41). United Kingdom Prospective Diabetes Study Group. Bmj 2000, 320(7246):1373-1378.

92. Clarke P, Gray A, Adler A, Stevens R, Raikou M, Cull C, Stratton I, Holman R. Cost-effectiveness analysis of intensive blood-glucose control with metformin in overweight patients with type II diabetes (UKPDS No. 51). Diabetologia 2001, 44(3):298-304.

93. Gray A, Clarke P, Raikou M, Adler A, Stevens R, Neil A, Cull C, Stratton I, Holman R, the UG. An economic evaluation of atenolol vs. captopril in patients with Type 2 diabetes (UKPDS 54). Diabetic Medicine 2001, 18(6):438-444.

94. Gray AM, Clarke P. The economic analyses of the UK prospective diabetes study. Diabet Med 2008, 25 Suppl 2:47-51.

95. Clarke PM, Gray AM, Briggs A, Stevens RJ, Matthews DR, Holman RR, Study UUKPD. Cost-utility analyses of intensive blood glucose and tight blood pressure control in type 2 diabetes (UKPDS 72). Diabetologia 2005, 48(5):868-877. <https://doi.org/10.1007/s00125-005-1717-3>.

96. Campbell AJ, Robertson MC, La Grow SJ, Kerse NM, Sanderson GF, Jacobs RJ, Sharp DM, Hale LA. Randomised controlled trial of prevention of falls in people aged >=75 with severe visual impairment: the VIP trial. BMJ 2005, 331(7520):817.

97. Wesson J, Olawo A, Bukusi V, Solomon M, Pierre-Louis B, Stanback J, Janowitz B. Reaching providers is not enough to increase IUD use: a factorial experiment of 'academic detailing' in Kenya. J Biosoc Sci 2008, 40(1):69-82. [https://doi.org/](https://doi.org/S0021932007002027)10.1017/S0021932007002027.

98. Sullivan MD, Anderson RT, Aron D, Atkinson HH, Bastien A, Chen GJ, Feeney P, Gafni A, Hwang W, Katz LA *et al*. Health-Related Quality of Life and Cost-Effectiveness Components of the Action to Control Cardiovascular Risk in Diabetes (ACCORD) Trial: Rationale and Design. The American Journal of Cardiology 2007, 99(12, Supplement 1):S90-S102.

99. Buse JB, Bigger JT, Byington RP, Cooper LS, Cushman WC, Friedewald WT, Genuth S, Gerstein HC, Ginsberg HN, Goff DC, Jr. *et al*. Action to Control Cardiovascular Risk in Diabetes (ACCORD) trial: design and methods. Am J Cardiol 2007, 99(12A):21i-33i.

100. Coulton S, Perryman K, Bland M, Cassidy P, Crawford M, Deluca P, Drummond C, Gilvarry E, Godfrey C, Heather N *et al*. Screening and brief interventions for hazardous alcohol use in accident and emergency departments: A randomised controlled trial protocol. BMC Health Services Research 2009, 9(114)

101. Walker DG, Aedo C, Albala C, Allen E, Dangour AD, Elbourne D, Grundy E, Uauy R. Methods for economic evaluation of a factorial-design cluster randomised controlled trial of a nutrition supplement and an exercise programme among healthy older people living in Santiago, chile: The CENEX study. BMC Health Services Research 2009, 9(85)

102. Dangour AD, Albala C, Aedo C, Elbourne D, Grundy E, Walker D, Uauy R. A factorial-design cluster randomised controlled trial investigating the cost-effectiveness of a nutrition supplement and an exercise programme on pneumonia incidence, walking capacity and body mass index in older people living in Santiago, Chile: The CENEX study protocol. Nutrition Journal 2007, 6(14)

103. Newbury-Birch D, Bland M, Cassidy P, Coulton S, Deluca P, Drummond C, Gilvarry E, Godfrey C, Heather N, Kaner E *et al*. Screening and brief interventions for hazardous and harmful alcohol use in probation services: a cluster randomised controlled trial protocol. BMC Public Health 2009, 9:418.

104. Coombes BK, Bisset L, Connelly L, Brooks P, Vicenzino B. Optimising corticosteroid injection for lateral epicondylalgia with the addition of physiotherapy: A protocol for a randomised control trial with placebo comparison. BMC Musculoskelet Disord 2009, 10(1):76. https://doi.org/10.1186/1471-2474-10-76.

105. Cals JW, Hopstaken RM, Butler CC, Hood K, Severens JL, Dinant GJ. Improving management of patients with acute cough by C-reactive protein point of care testing and communication training (IMPAC3T): study protocol of a cluster randomised controlled trial. BMC Fam Pract 2007, 8:15. <https://doi.org/1471-2296-8-15>.

106. Abbott JH, Clare MC, McKenzie JE, David GD, Theis JC, Campbell AJ. Exercise therapy, manual therapy, or both, for osteoarthritis of the hip or knee: A factorial randomised controlled trial protocol. Trials 2009, 10(11)

107. Kyriakides TC, Babiker A, Singer J, Cameron W, Schechter MT, Holodniy M, Brown ST, Youle M, Gazzard B. An open-label randomized clinical trial of novel therapeutic strategies for HIV-infected patients in whom antiretroviral therapy has failed: rationale and design of the OPTIMA Trial. Control Clin Trials 2003, 24(4):481-500. <https://doi.org/S0197245603000291>.

108. Barber MD, Brubaker L, Menefee S, Norton P, Borello-France D, Varner E, Schaffer J, Weidner A, Xu X, Spino C *et al*. Operations and pelvic muscle training in the management of apical support loss (OPTIMAL) trial: design and methods. Contemp Clin Trials 2009, 30(2):178-189. https://doi.org/10.1016/j.cct.2008.12.001.

109. Kaner E, Bland M, Cassidy P, Coulton S, Deluca P, Drummond C, Gilvarry E, Godfrey C, Heather N, Myles J *et al*. Screening and brief interventions for hazardous and harmful alcohol use in primary care: A cluster randomised controlled trial protocol. BMC Public Health 2009, 9(287)

110. Coleman T, McEwen A, Bauld L, Ferguson J, Lorgelly P, Lewis S. Protocol for the Proactive Or Reactive Telephone Smoking CeSsation Support (PORTSSS) trial. Trials 2009, 10:26. [https://doi.org/](https://doi.org/1745-6215-10-26)10.1186/1745-6215-10-26.

111. Andrews PJ, Avenell A, Noble DW, Campbell MK, Battison CG, Croal BL, Simpson WG, Norrie J, Vale LD, Cook J *et al*. Randomised trial of glutamine and selenium supplemented parenteral nutrition for critically ill patients. Protocol Version 9, 19 February 2007 known as SIGNET (Scottish Intensive care Glutamine or seleNium Evaluative Trial). Trials 2007, 8(1):25. [https://doi.org/](https://doi.org/1745-6215-8-25)10.1186/1745-6215-8-25.

112. Mihaylov S, Stark C, McColl E, Steen N, Vanoli A, Rubin G, Curless R, Barton R, Bond J. Stepped treatment of older adults on laxatives. The STOOL trial. Health Technol Assess 2008, 12(13):iii-iv, ix-139. <https://doi.org/98/32/99>.

113. Ayles HM, Sismanidis C, Beyers N, Hayes RJ, Godfrey-Faussett P. ZAMSTAR, The Zambia South Africa TB and HIV Reduction study: Design of a 2 x 2 factorial community randomized trial. Trials 2008, 9(63)
