# Supplementary material for: Which interactions matter in economic evaluations? A systematic review and simulation study
Source: BMC Med Res Methodol. 2020 May 7;20:109. doi: 10.1186/s12874-020-00978-0 (PMC7203889; doi:10.1186/s12874-020-00978-0)
Supplement: Supplementary file 3 — Additional file 3. Additional methods on data simulation and analysis. Includes data on the magnitude of interactions for each of the studies reporting mean costs and mean health benefits for each cell within the factorial design, data inputs used in the simulation study and the Stata code used for statistical analysis. [file 12874_2020_978_MOESM3_ESM.docx]

**Additional file 3: Additional methods on data simulation and analysis**

**Data inputs used in the simulations**

Means and standard deviations (SDs) for each treatment group were extracted for each study and used to estimate interaction terms and treatment effects (Table 3.1). Across all trials in Table 3.1, four trials measured health benefits in QALYs, one used life-years gained, four used functioning/language scores and seven used the proportion of patients meeting endpoints. Net benefits for studies that did not use QALYs or life years were calculated using an arbitrary threshold of £5000 per unit of benefit (regardless of what those units were) in the absence of any evidence on the value that society might place on these benefits. Using an arbitrary threshold that was lower than the £20,000 ceiling ratio used for QALYs and using the same threshold for all endpoints enabled us to quantify the magnitude of interactions on a net benefit scale and conduct our simulations to explore how the results might change with different assumptions about interactions.

Since none of the published trials reported the correlation between costs and benefits, hypothetical correlation coefficients were estimated based on the clinical area and any scatter graphs reported. The Stata code used to generate simulated trials and analyse the results is shown below. Costs and health benefits were randomly drawn for each patient in each sample. For simplicity, studies were simulated as perfectly balanced, with equal patient numbers in each treatment arm.

For simplicity, benefits were simulated as normal distributions, allowing for the correlation between costs and benefits. However, a maximum benefit was also specified for each trial (Table 3.2), to ensure that percentages remain <100% and that no patient accrued >1 QALY per year of analysis.[[1]](#footnote-1) Any patient with sampled health benefits above the ceiling for that study had benefits reduced to the ceiling. Such ceiling effects introduce a small skew into benefits and could introduce or accentuate interactions, since the treatment group with highest benefits will be truncated more than other groups. An arbitrarily high limit of 50 units was therefore used for all scenarios assuming zero interaction.

Costs were simulated as gamma distributions defined by the mean cost for each treatment arm and its SD. Although SDs for health benefits were approximately homoskedastic across study arms, SDs for cost varied substantially between treatment arms in all but one study.[[2]](#footnote-2) For three studies, SDs were assumed to be proportional to the mean (Table 3.2). For two further studies[[3]](#footnote-3) the specific pattern of heteroskedasticity was modelled by using different SD:mean ratios for patients with and without treatment B.

Assuming that health benefits are continuous and normally distributed means that the data generating process does not realistically replicate the two studies with binomial benefit measures that reported SEs in each group. In principle, health benefits for these studies could have been modelled as a binomial distribution correlated with the cost distribution that modelled the effect of treatment and interactions on a logarithmic scale (e.g. as odds ratios). However, this would have greatly complicated data generation and would have meant that interactions were modelled (and would need to be analysed) on a logarithmic scale rather than a natural scale. By modelling these two trials as though they measured benefits on a continuous scale, this analysis essentially treats them as though they had actually measured benefits in QALYs.

Sampling costs for each patient from a gamma distribution defined by the mean and SE in each group also has limitations for interactions that arise from rare events with substantial cost implications. Our approach would have applied the interaction equally to all patients in all samples, which may not have realistically replicated the trial data in cases where the interaction was solely due to rare events occurring in one of the four treatment arms. By contrast, if we had simulated our data in a way that reflected the fact that the interaction arose because of rare events in a minority of people, we would have got much larger interactions in some samples (e.g. those that had a larger than average proportion of patients with the rare event) and additive effects in others (which sampled no patients with the rare event). This could have affected the proportion of simulations where interactions were included. By assuming that all costs followed a gamma distribution, we could have also underestimated the right tail of the cost distribution for high-cost rare events.

**Table 3.1** Magnitude of interactions for the 16 studies reporting mean costs and mean health benefits for each cell within the factorial design

| **Study** | **Description** | | **Treatment with highest expected NMB** | | **Interaction:effect ratio^a^ (p-value^b^)** | | |
| --- | --- | --- | --- | --- | --- | --- | --- |
|  |  |  | **Ignoring interactions** | **Allowing for interactions** | **Cost** | **Health benefit** | **NMB^c^** |
| ***Studies reporting standard deviations around costs and benefits for each cell*** | | | | | | | |
| AFIST II [1] | 2x2 evaluating amiodarone vs. placebo and pacing vs. no pacing. Cost per case of atrial fibrillation avoided | | Amiodarone + pacing | Amiodarone + pacing | -1.589 (0.314)^d^ | -7.000 (0.329)^d^ | -1.667 (0.276)^d^ |
| ATEAM [2] | 2x4 evaluating normal care vs. massage (M) vs. 6 lessons in Alexander technique (6A) vs. 24 lessons in the Alexander technique (24A) and exercise and behavioural counselling (E) vs. no exercise/counselling. Cost per QALY | M x E: | 24 Alexander lessons + exercise | 24 Alexander lessons + exercise | -0.909 (0.252) | -0.500 (0.265)^d^ | -0.442 (0.409)^d^ |
|  |  | 6A x E: |  |  | -0.476 (0.328) | -0.333 (0.579) | -0.280 (0.745) |
|  |  | 24A x E: |  |  | -0.489 (0.539) | 0.000 (1.00) | 0.110 (0.897) |
| Boyle [3] | 2x2 evaluating group vs. individual therapy by therapist vs. assistant. Cost per 1-point change in CELF-3 total language score | | Therapist, group | Therapist, group | 8.385 (0.002) | -2.372 (0.704) | -2.676 (0.698) |
| Hollis 2007 [4] | 2x3 evaluating brief vs. moderate vs. intensive telephone counselling and offer of free nicotine replacement therapy (NRT) patches vs. no offer. Cost per patient quitting smoking | NRT x Moderate: | Intensive, with NRT | Intensive, with NRT | 0.225 (0.044) | 0.429 (0.726) | 0.554 (0.780) |
|  |  | NRT x Intensive: |  |  | 0.154 (0.041) | 0.577 (0.561) | 1.000 (0.616) |
| NIMH-MTA [5] | 2x2 evaluating intensive medication management vs. not and multi-component behavioural treatment vs. not. Cost per 1 point change in functioning score | | Medical management | Medical management | 231.750 (0.005) | -1.600 (0.599) | -1.082 (0.238)^d^ |
| UKBEAM [6] | 2x2 evaluating spinal manipulation vs. no manipulation and exercise programme, vs. best care. Cost per QALY | | **Best care + manipulation + exercise** | **Best care + manipulation** | -1.500 (0.008) | -1.471 (0.348) | -1.450 (0.595) |
| ***Studies reporting means but not standard deviations for costs and benefits within each cell*** | | | | | | | |
| BELLS [7] | 2x2 evaluating prednisolone vs. placebo and aciclovir vs. placebo. Cost per QALY | | Prednisolone | Prednisolone | -0.059^d^ | -0.553^d^ | -0.017^d^ |
| Salize [8] | 2x2 evaluating training and incentive for general practitioners vs. none and training and medication for medication vs. none. Cost per patient abstaining from smoking | | Treatment as usual | Treatment as usual | 1.690 | 2.125 | 1.681 |
| ADAPT [9] | 2x2 evaluating exercise vs. no exercise programme and dietary counselling vs. no dietary counselling. Cost per 100% change in WOMAC | | Control | Control | 0.200 | -11.333^d^ | 0.045 |
| ASCOT UK results [10] | 2x2 partial factorial evaluating amlodipine (+perindopril as required) vs. atenolol (+bendroflumethiazide) and atorvastatin vs. placebo. Cost per QALY | | Amlodipine + atorvastatin | Amlodipine + atorvastatin | -0.082 | 1.000 | 13.000 |
| Cantor [11] | 2x2x2 evaluating Exit interview (exit) vs. no exit interview, home interview (home) vs. no interview, small-group sessions with a social worker (group) vs. no sessions. Cost per life-year gained | Exit x Home: | Exit + group | Exit + group | -1.218 | -2.511 | -2.666 |
|  |  | Exit x Group: |  |  | -0.750 | -0.679 | -0.678 |
|  |  | Home x Group: |  |  | -2.125 | -0.044 | 0.017 |
|  |  | 3-way: |  |  | 0.938 | 0.244 | 0.236 |
| Brandon [12] | 2x2 evaluating 8 vs. 1 mailing of 8 vs. 1 booklets to prevent relapse of smoking. Cost per 24-month smoking abstinence | | 8 mailings 8 booklets | 8 mailings 8 booklets | -0.156 | -0.667 | -0.816 |
| Barnett [13] | 2x2 evaluating voucher for free methadone treatment vs. no voucher and case management vs. none. Cost per 1 unit reduction in heroin use at 3 months | | Voucher | Voucher | -0.565 | -0.032^d^ | -0.003^d^ |
| Saillour-Glenisson [14] | 2x2 evaluating pocket card aide memoir [PAM] vs. no card and informative test request form vs. no form [form]. Inside-the-table results presented separated into 3 types of centre. Cost per test conforming with regulations | Specialist centres: | PAM+form | PAM+form | 0.028 | 0.000 | -0.013 |
|  |  | Small centres: | PAM | Control | 0.042 | -23.000 | -44.269 |
|  |  | Large centres: | Form | Form | -0.026 | -1.222 | -1.188^d^ |
| Bankhead [15] | 2x2 evaluating screening reminder letter from GP vs. no letter and flag in notes prompting discussion of screening, vs. no flag. Cost per additional woman screened | | Letter + flag | Letter + flag | -0.231 | -0.514 | -0.517 |
| Richards [15] | 2x2 evaluating screening reminder letter from GP vs. no letter and flag in notes prompting discussion of screening, vs. no flag. Cost per additional woman screened | | Letter + flag | Letter + flag | -0.471 | -0.789 | -0.794 |

Abbreviations: AFIST, Atrial Fibrillation Suppression Trial; ASCOT, Anglo-Scandinavian Cardiac Outcomes Trial; ADAPT, Arthritis, Diet, and Activity Promotion Trial; ATEAM, Alexander Technique, Exercise and Massage; BELLS, Bell’s palsy: Early acicLovir and/or prednisolone in Scotland; CELF-3, Clinical Evaluation of Language Fundamentals; GP, general practitioner; NIMH-MTA, National Institute of Mental Health Multimodal Treatment study of children with Attention deficit and hyperactivity disorder; UKBEAM, United Kingdom Back pain Exercise And Manipulation; WOMAC, Western Ontario and McMaster [Osteoarthritis Index].

^a^ For two-way interactions, the interaction:effect ratio ($IER_{AB}$) was calculated as the interaction term ($I_{AB}$) divided by the simple effect of A ($\delta_{A}$): i.e. as $IER_{AB}=I_{AB}/\delta_{A}=\left(\mu_{0}-\mu_{a}-\mu_{b}+\mu_{ab}\right)/\left(\mu_{a}-\mu_{0}\right)$, where the factor defined as A is chosen such that $\left|\mu_{a}-\mu_{0}\right|<\left|\mu_{b}-\mu_{0}\right|$ in the case of non-mixed interactions and such that $\delta_{A}$ has the opposite sign to $I_{AB}$ in the case of mixed interactions. For three-way interactions, the interaction:effect ratio was calculated as: $I_{ABC}\left[\mu_{abc}-\left(\mu_{0}+\delta_{A}+\delta_{B}+\delta_{C}+I_{AB}+I_{AC}+I_{BC}\right)\right]/\delta_{A}$, where $\delta_{A}<\delta_{B}$ and $\delta_{A}<\delta_{C}$for non-mixed interactions and, for mixed interactions, where $\delta_{A}$ is the smallest of the simple effects that has the opposite sign to the interaction term.

^b^ The p-value around the interaction term was calculated in Microsoft Excel using a two-tailed t-test of the ratio of mean interaction/standard error around the mean (i.e. $\left|I_{AB}/SE_{\mathrm{int}AB}\right|$); the t-test was conducted with $n_{0}+n_{a}+n_{b}+n_{ab}-4$ degrees of freedom. The standard error for the interaction was calculated as $4\cdot\left(\sum_{k=0,a,b,ab} \left(n_{k}-1\right)\cdot s_{k}^{2}\right)^{1/2}\div\left[2\cdot\left(\sum_{k=0,a,b,ab} \left(n_{k}\right)\right)^{1/2}+\sum_{k=0,a,b,ab} \left(n_{k}\right)\right]$ , where $n_{k}$ equals the number of patients in arm k and $s_{k}$equals the standard deviation in arm k.

^c^ A £20,000/QALY (or $20,000/QALY or €20,000/QALY) ceiling ratio was used for QALYs [16] and life-years gained, while a ceiling ratio of £5,000 (or $5,000 or €5,000) per unit of benefit was arbitrarily used for all other health benefits.

^d^ Mixed interaction: one factor increases the outcome of interest, while the other decreases it. The interaction therefore cannot be classified as either sub-additive or super-additive.

**Table 3.2** Data inputs for each study included in the simulation. The data inputs shown in this table were kept constant across all simulated datasets from the same trial.

| **Study** | **Constant: cost** | **dA: cost** | **dB: cost** | **Type of cost distribution** | **SD: cost** | **Units for health benefits** | **Constant: health** | **dA: health** | **dB: health** | **SD: health** | **Max health units** | **Correlation between costs and health** |
| --- | --- | --- | --- | --- | --- | --- | --- | --- | --- | --- | --- | --- |
| AFIST II [1] | £27,026 | £6,842 | -£4,301 | Heteroskedastic | SDMR A: 1.398; SDMR B: 0.609 | % pts with atrial fibrillation§ | 0.380§ | 0.020§ | -0.100§ | 0.449 | 1 | 0.30* |
| ATEAM [2] | £54 | £204 | £100 | Heteroskedastic | SDMR A: 2.617; SDMR B: 1.070 | QALY | 0.700 | -0.010 | 0.040 | 0.087 | 1 | -0.10* |
| Boyle [3] | £493 | £26 | £407 | Ratio | SDMR: 0.249 | Language score | 1.590 | 2.910 | 0.860 | 7.365 | 50‡ | 0.05* |
| Hollis 2007 [4] | £67 | £40 | £126 | Ratio | SDMR: 0.349 | % smoking abstinence | 0.117 | 0.021 | 0.054 | 0.360 | 1 | 0.15* |
| NIMH-MTA [5] | £975 | £4 | £5,158 | Homoskedastic | SD: 917 | Functioning Score§ | -0.600§ | -0.320§ | -0.100§ | 0.898 | 15‡ | 0.30* |
| UKBEAM [6] | £346 | £140 | £195 | Ratio | SDMR: 1.509 | QALY | 0.618 | 0.017 | 0.041 | 0.239 | 1 | -0.10* |

Abbreviations: AFIST, Atrial Fibrillation Suppression Trial; ATEAM, Alexander Technique, Exercise and Massage; dA [dB], simple effect of treatment A [B] $(\mu_{a}-\mu_{0})$; NIMH-MTA, National Institute of Mental Health Multimodal Treatment study of children with ADHD; SDMR, ratio of mean divided by SD; UKBEAM, United Kingdom Back pain Exercise And Manipulation.

* Hypothetical figure informed by the clinical area and any available scatter graphs.

† Estimated from primary data.

‡ Arbitrary limit chosen to be well above means for each group, since no maximum score was indicated in the trial reports.

§ AFIST and NIMH-MTA measured health benefits on scales in which high values indicate worse scores rather than better scores. The trials were simulated and mixed models were estimated using the actual scores (i.e. the proportion of patients with atrial fibrillation and negative functioning scores). However, to ensure that NMB was calculated correctly, model coefficients were transformed before NMB was calculated or criteria for assessing interactions were applied. For both trials, all interaction terms and treatment effects were subtracted from 0 to change the sign. Constant terms for NIMH-MTA were subtracted from zero, while those for AFIST were subtracted from 1. This transformation has no effect on AIC, BIC, SEs, degrees of freedom or p-values, so these outcomes were not adjusted.

¥ For IVAN, the interaction between drug and treatment regimen is so large that simply setting the interaction to 0 generates negative costs (Chapter 7). To avoid this arising within the simulated data, the constant term was increased by £2,000 in the two scenarios with zero interaction.

**Table 3.3** Data inputs used for each simulated version of each study

| **Scenario Name** | **c_Int** | **q_Int** | **Patients /arm** | **Best Tx*** | **Interaction:effect ratio†** | | |
| --- | --- | --- | --- | --- | --- | --- | --- |
|  |  |  |  |  | **Cost** | **QALYs** | **NMB*** |
| AFIST | -£10,870 | -14% | 40 | *ab* | -1.589 | -7.000 | -1.667 |
| AFIST_DoubleInt | -£21,740 | -28% | 40 | *ab* | -3.177 | -14.000 | -3.333 |
| AFIST_HalfInt | -£5,435 | -7% | 40 | *b* | -0.794 | -3.500 | -0.833 |
| AFIST_DoubleSize | -£10,870 | -14% | 80 | *ab* | -1.589 | -7.000 | -1.667 |
| AFIST_NoInt | £0 | 0% | 40 | *b* | 0.000 | 0.000 | 0.000 |
| AFIST_NoInt_DoubleSize | £0 | 0% | 80 | *b* | 0.000 | 0.000 | 0.000 |
| ATEAM | -£91 | -0.02 | 73 | *b* | -0.909 | -0.500 | -0.442 |
| ATEAM_DoubleInt | -£182 | -0.04 | 73 | *b* | -1.818 | -1.000 | -0.883 |
| ATEAM_HalfInt | -£45 | -0.01 | 73 | *b* | -0.454 | -0.250 | -0.221 |
| ATEAM_DoubleSize | -£91 | -0.02 | 146 | *b* | -0.909 | -0.500 | -0.442 |
| ATEAM_NoInt | £0 | 0 | 73 | *b* | 0.000 | 0.000 | 0.000 |
| ATEAM_NoInt_DoubleSize | £0 | 0 | 146 | *b* | 0.000 | 0.000 | 0.000 |
| Boyle | £218 | -2.04 | 31 | *a* | 8.385 | -2.372 | -2.676 |
| Boyle_DoubleInt | £436 | -4.08 | 31 | *a* | 16.769 | -4.744 | -5.352 |
| Boyle_HalfInt | £109 | -1.02 | 31 | *a* | 4.192 | -1.186 | -1.338 |
| Boyle_DoubleSize | £218 | -2.04 | 62 | *a* | 8.385 | -2.372 | -2.676 |
| Boyle_NoInt | £0 | 0 | 31 | *ab* | 0.000 | 0.000 | 0.000 |
| Boyle_NoInt_DoubleSize | £0 | 0 | 62 | *ab* | 0.000 | 0.000 | 0.000 |
| Hollis | £9 | 1% | 793 | *ab* | 0.225 | 0.429 | 0.554 |
| Hollis_DoubleInt | £18 | 2% | 793 | *ab* | 0.450 | 0.857 | 1.108 |
| Hollis_HalfInt | £5 | 0% | 793 | *ab* | 0.113 | 0.214 | 0.277 |
| Hollis_DoubleSize | £9 | 1% | 1586 | *ab* | 0.225 | 0.429 | 0.554 |
| Hollis_NoInt | £0 | 0% | 793 | *ab* | 0.000 | 0.000 | 0.000 |
| Hollis_NoInt_DoubleSize | £0 | 0% | 1586 | *ab* | 0.000 | 0.000 | 0.000 |
| NIMH-MTA | £927 | -0.16 | 35 | *0* | 231.750 | -1.600 | 0.079 |
| NIMH-MTA_DoubleInt | £1,854 | -0.32 | 35 | *0* | 463.500 | -3.200 | 0.158 |
| NIMH-MTA_HalfInt | £464 | -0.08 | 35 | *0* | 115.875 | -0.800 | 0.040 |
| NIMH-MTA_DoubleSize | £927 | -0.16 | 70 | *0* | 231.750 | -1.600 | 0.079 |
| NIMH-MTA_NoInt | £0 | 0 | 35 | *0* | 0.000 | 0.000 | 0.000 |
| NIMH-MTA_NoInt_DoubleSize | £0 | 0 | 70 | *0* | 0.000 | 0.000 | 0.000 |
| UKBEAM | -£210 | -0.025 | 322 | *b* | -1.500 | -1.471 | -1.450 |
| UKBEAM_DoubleInt | -£420 | -0.05 | 322 | *b* | -3.000 | -2.941 | -2.900 |
| UKBEAM_HalfInt | -£105 | -0.0125 | 322 | *ab* | -0.750 | -0.735 | -0.725 |
| UKBEAM_DoubleSize | -£210 | -0.025 | 644 | *b* | -1.500 | -1.471 | -1.450 |
| UKBEAM_NoInt | £0 | 0 | 322 | *ab* | 0.000 | 0.000 | 0.000 |
| UKBEAM_NoInt_DoubleSize | £0 | 0 | 644 | *ab* | 0.000 | 0.000 | 0.000 |

Abbreviations: AFIST, Atrial Fibrillation Suppression Trial; ATEAM, Alexander Technique, Exercise and Massage; c_Int, interaction for cost; NIMH-MTA, National Institute of Mental Health Multimodal Treatment study of children with ADHD; q_Int, interaction for health benefits; Tx, treatment-combination; UKBEAM, United Kingdom Back pain Exercise And Manipulation.

* NMB was calculated at a £20,000/QALY ceiling ratio; a £5,000 (or $5,000) per unit ceiling ratio was used for all other measures of health benefit.

† The interaction:effect ratio ($IER_{AB}$) was calculated as the ratio of the interaction ($I_{AB}$) divided by the main effect of A ($\delta_{A}$): i.e. as $IER_{AB}=I_{AB}/\delta_{A}=\left(\mu_{0}-\mu_{a}-\mu_{b}+\mu_{ab}\right)/\left(\mu_{a}-\mu_{0}\right)$, where the factor defined as A is chosen such that $\left|\mu_{a}-\mu_{0}\right|<\left|\mu_{b}-\mu_{0}\right|$ in the case of non-mixed interactions and such that $\delta_{A}$ has the opposite sign to $I_{AB}$ in the case of mixed interactions.

**Stata code used in the simulation study**

The below Stata code runs using the file “Online Resource 5 - Data for simulation.xlsx”.

import excel " Online Resource 5 - Data for simulation.xlsx", sheet("Sheet1") firstrow

sum NoPts

local I=r(N)

mkmat Study c_cons c_A c_B c_Int c_DistType c_sd c_SDOverMean c_sdOverMeanB0 c_sdOverMeanB1 Outcome q_cons q_A q_B q_Int q_sd q_limit rho NoPts, matrix(Data)

clear

local N=300 //Number of datasets to generate with each combination of interaction terms across all sample sizes

forvalues n=1(1)`N' {

forvalues i=1(1)`I' {

display "Dataset `i', Run `n'"

quietly {

clear

local w=1

local variable "Study c_cons c_A c_B c_Int c_DistType c_sd c_SDOverMean c_sdOverMeanB0 c_sdOverMeanB1 Outcome q_cons q_A q_B q_Int q_sd q_limit rho NoPts"

foreach v of local variable {

local `v' = Data[`i',`w']

local w=`w'+1

}

//GENERATING A DATASET

local NoPtsTotal = `NoPts'*4

set obs `NoPtsTotal' //Total number of patients in trial

generate ptid=_n

generate treatment=ceil(ptid/`NoPts')

generate a=0

replace a=1 if treatment==2 | treatment==4

generate b=0

replace b=1 if treatment==3 | treatment==4

generate interaction = a*b

generate mean_cost = `c_cons' + a*`c_A' + b*`c_B' + interaction*`c_Int'

if (`c_DistType'==1) generate sd_cost = `c_sd' //Homoskedastic

if (`c_DistType'==2) generate sd_cost = `c_SDOverMean'*mean_cost //Ratio.

if (`c_DistType'==3) generate sd_cost = `c_sdOverMeanB0'*mean_cost if b==0 //Heteroskedastic ratio

if (`c_DistType'==3) replace sd_cost = `c_sdOverMeanB1'*mean_cost if b==1 //Heteroskedastic ratio

generate cost = rgamma((mean_cost/sd_cost)^2,(sd_cost^2)/mean_cost)

generate rand_qalys = rnormal()

generate rand_cost_standard=(cost-mean_cost)/sd_cost

generate mean_qalys = `q_cons' + a*`q_A' + b*`q_B' + interaction*`q_Int'

generate qalys = `q_cons' + a*`q_A' + b*`q_B' + interaction*`q_Int' + `q_sd'*(rand_cost_standard*`rho' + rand_qalys*(1-`rho'^2)^0.5)

replace qalys = `q_limit' if qalys>`q_limit' //Imposing a ceiling effect on QALYs

sum cost qalys if treatment==1 //Means should equal c_cons SD should equal c_sd

sum cost qalys if treatment==2 //Means should equal c_cons + c_A SD should equal c_sd

sum cost qalys if treatment==3 //Means should equal c_cons + c_B SD should equal c_sd

sum cost qalys if treatment==4 //Means should equal c_cons + c_A + c_B + c_Int; SD should equal c_sd

correl cost qalys //Correlation should equal rho

regress cost a b interaction //coefficients should equal parameters set above; SE for constant should equal SD/sqrt(n*4), SE for A & B should be SD/sqrt(n*2), SE for interaction should be SD/sqrt(n)

regress qalys a b interaction //coefficients should equal parameters set above

rename qalys outcome1

rename cost outcome2

reshape long outcome, i(ptid) j(cost)

replace cost=cost-1 //Variable called cost has been generated in the reshape process, but is currently equal to 1 if outcome=health, and we want it to equal 1 if outcome=cost

tab cost

sum outcome if cost==1

sum outcome if cost==0

generate A_cost = cost* a

generate B_cost = (cost)* b

generate Interaction_cost = (cost)* interaction

generate A_QALY = (1-cost)* a

generate B_QALY = (1-cost)* b

generate Interaction_QALY = (1-cost)* interaction

generate QALYconstant=1-cost

generate cost_noint = 0

generate qaly_noint = 0

xtmixed outcome cost A_cost B_cost cost_noint QALYconstant A_QALY B_QALY qaly_noint , noconstant || ptid: , noconstant residuals(unstructured, t(cost))

matrix coefficients_aux = e(b)

matrix coleq coefficients_aux =""

matrix colnames coefficients_aux = C_cons_cN_qN C_A_cN_qN C_B_cN_qN C_Int_cN_qN Q_cons_cN_qN Q_A_cN_qN Q_B_cN_qN Q_Int_cN_qN C_SD_cN_qN Q_SD_cN_qN CQ_SD_cN_qN

scalar C_Int_test_cN_qN = coefficients_aux[1, 4]

scalar Q_Int_test_cN_qN = coefficients_aux[1, 8]

matrix V=e(V)

scalar C_Int_SE_cN_qN=sqrt(V[4,4])

scalar Q_Int_SE_cN_qN=sqrt(V[8,8])

scalar C_A_SE_cN_qN=sqrt(V[2,2])

scalar Q_A_SE_cN_qN=sqrt(V[6,6])

scalar C_B_SE_cN_qN=sqrt(V[3,3])

scalar Q_B_SE_cN_qN=sqrt(V[7,7])

matrix Int = C_Int_test_cN_qN, C_Int_SE_cN_qN, C_A_SE_cN_qN, C_B_SE_cN_qN, Q_Int_test_cN_qN, Q_Int_SE_cN_qN, Q_A_SE_cN_qN, Q_B_SE_cN_qN

matrix colnames Int = C_Int_test_cN_qN C_Int_SE_cN_qN C_A_SE_cN_qN C_B_SE_cN_qN Q_Int_test_cN_qN Q_Int_SE_cN_qN Q_A_SE_cN_qN Q_B_SE_cN_qN

matrix LL = e(df_m), e(ll), e(r2_a)

matrix colnames LL = dfm_cN_qN LL_cN_qN R2Adj_cN_qN

matrix coefficients_all = coefficients_aux, Int, LL

xtmixed outcome cost A_cost B_cost Interaction_cost QALYconstant A_QALY B_QALY qaly_noint , noconstant || ptid: , noconstant residuals(unstructured, t(cost))

matrix coefficients_aux = e(b)

matrix coleq coefficients_aux =""

matrix colnames coefficients_aux = C_cons_cI_qN C_A_cI_qN C_B_cI_qN C_Int_cI_qN Q_cons_cI_qN Q_A_cI_qN Q_B_cI_qN Q_Int_cI_qN C_SD_cI_qN Q_SD_cI_qN CQ_SD_cI_qN

scalar C_Int_test_cI_qN = coefficients_aux[1, 4]

scalar Q_Int_test_cI_qN = coefficients_aux[1, 8]

matrix V=e(V)

scalar C_Int_SE_cI_qN=sqrt(V[4,4])

scalar Q_Int_SE_cI_qN=sqrt(V[8,8])

scalar C_A_SE_cI_qN=sqrt(V[2,2])

scalar Q_A_SE_cI_qN=sqrt(V[6,6])

scalar C_B_SE_cI_qN=sqrt(V[3,3])

scalar Q_B_SE_cI_qN=sqrt(V[7,7])

matrix Int = C_Int_test_cI_qN, C_Int_SE_cI_qN, C_A_SE_cI_qN, C_B_SE_cI_qN, Q_Int_test_cI_qN, Q_Int_SE_cI_qN, Q_A_SE_cI_qN, Q_B_SE_cI_qN

matrix colnames Int = C_Int_test_cI_qN C_Int_SE_cI_qN C_A_SE_cI_qN C_B_SE_cI_qN Q_Int_test_cI_qN Q_Int_SE_cI_qN Q_A_SE_cI_qN Q_B_SE_cI_qN

matrix LL = e(df_m), e(ll), e(r2_a)

matrix colnames LL = dfm_cI_qN LL_cI_qN R2Adj_cI_qN

matrix coefficients_all = coefficients_all , coefficients_aux, Int, LL

xtmixed outcome cost A_cost B_cost cost_noint QALYconstant A_QALY B_QALY Interaction_QALY, noconstant || ptid: , noconstant residuals(unstructured, t(cost))

matrix coefficients_aux = e(b)

matrix coleq coefficients_aux =""

matrix colnames coefficients_aux = C_cons_cN_qI C_A_cN_qI C_B_cN_qI C_Int_cN_qI Q_cons_cN_qI Q_A_cN_qI Q_B_cN_qI Q_Int_cN_qI C_SD_cN_qI Q_SD_cN_qI CQ_SD_cN_qI

scalar C_Int_test_cN_qI = coefficients_aux[1, 4]

scalar Q_Int_test_cN_qI = coefficients_aux[1, 8]

matrix V=e(V)

scalar C_Int_SE_cN_qI=sqrt(V[4,4])

scalar Q_Int_SE_cN_qI=sqrt(V[8,8])

scalar C_A_SE_cN_qI=sqrt(V[2,2])

scalar Q_A_SE_cN_qI=sqrt(V[6,6])

scalar C_B_SE_cN_qI=sqrt(V[3,3])

scalar Q_B_SE_cN_qI=sqrt(V[7,7])

matrix Int = C_Int_test_cN_qI, C_Int_SE_cN_qI, C_A_SE_cN_qI, C_B_SE_cN_qI, Q_Int_test_cN_qI, Q_Int_SE_cN_qI, Q_A_SE_cN_qI, Q_B_SE_cN_qI

matrix colnames Int = C_Int_test_cN_qI C_Int_SE_cN_qI C_A_SE_cN_qI C_B_SE_cN_qI Q_Int_test_cN_qI Q_Int_SE_cN_qI Q_A_SE_cN_qI Q_B_SE_cN_qI

matrix LL = e(df_m), e(ll), e(r2_a)

matrix colnames LL = dfm_cN_qI LL_cN_qI R2Adj_cN_qI

matrix coefficients_all = coefficients_all , coefficients_aux, Int, LL

xtmixed outcome cost A_cost B_cost Interaction_cost QALYconstant A_QALY B_QALY Interaction_QALY, noconstant || ptid: , noconstant residuals(unstructured, t(cost))

matrix coefficients_aux = e(b)

matrix coleq coefficients_aux =""

matrix colnames coefficients_aux = C_cons_cI_qI C_A_cI_qI C_B_cI_qI C_Int_cI_qI Q_cons_cI_qI Q_A_cI_qI Q_B_cI_qI Q_Int_cI_qI C_SD_cI_qI Q_SD_cI_qI CQ_SD_cI_qI

scalar C_Int_test_cI_qI = coefficients_aux[1, 4]

scalar Q_Int_test_cI_qI = coefficients_aux[1, 8]

matrix V=e(V)

scalar C_Int_SE_cI_qI=sqrt(V[4,4])

scalar Q_Int_SE_cI_qI=sqrt(V[8,8])

scalar C_A_SE_cI_qI=sqrt(V[2,2])

scalar Q_A_SE_cI_qI=sqrt(V[6,6])

scalar C_B_SE_cI_qI=sqrt(V[3,3])

scalar Q_B_SE_cI_qI=sqrt(V[7,7])

matrix Int = C_Int_test_cI_qI, C_Int_SE_cI_qI, C_A_SE_cI_qI, C_B_SE_cI_qI, Q_Int_test_cI_qI, Q_Int_SE_cI_qI, Q_A_SE_cI_qI, Q_B_SE_cI_qI

matrix colnames Int = C_Int_test_cI_qI C_Int_SE_cI_qI C_A_SE_cI_qI C_B_SE_cI_qI Q_Int_test_cI_qI Q_Int_SE_cI_qI Q_A_SE_cI_qI Q_B_SE_cI_qI

matrix LL = e(df_m), e(ll), e(r2_a)

matrix colnames LL = dfm_cI_qI LL_cI_qI R2Adj_cI_qI

matrix coefficients_all = coefficients_all , coefficients_aux, Int, LL

clear

svmat coefficients_all, names(col)

generate Dataset = `i'

generate run = `n'

local input = "NoPts rho c_cons c_A c_B c_Int c_sd q_cons q_A q_B q_Int q_sd"

foreach v of local input {

generate `v'=``v''

}

if (`i'==1 & `n'==1) save "Allcoefficients.dta", replace

if (`i'!=1 | `n'!=1) append using "Allcoefficients.dta"

if (`i'!=1 | `n'!=1) save "Allcoefficients.dta", replace

} //quietly

} //for i

} //for n

tab Dataset computer

order rho , first

order c_sd , first

order c_cons , first

order c_B , first

order c_A , first

order q_sd , first

order q_cons , first

order q_B , first

order q_A , first

order q_Int , first

order c_Int , first

order NoPts , first

order run, first

order Dataset, first

sort Dataset NoPts run

//Adjustment for outcomes in AFIST II and MTA where higher outcome numbers are bad. AFIST was analysed as % pts with AF & want to report as % pts without AF. MTA was analysed as negative numbers, but want to analyse as positive ones

sum q_cons Q_cons_cN_qN Q_cons_cN_qI Q_cons_cI_qN Q_cons_cI_qI q_A q_B q_Int Q_Int_cN_qN Q_A_cN_qN Q_B_cN_qN Q_Int_test_cN_qN Q_Int_cN_qI Q_A_cN_qI Q_B_cN_qI Q_Int_test_cN_qI Q_Int_cI_qN Q_A_cI_qN Q_B_cI_qN Q_Int_test_cI_qN Q_Int_cI_qI Q_A_cI_qI Q_B_cI_qI Q_Int_test_cI_qI if Dataset >=19 & Dataset<=24

sum q_cons Q_cons_cN_qN Q_cons_cN_qI Q_cons_cI_qN Q_cons_cI_qI q_A q_B q_Int Q_Int_cN_qN Q_A_cN_qN Q_B_cN_qN Q_Int_test_cN_qN Q_Int_cN_qI Q_A_cN_qI Q_B_cN_qI Q_Int_test_cN_qI Q_Int_cI_qN Q_A_cI_qN Q_B_cI_qN Q_Int_test_cI_qN Q_Int_cI_qI Q_A_cI_qI Q_B_cI_qI Q_Int_test_cI_qI if Dataset >=25 & Dataset<=30

sum q_cons Q_cons_cN_qN Q_cons_cN_qI Q_cons_cI_qN Q_cons_cI_qI q_A q_B q_Int Q_Int_cN_qN Q_A_cN_qN Q_B_cN_qN Q_Int_test_cN_qN Q_Int_cN_qI Q_A_cN_qI Q_B_cN_qI Q_Int_test_cN_qI Q_Int_cI_qN Q_A_cI_qN Q_B_cI_qN Q_Int_test_cI_qN Q_Int_cI_qI Q_A_cI_qI Q_B_cI_qI Q_Int_test_cI_qI if Dataset <19 | Dataset>30

local outcome="q_cons Q_cons_cN_qN Q_cons_cN_qI Q_cons_cI_qN Q_cons_cI_qI"

foreach o of local outcome {

replace `o'=1-`o' if Dataset >=19 & Dataset<=24 //AFIST: Constants are set to be 100% - % with AF

replace `o'=0-`o' if Dataset >=25 & Dataset<=30 //MTA: Constants are set to be - X

}

local outcome="q_A q_B q_Int"

foreach o of local outcome {

replace `o'=0-`o' if Dataset >=19 & Dataset<=30 //AFIST & MTA: Sign is changed on interaction terms & all treatment effects

}

local outcome="Q_Int Q_A Q_B Q_Int_test"

foreach o of local outcome {

local model="_cN_qN _cI_qN _cN_qI _cI_qI"

foreach m of local model {

replace `o'`m'=0-`o'`m' if Dataset >=19 & Dataset<=30 //AFIST & MTA: Sign is changed on interaction terms & all treatment effects

}

}

sum q_cons Q_cons_cN_qN Q_cons_cN_qI Q_cons_cI_qN Q_cons_cI_qI q_A q_B q_Int Q_Int_cN_qN Q_A_cN_qN Q_B_cN_qN Q_Int_test_cN_qN Q_Int_cN_qI Q_A_cN_qI Q_B_cN_qI Q_Int_test_cN_qI Q_Int_cI_qN Q_A_cI_qN Q_B_cI_qN Q_Int_test_cI_qN Q_Int_cI_qI Q_A_cI_qI Q_B_cI_qI Q_Int_test_cI_qI if Dataset >=19 & Dataset<=24

sum q_cons Q_cons_cN_qN Q_cons_cN_qI Q_cons_cI_qN Q_cons_cI_qI q_A q_B q_Int Q_Int_cN_qN Q_A_cN_qN Q_B_cN_qN Q_Int_test_cN_qN Q_Int_cN_qI Q_A_cN_qI Q_B_cN_qI Q_Int_test_cN_qI Q_Int_cI_qN Q_A_cI_qN Q_B_cI_qN Q_Int_test_cI_qN Q_Int_cI_qI Q_A_cI_qI Q_B_cI_qI Q_Int_test_cI_qI if Dataset >=25 & Dataset<=30

sum q_cons Q_cons_cN_qN Q_cons_cN_qI Q_cons_cI_qN Q_cons_cI_qI q_A q_B q_Int Q_Int_cN_qN Q_A_cN_qN Q_B_cN_qN Q_Int_test_cN_qN Q_Int_cN_qI Q_A_cN_qI Q_B_cN_qI Q_Int_test_cN_qI Q_Int_cI_qN Q_A_cI_qN Q_B_cI_qN Q_Int_test_cI_qN Q_Int_cI_qI Q_A_cI_qI Q_B_cI_qI Q_Int_test_cI_qI if Dataset <19 | Dataset>30

//CALCULATING CRITERIA FOR INTERACTIONS

local model="_cN_qN _cI_qN _cN_qI _cI_qI"

foreach m of local model {

generate aic`m' = 2*dfm`m'-2*LL`m'

generate bic`m' = -2*LL`m' + dfm`m'*ln(NoPts*4)

local outcome="C Q"

foreach o of local outcome {

generate `o'_pvalueInt`m' = ttail(NoPts*4-dfm`m', abs(`o'_Int`m'/`o'_Int_SE`m'))

generate `o'_pvalueA`m' = ttail(NoPts*4-dfm`m', abs(`o'_A`m' /`o'_A_SE`m'))

generate `o'_pvalueB`m' = ttail(NoPts*4-dfm`m', abs(`o'_B`m' /`o'_B_SE`m'))

egen minTxeffect = rowmin(`o'_A`m' `o'_B`m')

egen maxTxeffect = rowmax(`o'_A`m' `o'_B`m')

replace minTxeffect=maxTxeffect if abs(minTxeffect)>abs(maxTxeffect)

generate `o'_IntTyp`m' = 0 if `o'_Int`m'==0

replace `o'_IntTyp`m' = -1 if (`o'_Int`m'<0 & minTxeffect>=0) | (`o'_Int`m'>0 & minTxeffect<=0) //Sub-additive

replace `o'_IntTyp`m' = -2 if (`o'_Int`m'<0 & minTxeffect>=0 & abs(`o'_Int`m')>abs(minTxeffect)) | (`o'_Int`m'>0 & minTxeffect<=0 & abs(`o'_Int`m')>abs(minTxeffect)) //Qualitative

replace `o'_IntTyp`m' = 1 if (`o'_Int`m'>0 & minTxeffect> 0) | (`o'_Int`m'<0 & minTxeffect< 0) //Super-additive

replace `o'_IntTyp`m' = 2 if (`o'_Int`m'>0 & minTxeffect> 0 & abs(`o'_Int`m')>abs(minTxeffect)) | (`o'_Int`m'<0 & minTxeffect< 0 & abs(`o'_Int`m')>abs(minTxeffect)) //Super-additive & larger than main effect

replace `o'_IntTyp`m' = -3 if `o'_A`m'/`o'_B`m'<0 & `o'_Int`m'!=0 //Mixed interaction (could at this stage be qualitative or not)

replace `o'_IntTyp`m' = -4 if `o'_A`m'/`o'_B`m'<0 & `o'_Int`m'!=0 & ((`o'_Int`m'/`o'_A`m'<0 & abs(`o'_Int`m')>abs(`o'_A`m')) | (`o'_Int`m'/`o'_B`m'<0 & abs(`o'_Int`m')>abs(`o'_B`m'))) //Mixed qualitative interaction, where minTxeffect is the one with the opposing sign

generate `o'_IntRatio`m' = `o'_Int`m'/minTxeffect if `o'_IntTyp`m'>-3

replace `o'_IntRatio`m' = `o'_Int`m'/`o'_A`m' if `o'_IntTyp`m'<=-3 & `o'_Int`m'/`o'_A`m'<0

replace `o'_IntRatio`m' = `o'_Int`m'/`o'_B`m' if `o'_IntTyp`m'<=-3 & `o'_Int`m'/`o'_B`m'<0

drop minTxeffect maxTxeffect

label define `o'_IntTyp`m' -4 "Mixed Qualitative" -3 "Mixed Quant" -2 "Qualitative" -1 "SubAdd Quant" 0 "Zero" 1 "SuperAdd" 2 "LargeSuperAdd"

label values `o'_IntTyp`m' `o'_IntTyp`m'

generate `o'_Bias`m' = 0.5*`o'_Int`m'

}

forvalues R=0(5000)40000 {

generate NB_00`m'_`R' = (Q_cons`m' )*`R'-(C_cons`m')

generate NB_A0`m'_`R' = (Q_cons`m' + Q_A`m' )*`R'-(C_cons`m' + C_A`m')

generate NB_0B`m'_`R' = (Q_cons`m' + Q_B`m' )*`R'-(C_cons`m' + C_B`m')

generate NB_AB`m'_`R' = (Q_cons`m' + Q_A`m' + Q_B`m' + Q_Int`m')*`R'-(C_cons`m' + C_A`m' + C_B`m' + C_Int`m')

generate NB_Int`m'_`R'= NB_00`m'_`R' + NB_AB`m'_`R' - NB_A0`m'_`R' - NB_0B`m'_`R'

generate txA= NB_A0`m'_`R'-NB_00`m'_`R'

generate txB= NB_0B`m'_`R'-NB_00`m'_`R'

egen minTxeffect = rowmin(txA txB)

egen maxTxeffect = rowmax(txA txB)

replace minTxeffect=maxTxeffect if abs(minTxeffect)>abs(maxTxeffect)

generate NB_IntTyp`m'_`R' = 0 if NB_Int`m'_`R'==0

replace NB_IntTyp`m'_`R' = -1 if (NB_Int`m'_`R'<0 & minTxeffect>=0) | (NB_Int`m'_`R'>0 & minTxeffect<0)

replace NB_IntTyp`m'_`R' = -2 if (NB_Int`m'_`R'<0 & minTxeffect>=0 & abs(NB_Int`m'_`R')>abs(minTxeffect)) | (NB_Int`m'_`R'>0 & minTxeffect<0 & abs(NB_Int`m'_`R')>abs(minTxeffect))

replace NB_IntTyp`m'_`R' = 1 if (NB_Int`m'_`R'>0 & minTxeffect> 0) | (NB_Int`m'_`R'<0 & minTxeffect< 0)

replace NB_IntTyp`m'_`R' = 2 if (NB_Int`m'_`R'>0 & minTxeffect> 0 & abs(NB_Int`m'_`R')>abs(minTxeffect)) | (NB_Int`m'_`R'<0 & minTxeffect< 0 & abs(NB_Int`m'_`R')>abs(minTxeffect))

replace NB_IntTyp`m'_`R' = -3 if txA/txB<0 & NB_Int`m'_`R'!=0 //Mixed interaction (could at this stage be qualitative or not)

replace NB_IntTyp`m'_`R' = -4 if txA/txB<0 & NB_Int`m'_`R'!=0 & ((NB_Int`m'_`R'/txA<0 & abs(NB_Int`m'_`R')>abs(txA)) | (NB_Int`m'_`R'/txB<0 & abs(NB_Int`m'_`R')>abs(txB))) //Mixed qualitative interaction, where minTxeffect is the one with the opposing sign

generate NB_IntRatio`m'_`R' = NB_Int`m'_`R'/minTxeffect if NB_IntTyp`m'_`R'>-3

replace NB_IntRatio`m'_`R' = NB_Int`m'_`R'/txA if NB_IntTyp`m'_`R'<=-3 & NB_Int`m'_`R'/txA<0

replace NB_IntRatio`m'_`R' = NB_Int`m'_`R'/txB if NB_IntTyp`m'_`R'<=-3 & NB_Int`m'_`R'/txB<0

drop minTxeffect maxTxeffect txA txB

label define NB_IntTyp`m'_`R' -4 "Mixed Qualitative" -3 "Mixed Quant" -2 "Qualitative" -1 "SubAdd Quant" 0 "Zero" 1 "SuperAdd" 2 "LargeSuperAdd"

label values NB_IntTyp`m'_`R' NB_IntTyp`m'_`R'

egen MaxNBCurrent`m'_`R' = rowmax(NB_00`m'_`R' NB_A0`m'_`R' NB_0B`m'_`R' NB_AB`m'_`R')

generate BestTx`m'_`R' = 1 if NB_00`m'_`R'==MaxNBCurrent`m'_`R'

replace BestTx`m'_`R' = 2 if NB_A0`m'_`R'==MaxNBCurrent`m'_`R'

replace BestTx`m'_`R' = 3 if NB_0B`m'_`R'==MaxNBCurrent`m'_`R'

replace BestTx`m'_`R' = 4 if NB_AB`m'_`R'==MaxNBCurrent`m'_`R'

}

}

generate BestAnalysis_alwaysinclude= "cI_qI"

generate BestAnalysis_alwaysexclude= "cN_qN"

generate BestAnalysis_p0_05 = "cN_qN"

replace BestAnalysis_p0_05 = "cN_qI" if C_pvalueInt_cI_qI>=0.05 & Q_pvalueInt_cI_qI< 0.05

replace BestAnalysis_p0_05 = "cI_qN" if C_pvalueInt_cI_qI< 0.05 & Q_pvalueInt_cI_qI>=0.05

replace BestAnalysis_p0_05 = "cI_qI" if C_pvalueInt_cI_qI< 0.05 & Q_pvalueInt_cI_qI< 0.05

generate BestAnalysis_p0_10 = "cN_qN"

replace BestAnalysis_p0_10 = "cN_qI" if C_pvalueInt_cI_qI>=0.10 & Q_pvalueInt_cI_qI< 0.10

replace BestAnalysis_p0_10 = "cI_qN" if C_pvalueInt_cI_qI< 0.10 & Q_pvalueInt_cI_qI>=0.10

replace BestAnalysis_p0_10 = "cI_qI" if C_pvalueInt_cI_qI< 0.10 & Q_pvalueInt_cI_qI< 0.10

generate BestAnalysis_p0_25 = "cN_qN"

replace BestAnalysis_p0_25 = "cN_qI" if C_pvalueInt_cI_qI>=0.25 & Q_pvalueInt_cI_qI< 0.25

replace BestAnalysis_p0_25 = "cI_qN" if C_pvalueInt_cI_qI< 0.25 & Q_pvalueInt_cI_qI>=0.25

replace BestAnalysis_p0_25 = "cI_qI" if C_pvalueInt_cI_qI< 0.25 & Q_pvalueInt_cI_qI< 0.25

local ic = "aic bic"

foreach i of local ic {

generate BestAnalysis_`i' = "cN_qN"

replace BestAnalysis_`i' = "cN_qI" if `i'_cN_qI<`i'_cN_qN & `i'_cN_qI<`i'_cI_qN & `i'_cN_qI<`i'_cI_qI

replace BestAnalysis_`i' = "cI_qN" if `i'_cI_qN<`i'_cN_qN & `i'_cI_qN<`i'_cN_qI & `i'_cI_qN<`i'_cI_qI

replace BestAnalysis_`i' = "cI_qI" if `i'_cI_qI<`i'_cN_qN & `i'_cI_qI<`i'_cI_qN & `i'_cI_qI<`i'_cN_qI

generate BestAnalysis_`i'3 = "cN_qN"

replace BestAnalysis_`i'3 = "cN_qI" if `i'_cN_qI+3<`i'_cN_qN & `i'_cN_qI+3<`i'_cI_qN & `i'_cN_qI+3<`i'_cI_qI

replace BestAnalysis_`i'3 = "cI_qN" if `i'_cI_qN+3<`i'_cN_qN & `i'_cI_qN+3<`i'_cN_qI & `i'_cI_qN+3<`i'_cI_qI

replace BestAnalysis_`i'3 = "cI_qI" if `i'_cI_qI+3<`i'_cN_qN & `i'_cI_qI+3<`i'_cI_qN & `i'_cI_qI+3<`i'_cN_qI

}

generate BestAnalysis_R2 = "cN_qN"

replace BestAnalysis_R2 = "cN_qI" if R2Adj_cN_qI>R2Adj_cN_qN & R2Adj_cN_qI>R2Adj_cI_qN & R2Adj_cN_qI>R2Adj_cI_qI

replace BestAnalysis_R2 = "cI_qN" if R2Adj_cI_qN>R2Adj_cN_qN & R2Adj_cI_qN>R2Adj_cN_qI & R2Adj_cI_qN>R2Adj_cI_qI

replace BestAnalysis_R2 = "cI_qI" if R2Adj_cI_qI>R2Adj_cN_qN & R2Adj_cI_qI>R2Adj_cI_qN & R2Adj_cI_qI>R2Adj_cN_qI

generate BestAnalysis_qualitative = "cN_qN"

replace BestAnalysis_qualitative = "cN_qI" if C_IntTyp_cI_qI!=-2 & C_IntTyp_cI_qI!=-4 & (Q_IntTyp_cI_qI==-2 | Q_IntTyp_cI_qI==-4)

replace BestAnalysis_qualitative = "cI_qN" if (C_IntTyp_cI_qI==-2 | C_IntTyp_cI_qI==-4) & Q_IntTyp_cI_qI!=-2 & Q_IntTyp_cI_qI!=-4

replace BestAnalysis_qualitative = "cI_qI" if (C_IntTyp_cI_qI==-2 | C_IntTyp_cI_qI==-4) & (Q_IntTyp_cI_qI==-2 | Q_IntTyp_cI_qI==-4)

generate BestAnalysis_qual_large = "cN_qN"

replace BestAnalysis_qual_large = "cN_qI" if (C_IntTyp_cI_qI!=-2 & C_IntTyp_cI_qI!=2 & C_IntTyp_cI_qI!=-4) & (Q_IntTyp_cI_qI==-2 | Q_IntTyp_cI_qI==2 | Q_IntTyp_cI_qI==-4)

replace BestAnalysis_qual_large = "cI_qN" if (C_IntTyp_cI_qI==-2 | C_IntTyp_cI_qI==2 | C_IntTyp_cI_qI==-4) & (Q_IntTyp_cI_qI!=-2 & Q_IntTyp_cI_qI!=2 & Q_IntTyp_cI_qI!=-4)

replace BestAnalysis_qual_large = "cI_qI" if (C_IntTyp_cI_qI==-2 | C_IntTyp_cI_qI==2 | C_IntTyp_cI_qI==-4) & (Q_IntTyp_cI_qI==-2 | Q_IntTyp_cI_qI==2 | Q_IntTyp_cI_qI==-4)

generate BestAnalysis_qualitativeNB = "cN_qN"

replace BestAnalysis_qualitativeNB = "cN_qI" if (Q_IntTyp_cI_qI==-2 | NB_IntTyp_cN_qI_5000==-2 | NB_IntTyp_cN_qI_10000==-2 | NB_IntTyp_cN_qI_15000==-2 | NB_IntTyp_cN_qI_20000==-2 | NB_IntTyp_cN_qI_25000==-2 | NB_IntTyp_cN_qI_30000==-2 | NB_IntTyp_cN_qI_35000==-2 | NB_IntTyp_cN_qI_40000==-2 | Q_IntTyp_cI_qI==-4 | NB_IntTyp_cN_qI_5000==-4 | NB_IntTyp_cN_qI_10000==-4 | NB_IntTyp_cN_qI_15000==-4 | NB_IntTyp_cN_qI_20000==-4 | NB_IntTyp_cN_qI_25000==-4 | NB_IntTyp_cN_qI_30000==-4 | NB_IntTyp_cN_qI_35000==-4 | NB_IntTyp_cN_qI_40000==-4) & C_IntTyp_cI_qI!=-2 & NB_IntTyp_cI_qN_5000!=-2 & NB_IntTyp_cI_qN_10000!=-2 & NB_IntTyp_cI_qN_15000!=-2 & NB_IntTyp_cI_qN_20000!=-2 & NB_IntTyp_cI_qN_25000!=-2 & NB_IntTyp_cI_qN_30000!=-2 & NB_IntTyp_cI_qN_35000!=-2 & NB_IntTyp_cI_qN_40000!=-2 & C_IntTyp_cI_qI!=-4 & NB_IntTyp_cI_qN_5000!=-4 & NB_IntTyp_cI_qN_10000!=-4 & NB_IntTyp_cI_qN_15000!=-4 & NB_IntTyp_cI_qN_20000!=-4 & NB_IntTyp_cI_qN_25000!=-4 & NB_IntTyp_cI_qN_30000!=-4 & NB_IntTyp_cI_qN_35000!=-4 & NB_IntTyp_cI_qN_40000!=-4

replace BestAnalysis_qualitativeNB = "cI_qN" if (Q_IntTyp_cI_qI!=-2 & NB_IntTyp_cN_qI_5000!=-2 & NB_IntTyp_cN_qI_10000!=-2 & NB_IntTyp_cN_qI_15000!=-2 & NB_IntTyp_cN_qI_20000!=-2 & NB_IntTyp_cN_qI_25000!=-2 & NB_IntTyp_cN_qI_30000!=-2 & NB_IntTyp_cN_qI_35000!=-2 & NB_IntTyp_cN_qI_40000!=-2 & Q_IntTyp_cI_qI!=-4 & NB_IntTyp_cN_qI_5000!=-4 & NB_IntTyp_cN_qI_10000!=-4 & NB_IntTyp_cN_qI_15000!=-4 & NB_IntTyp_cN_qI_20000!=-4 & NB_IntTyp_cN_qI_25000!=-4 & NB_IntTyp_cN_qI_30000!=-4 & NB_IntTyp_cN_qI_35000!=-4 & NB_IntTyp_cN_qI_40000!=-4) & (C_IntTyp_cI_qI==-2 | NB_IntTyp_cI_qN_5000==-2 | NB_IntTyp_cI_qN_10000==-2 | NB_IntTyp_cI_qN_15000==-2 | NB_IntTyp_cI_qN_20000==-2 | NB_IntTyp_cI_qN_25000==-2 | NB_IntTyp_cI_qN_30000==-2 | NB_IntTyp_cI_qN_35000==-2 | NB_IntTyp_cI_qN_40000==-2 | C_IntTyp_cI_qI==-4 | NB_IntTyp_cI_qN_5000==-4 | NB_IntTyp_cI_qN_10000==-4 | NB_IntTyp_cI_qN_15000==-4 | NB_IntTyp_cI_qN_20000==-4 | NB_IntTyp_cI_qN_25000==-4 | NB_IntTyp_cI_qN_30000==-4 | NB_IntTyp_cI_qN_35000==-4 | NB_IntTyp_cI_qN_40000==-4)

replace BestAnalysis_qualitativeNB = "cI_qI" if (Q_IntTyp_cI_qI==-2 | NB_IntTyp_cN_qI_5000==-2 | NB_IntTyp_cN_qI_10000==-2 | NB_IntTyp_cN_qI_15000==-2 | NB_IntTyp_cN_qI_20000==-2 | NB_IntTyp_cN_qI_25000==-2 | NB_IntTyp_cN_qI_30000==-2 | NB_IntTyp_cN_qI_35000==-2 | NB_IntTyp_cN_qI_40000==-2 | Q_IntTyp_cI_qI==-4 | NB_IntTyp_cN_qI_5000==-4 | NB_IntTyp_cN_qI_10000==-4 | NB_IntTyp_cN_qI_15000==-4 | NB_IntTyp_cN_qI_20000==-4 | NB_IntTyp_cN_qI_25000==-4 | NB_IntTyp_cN_qI_30000==-4 | NB_IntTyp_cN_qI_35000==-4 | NB_IntTyp_cN_qI_40000==-4) & (C_IntTyp_cI_qI==-2 | NB_IntTyp_cI_qN_5000==-2 | NB_IntTyp_cI_qN_10000==-2 | NB_IntTyp_cI_qN_15000==-2 | NB_IntTyp_cI_qN_20000==-2 | NB_IntTyp_cI_qN_25000==-2 | NB_IntTyp_cI_qN_30000==-2 | NB_IntTyp_cI_qN_35000==-2 | NB_IntTyp_cI_qN_40000==-2 | C_IntTyp_cI_qI==-4 | NB_IntTyp_cI_qN_5000==-4 | NB_IntTyp_cI_qN_10000==-4 | NB_IntTyp_cI_qN_15000==-4 | NB_IntTyp_cI_qN_20000==-4 | NB_IntTyp_cI_qN_25000==-4 | NB_IntTyp_cI_qN_30000==-4 | NB_IntTyp_cI_qN_35000==-4 | NB_IntTyp_cI_qN_40000==-4)

generate BestAnalysis_qual_largeNB = "cN_qN"

replace BestAnalysis_qual_largeNB = "cN_qI" if (Q_IntTyp_cI_qI==-2 | NB_IntTyp_cN_qI_5000==-2 | NB_IntTyp_cN_qI_10000==-2 | NB_IntTyp_cN_qI_15000==-2 | NB_IntTyp_cN_qI_20000==-2 | NB_IntTyp_cN_qI_25000==-2 | NB_IntTyp_cN_qI_30000==-2 | NB_IntTyp_cN_qI_35000==-2 | NB_IntTyp_cN_qI_40000==-2 | Q_IntTyp_cI_qI==-4 | NB_IntTyp_cN_qI_5000==-4 | NB_IntTyp_cN_qI_10000==-4 | NB_IntTyp_cN_qI_15000==-4 | NB_IntTyp_cN_qI_20000==-4 | NB_IntTyp_cN_qI_25000==-4 | NB_IntTyp_cN_qI_30000==-4 | NB_IntTyp_cN_qI_35000==-4 | NB_IntTyp_cN_qI_40000==-4 | Q_IntTyp_cI_qI==2 | NB_IntTyp_cN_qI_5000==2 | NB_IntTyp_cN_qI_10000==2 | NB_IntTyp_cN_qI_15000==2 | NB_IntTyp_cN_qI_20000==2 | NB_IntTyp_cN_qI_25000==2 | NB_IntTyp_cN_qI_30000==2 | NB_IntTyp_cN_qI_35000==2 | NB_IntTyp_cN_qI_40000==2) & C_IntTyp_cI_qI!=-2 & NB_IntTyp_cI_qN_5000!=-2 & NB_IntTyp_cI_qN_10000!=-2 & NB_IntTyp_cI_qN_15000!=-2 & NB_IntTyp_cI_qN_20000!=-2 & NB_IntTyp_cI_qN_25000!=-2 & NB_IntTyp_cI_qN_30000!=-2 & NB_IntTyp_cI_qN_35000!=-2 & NB_IntTyp_cI_qN_40000!=-2 & C_IntTyp_cI_qI!=-4 & NB_IntTyp_cI_qN_5000!=-4 & NB_IntTyp_cI_qN_10000!=-4 & NB_IntTyp_cI_qN_15000!=-4 & NB_IntTyp_cI_qN_20000!=-4 & NB_IntTyp_cI_qN_25000!=-4 & NB_IntTyp_cI_qN_30000!=-4 & NB_IntTyp_cI_qN_35000!=-4 & NB_IntTyp_cI_qN_40000!=-4 & C_IntTyp_cI_qI!=2 & NB_IntTyp_cI_qN_5000!=2 & NB_IntTyp_cI_qN_10000!=2 & NB_IntTyp_cI_qN_15000!=2 & NB_IntTyp_cI_qN_20000!=2 & NB_IntTyp_cI_qN_25000!=2 & NB_IntTyp_cI_qN_30000!=2 & NB_IntTyp_cI_qN_35000!=2 & NB_IntTyp_cI_qN_40000!=2

replace BestAnalysis_qual_largeNB = "cI_qN" if (Q_IntTyp_cI_qI!=-2 & NB_IntTyp_cN_qI_5000!=-2 & NB_IntTyp_cN_qI_10000!=-2 & NB_IntTyp_cN_qI_15000!=-2 & NB_IntTyp_cN_qI_20000!=-2 & NB_IntTyp_cN_qI_25000!=-2 & NB_IntTyp_cN_qI_30000!=-2 & NB_IntTyp_cN_qI_35000!=-2 & NB_IntTyp_cN_qI_40000!=-2 & Q_IntTyp_cI_qI!=-4 & NB_IntTyp_cN_qI_5000!=-4 & NB_IntTyp_cN_qI_10000!=-4 & NB_IntTyp_cN_qI_15000!=-4 & NB_IntTyp_cN_qI_20000!=-4 & NB_IntTyp_cN_qI_25000!=-4 & NB_IntTyp_cN_qI_30000!=-4 & NB_IntTyp_cN_qI_35000!=-4 & NB_IntTyp_cN_qI_40000!=-4 & Q_IntTyp_cI_qI!=2 & NB_IntTyp_cN_qI_5000!=2 & NB_IntTyp_cN_qI_10000!=2 & NB_IntTyp_cN_qI_15000!=2 & NB_IntTyp_cN_qI_20000!=2 & NB_IntTyp_cN_qI_25000!=2 & NB_IntTyp_cN_qI_30000!=2 & NB_IntTyp_cN_qI_35000!=2 & NB_IntTyp_cN_qI_40000!=2) & (C_IntTyp_cI_qI==-2 | NB_IntTyp_cI_qN_5000==-2 | NB_IntTyp_cI_qN_10000==-2 | NB_IntTyp_cI_qN_15000==-2 | NB_IntTyp_cI_qN_20000==-2 | NB_IntTyp_cI_qN_25000==-2 | NB_IntTyp_cI_qN_30000==-2 | NB_IntTyp_cI_qN_35000==-2 | NB_IntTyp_cI_qN_40000==-2 | C_IntTyp_cI_qI==-4 | NB_IntTyp_cI_qN_5000==-4 | NB_IntTyp_cI_qN_10000==-4 | NB_IntTyp_cI_qN_15000==-4 | NB_IntTyp_cI_qN_20000==-4 | NB_IntTyp_cI_qN_25000==-4 | NB_IntTyp_cI_qN_30000==-4 | NB_IntTyp_cI_qN_35000==-4 | NB_IntTyp_cI_qN_40000==-4 | C_IntTyp_cI_qI==2 | NB_IntTyp_cI_qN_5000==2 | NB_IntTyp_cI_qN_10000==2 | NB_IntTyp_cI_qN_15000==2 | NB_IntTyp_cI_qN_20000==2 | NB_IntTyp_cI_qN_25000==2 | NB_IntTyp_cI_qN_30000==2 | NB_IntTyp_cI_qN_35000==2 | NB_IntTyp_cI_qN_40000==2)

replace BestAnalysis_qual_largeNB = "cI_qI" if (Q_IntTyp_cI_qI==-2 | NB_IntTyp_cN_qI_5000==-2 | NB_IntTyp_cN_qI_10000==-2 | NB_IntTyp_cN_qI_15000==-2 | NB_IntTyp_cN_qI_20000==-2 | NB_IntTyp_cN_qI_25000==-2 | NB_IntTyp_cN_qI_30000==-2 | NB_IntTyp_cN_qI_35000==-2 | NB_IntTyp_cN_qI_40000==-2 | Q_IntTyp_cI_qI==-4 | NB_IntTyp_cN_qI_5000==-4 | NB_IntTyp_cN_qI_10000==-4 | NB_IntTyp_cN_qI_15000==-4 | NB_IntTyp_cN_qI_20000==-4 | NB_IntTyp_cN_qI_25000==-4 | NB_IntTyp_cN_qI_30000==-4 | NB_IntTyp_cN_qI_35000==-4 | NB_IntTyp_cN_qI_40000==-4 | Q_IntTyp_cI_qI==2 | NB_IntTyp_cN_qI_5000==2 | NB_IntTyp_cN_qI_10000==2 | NB_IntTyp_cN_qI_15000==2 | NB_IntTyp_cN_qI_20000==2 | NB_IntTyp_cN_qI_25000==2 | NB_IntTyp_cN_qI_30000==2 | NB_IntTyp_cN_qI_35000==2 | NB_IntTyp_cN_qI_40000==2) & (C_IntTyp_cI_qI==-2 | NB_IntTyp_cI_qN_5000==-2 | NB_IntTyp_cI_qN_10000==-2 | NB_IntTyp_cI_qN_15000==-2 | NB_IntTyp_cI_qN_20000==-2 | NB_IntTyp_cI_qN_25000==-2 | NB_IntTyp_cI_qN_30000==-2 | NB_IntTyp_cI_qN_35000==-2 | NB_IntTyp_cI_qN_40000==-2 | C_IntTyp_cI_qI==-4 | NB_IntTyp_cI_qN_5000==-4 | NB_IntTyp_cI_qN_10000==-4 | NB_IntTyp_cI_qN_15000==-4 | NB_IntTyp_cI_qN_20000==-4 | NB_IntTyp_cI_qN_25000==-4 | NB_IntTyp_cI_qN_30000==-4 | NB_IntTyp_cI_qN_35000==-4 | NB_IntTyp_cI_qN_40000==-4 | C_IntTyp_cI_qI==2 | NB_IntTyp_cI_qN_5000==2 | NB_IntTyp_cI_qN_10000==2 | NB_IntTyp_cI_qN_15000==2 | NB_IntTyp_cI_qN_20000==2 | NB_IntTyp_cI_qN_25000==2 | NB_IntTyp_cI_qN_30000==2 | NB_IntTyp_cI_qN_35000==2 | NB_IntTyp_cI_qN_40000==2)

generate BestAnalysis_qual_largeSig = "cN_qN"

replace BestAnalysis_qual_largeSig = "cN_qI" if (C_IntTyp_cI_qI!=-2 & C_IntTyp_cI_qI!=2 & C_IntTyp_cI_qI!=-4 & C_pvalueInt_cI_qI>=0.05) & (Q_IntTyp_cI_qI==-2 | Q_IntTyp_cI_qI==2 | Q_IntTyp_cI_qI==-4 | Q_pvalueInt_cI_qI< 0.05)

replace BestAnalysis_qual_largeSig = "cI_qN" if (C_IntTyp_cI_qI==-2 | C_IntTyp_cI_qI==2 | C_IntTyp_cI_qI==-4 | C_pvalueInt_cI_qI< 0.05) & (Q_IntTyp_cI_qI!=-2 & Q_IntTyp_cI_qI!=2 & Q_IntTyp_cI_qI!=-4 & Q_pvalueInt_cI_qI>=0.05)

replace BestAnalysis_qual_largeSig = "cI_qI" if (C_IntTyp_cI_qI==-2 | C_IntTyp_cI_qI==2 | C_IntTyp_cI_qI==-4 | C_pvalueInt_cI_qI< 0.05) & (Q_IntTyp_cI_qI==-2 | Q_IntTyp_cI_qI==2 | Q_IntTyp_cI_qI==-4 | Q_pvalueInt_cI_qI< 0.05)

local size = "250 500 1000"

foreach s of local size {

generate BestAnalysis_`s' = "cN_qN"

replace BestAnalysis_`s' = "cN_qI" if abs(Q_Int_cI_qI)>=`s'/1000 & abs(C_Int_cI_qI)< `s'

replace BestAnalysis_`s' = "cI_qN" if abs(Q_Int_cI_qI)< `s'/1000 & abs(C_Int_cI_qI)>=`s'

replace BestAnalysis_`s' = "cI_qI" if abs(Q_Int_cI_qI)>=`s'/1000 & abs(C_Int_cI_qI)>=`s'

}

forvalues R=0(5000)40000 {

generate Correct_NB_00_`R' = (q_cons )*`R'-(c_cons)

generate Correct_NB_A0_`R' = (q_cons + q_A )*`R'-(c_cons + c_A)

generate Correct_NB_0B_`R' = (q_cons + q_B )*`R'-(c_cons + c_B)

generate Correct_NB_AB_`R' = (q_cons + q_A + q_B + q_Int)*`R'-(c_cons + c_A + c_B + c_Int)

generate Correct_NB_Int_`R'= Correct_NB_00_`R' + Correct_NB_AB_`R' - Correct_NB_A0_`R' - Correct_NB_0B_`R'

egen Correct_MaxNBCurrent_`R' = rowmax(Correct_NB_00_`R' Correct_NB_A0_`R' Correct_NB_0B_`R' Correct_NB_AB_`R')

generate Correct_BestTx_`R' = 1 if Correct_NB_00_`R'==Correct_MaxNBCurrent_`R'

replace Correct_BestTx_`R' = 2 if Correct_NB_A0_`R'==Correct_MaxNBCurrent_`R'

replace Correct_BestTx_`R' = 3 if Correct_NB_0B_`R'==Correct_MaxNBCurrent_`R'

replace Correct_BestTx_`R' = 4 if Correct_NB_AB_`R'==Correct_MaxNBCurrent_`R'

}

local criteria = "alwaysinclude alwaysexclude p0_05 p0_10 p0_25 aic bic aic3 bic3 R2 qualitative qual_large qualitativeNB qual_largeNB qual_largeSig 250 500 1000"

forvalues R=5000(15000)20000 {

foreach c of local criteria {

generate BestTx_`c'_`R' = BestTx_cN_qN_`R' if BestAnalysis_`c'=="cN_qN"

replace BestTx_`c'_`R' = BestTx_cI_qN_`R' if BestAnalysis_`c'=="cI_qN"

replace BestTx_`c'_`R' = BestTx_cN_qI_`R' if BestAnalysis_`c'=="cN_qI"

replace BestTx_`c'_`R' = BestTx_cI_qI_`R' if BestAnalysis_`c'=="cI_qI"

generate RightBestTx`c'_`R' = 0

replace RightBestTx`c'_`R' = 1 if BestTx_`c'_`R'==Correct_BestTx_`R'

generate netloss`c'_`R' = Correct_MaxNBCurrent_`R'-Correct_NB_00_`R' if BestTx_`c'_`R'==1

replace netloss`c'_`R' = Correct_MaxNBCurrent_`R'-Correct_NB_A0_`R' if BestTx_`c'_`R'==2

replace netloss`c'_`R' = Correct_MaxNBCurrent_`R'-Correct_NB_0B_`R' if BestTx_`c'_`R'==3

replace netloss`c'_`R' = Correct_MaxNBCurrent_`R'-Correct_NB_AB_`R' if BestTx_`c'_`R'==4

generate EffectA_`c'_`R' = NB_A0_cN_qN_`R'-NB_00_cN_qN_`R' if BestAnalysis_`c'=="cN_qN"

replace EffectA_`c'_`R' = NB_A0_cI_qN_`R'-NB_00_cI_qN_`R' if BestAnalysis_`c'=="cI_qN"

replace EffectA_`c'_`R' = NB_A0_cN_qI_`R'-NB_00_cN_qI_`R' if BestAnalysis_`c'=="cN_qI"

replace EffectA_`c'_`R' = NB_A0_cI_qI_`R'-NB_00_cI_qI_`R' if BestAnalysis_`c'=="cI_qI"

generate EffectB_`c'_`R' = NB_0B_cN_qN_`R'-NB_00_cN_qN_`R' if BestAnalysis_`c'=="cN_qN"

replace EffectB_`c'_`R' = NB_0B_cI_qN_`R'-NB_00_cI_qN_`R' if BestAnalysis_`c'=="cI_qN"

replace EffectB_`c'_`R' = NB_0B_cN_qI_`R'-NB_00_cN_qI_`R' if BestAnalysis_`c'=="cN_qI"

replace EffectB_`c'_`R' = NB_0B_cI_qI_`R'-NB_00_cI_qI_`R' if BestAnalysis_`c'=="cI_qI"

}

}

foreach c of local criteria {

generate RightBestTx`c'_Rc = RightBestTx`c'_20000 if (Dataset>=7 & Dataset<=18) | Dataset>=37 //ATEAM & UKBEAM, KAT, IVAN use QALYs

generate netloss`c'_Rc = netloss`c'_20000 if (Dataset>=7 & Dataset<=18) | Dataset>=37 //ATEAM & UKBEAM, KAT, IVAN use QALYs

replace RightBestTx`c'_Rc = RightBestTx`c'_5000 if (Dataset>=19 & Dataset<=36) | Dataset<=6 //Boyle, AFIST, MTA, Hollis use other outcomes

replace netloss`c'_Rc = netloss`c'_5000 if (Dataset>=19 & Dataset<=36) | Dataset<=6 //Boyle, AFIST, MTA, Hollis use other outcomes

}

generate Correct_BestTx_Rc = Correct_BestTx_20000 if (Dataset>=7 & Dataset<=18) | Dataset>=37 //ATEAM & UKBEAM, KAT, IVAN use QALYs

replace Correct_BestTx_Rc = Correct_BestTx_5000 if (Dataset>=19 & Dataset<=36) | Dataset<=6 //Boyle, AFIST, MTA, Hollis use other outcomes

sort Dataset run

//Identifying true int type & ratio

generate N_Int= Correct_NB_Int_20000 if (Dataset>=7 & Dataset<=18) | Dataset>=37 //ATEAM & UKBEAM, KAT, IVAN use QALYs

replace N_Int= Correct_NB_Int_5000 if (Dataset>=19 & Dataset<=36) | Dataset<=6 //Boyle, AFIST, MTA, Hollis use other outcomes

generate N_A = Correct_NB_A0_20000-Correct_NB_00_20000 if (Dataset>=7 & Dataset<=18) | Dataset>=37 //ATEAM & UKBEAM, KAT, IVAN use QALYs

generate N_B = Correct_NB_0B_20000-Correct_NB_00_20000 if (Dataset>=7 & Dataset<=18) | Dataset>=37 //ATEAM & UKBEAM, KAT, IVAN use QALYs

replace N_A = Correct_NB_A0_5000 -Correct_NB_00_5000 if (Dataset>=19 & Dataset<=36) | Dataset<=6 //Boyle, AFIST, MTA, Hollis use other outcomes

replace N_B = Correct_NB_0B_5000 -Correct_NB_00_5000 if (Dataset>=19 & Dataset<=36) | Dataset<=6 //Boyle, AFIST, MTA, Hollis use other outcomes

local outcome "q c N"

foreach o of local outcome {

egen minTxeffect = rowmin(`o'_A `o'_B)

egen maxTxeffect = rowmax(`o'_A `o'_B)

replace minTxeffect=maxTxeffect if abs(minTxeffect)>abs(maxTxeffect)

generate `o'_IntTyp = 0 if `o'_Int==0

replace `o'_IntTyp = -1 if (`o'_Int<0 & minTxeffect>=0) | (`o'_Int>0 & minTxeffect<=0) //Sub-additive

replace `o'_IntTyp = -2 if (`o'_Int<0 & minTxeffect>=0 & abs(`o'_Int)>abs(minTxeffect)) | (`o'_Int>0 & minTxeffect<=0 & abs(`o'_Int)>abs(minTxeffect)) //Qualitative

replace `o'_IntTyp = 1 if (`o'_Int>0 & minTxeffect> 0) | (`o'_Int<0 & minTxeffect< 0) //Super-additive

replace `o'_IntTyp = 2 if (`o'_Int>0 & minTxeffect> 0 & abs(`o'_Int)>abs(minTxeffect)) | (`o'_Int<0 & minTxeffect< 0 & abs(`o'_Int)>abs(minTxeffect)) //Super-additive & larger than main effect

replace `o'_IntTyp = -3 if `o'_A/`o'_B<0 & `o'_Int!=0 //Mixed interaction (could at this stage be qualitative or not)

replace `o'_IntTyp = -4 if `o'_A/`o'_B<0 & `o'_Int!=0 & ((`o'_Int/`o'_A<0 & abs(`o'_Int)>abs(`o'_A)) | (`o'_Int/`o'_B<0 & abs(`o'_Int)>abs(`o'_B))) //Mixed qualitative interaction, where minTxeffect is the one with the opposing sign

generate `o'_IntRatio = `o'_Int/minTxeffect if `o'_IntTyp>-3

replace `o'_IntRatio = `o'_Int/`o'_A if `o'_IntTyp<=-3 & `o'_Int/`o'_A<0

replace `o'_IntRatio = `o'_Int/`o'_B if `o'_IntTyp<=-3 & `o'_Int/`o'_B<0

drop minTxeffect maxTxeffect

label define `o'_IntTyp -4 "Mixed Qualitative" -3 "Mixed Quant" -2 "Qualitative" -1 "SubAdd Quant" 0 "Zero" 1 "SuperAdd" 2 "LargeSuperAdd"

label values `o'_IntTyp `o'_IntTyp

}

//Coverage and power

local model="_cN_qN _cI_qN _cN_qI _cI_qI"

foreach m of local model {

local treatment "A B"

foreach t of local treatment {

generate coverage_C`m'_`t' = 0

replace coverage_C`m'_`t' = 1 if c_`t' < C_`t'`m' + 1.959963985*C_`t'_SE`m' & c_`t' > C_`t'`m' - 1.959963985*C_`t'_SE`m' //Coverage is 1 for this run if true value is within the 95% CI: i.e. above the lower limit and below the upper limit

generate coverage_Q`m'_`t' = 0

replace coverage_Q`m'_`t' = 1 if q_`t' < Q_`t'`m' + 1.959963985*Q_`t'_SE`m' & q_`t' > Q_`t'`m' - 1.959963985*Q_`t'_SE`m'

}

}

local criteria = "alwaysinclude alwaysexclude p0_05 p0_10 p0_25 aic bic aic3 bic3 R2 qualitative qual_large qualitativeNB qual_largeNB qual_largeSig 250 500 1000"

foreach c of local criteria {

local treatment "A B"

foreach t of local treatment {

local outcome "C Q"

foreach o of local outcome {

generate coverage_`o'_`t'_`c' = coverage_`o'_cN_qN_`t' if BestAnalysis_`c'=="cN_qN"

replace coverage_`o'_`t'_`c' = coverage_`o'_cN_qI_`t' if BestAnalysis_`c'=="cN_qI"

replace coverage_`o'_`t'_`c' = coverage_`o'_cI_qN_`t' if BestAnalysis_`c'=="cI_qN"

replace coverage_`o'_`t'_`c' = coverage_`o'_cI_qI_`t' if BestAnalysis_`c'=="cI_qI"

generate power_`o'_`t'_`c' = 0

replace power_`o'_`t'_`c' = 1 if `o'_pvalue`t'_cN_qN<0.05 & BestAnalysis_`c'=="cN_qN" //Power is 1 for this run if p<0.05 for main effect of A

replace power_`o'_`t'_`c' = 1 if `o'_pvalue`t'_cN_qI<0.05 & BestAnalysis_`c'=="cN_qI"

replace power_`o'_`t'_`c' = 1 if `o'_pvalue`t'_cI_qN<0.05 & BestAnalysis_`c'=="cI_qN"

replace power_`o'_`t'_`c' = 1 if `o'_pvalue`t'_cI_qI<0.05 & BestAnalysis_`c'=="cI_qI"

}

}

}

**References**

1. Reddy P, Kalus JS, Caron MF, Horowitz S, Karapanos A, Coleman CI et al. Economic analysis of intravenous plus oral amiodarone, atrial septal pacing, and both strategies to prevent atrial fibrillation after open heart surgery. Pharmacotherapy. 2004;24(8):1013-9.

2. Hollinghurst S, Sharp D, Ballard K, Barnett J, Beattie A, Evans M et al. Randomised controlled trial of Alexander technique lessons, exercise, and massage (ATEAM) for chronic and recurrent back pain: economic evaluation. BMJ. 2008;337:a2656.

3. Boyle J, McCartney E, Forbes J, O'Hare A. A randomised controlled trial and economic evaluation of direct versus indirect and individual versus group modes of speech and language therapy for children with primary language impairment. Health Technol Assess. 2007;11(25):iii-iv, xi-xii, 1-139. doi:99/36/04 [pii].

4. Hollis JF, McAfee TA, Fellows JL, Zbikowski SM, Stark M, Riedlinger K. The effectiveness and cost effectiveness of telephone counselling and the nicotine patch in a state tobacco quitline. Tobacco Control. 2007;16 Supplement(1):i53-i9.

5. Foster EM, Jensen PS, Schlander M, Pelham WE, Jr., Hechtman L, Arnold LE et al. Treatment for ADHD: is more complex treatment cost-effective for more complex cases? Health Serv Res. 2007;42(1 Pt 1):165-82. doi:HESR599.

6. United Kingdom back pain exercise and manipulation (UK BEAM) randomised trial: cost effectiveness of physical treatments for back pain in primary care. BMJ. 2004;329(7479):1381. doi:bmj.38282.607859.AE.

7. Sullivan FM, Swan IR, Donnan PT, Morrison JM, Smith BH, McKinstry B et al. A randomised controlled trial of the use of aciclovir and/or prednisolone for the early treatment of Bell's palsy: the BELLS study. Health Technol Assess. 2009;13(47):iii-iv, ix-xi 1-130. doi:10.3310/hta13470.

8. Salize HJP, Merkel SD-R, Reinhard ID-M, Twardella DP, Mann KMD, Brenner HMDMPH. Cost-effective Primary Care-Based Strategies to Improve Smoking Cessation: More Value for Money. Archives of Internal Medicine. 2009;169(3):230-5.

9. Sevick MA, Miller GD, Loeser RF, Williamson JD, Messier SP. Cost-effectiveness of exercise and diet in overweight and obese adults with knee osteoarthritis. Med Sci Sports Exerc. 2009;41(6):1167-74. doi:10.1249/MSS.0b013e318197ece7.

10. Lindgren P, Buxton M, Kahan T, Poulter NR, Dahlof B, Sever PS et al. The lifetime cost effectiveness of amlodipine-based therapy plus atorvastatin compared with atenolol plus atorvastatin, amlodipine-based therapy alone and atenolol-based therapy alone: results from ASCOT1. Pharmacoeconomics. 2009;27(3):221-30. doi:5 [pii].

11. Cantor JC, Morisky DE, Green LW, Levine DM, Salkever DS. Cost-effectiveness of educational interventions to improve patient outcomes in blood pressure control. Prev Med. 1985;14(6):782-800.

12. Brandon TH, Meade CD, Herzog TA, Chirikos TN, Webb MS, Cantor AB. Efficacy and cost-effectiveness of a minimal intervention to prevent smoking relapse: dismantling the effects of amount of content versus contact. J Consult Clin Psychol. 2004;72(5):797-808. doi:2004-19094-007 [pii]

10.1037/0022-006X.72.5.797.

13. Barnett PG, Masson CL, Sorensen JL, Wong W, Hall S. Linking opioid-dependent hospital patients to drug treatment: health care use and costs 6 months after randomization. Addiction. 2006;101(12):1797-804.

14. Saillour-Glenisson F, Michel P, Daucourt V. [Medico-economic assessment of two methods for implementing thyroid testing guidelines]. Rev Epidemiol Sante Publique. 2005;53 Spec No 1:1S79-88. doi:MDOI-RESP-09-2005-53-HS1-0398-7620-101019-200505046 [pii].

15. Brown J, Welton NJ, Bankhead C, Richards SH, Roberts L, Tydeman C et al. A Bayesian approach to analysing the cost-effectiveness of two primary care interventions aimed at improving attendance for breast screening. Health Econ. 2006;15(5):435-45. doi:10.1002/hec.1077.

16. National Institute for Health and Care Excellence. Guide to the methods of technology appraisal 2013. 2013. <http://www.nice.org.uk/media/D45/1E/GuideToMethodsTechnologyAppraisal2013.pdf>. Accessed 17th May 2013.

1. For QALYs, the ceiling allowed for discounting and equalled 1/(1+r)^t^ per year of analysis. [↑](#footnote-ref-1)
2. This was the National Institute of Mental Health Multimodal Treatment study of children with ADHD (NIMH-MTA). [↑](#footnote-ref-2)
3. These comprised the Atrial Fibrillation Suppression Trial (AFIST) and the Alexander Technique, Exercise and Massage (ATEAM) trial. [↑](#footnote-ref-3)
