# Supplementary material for: Which interactions matter in economic evaluations? A systematic review and simulation study
Source: BMC Med Res Methodol. 2020 May 7;20:109. doi: 10.1186/s12874-020-00978-0 (PMC7203889; doi:10.1186/s12874-020-00978-0)
Supplement: Supplementary file 4 — Additional file 4. Additional results of the simulation study. Presents data on the performance of different criteria in different types of scenario and for each individual scenario of each trial, as well as data on bias and coverage. [file 12874_2020_978_MOESM4_ESM.docx]

**Additional file 4: Additional results of the simulation study**

**Table 4.1** Variation in the best performing criterion between trials and scenarios

| **Study Name** | **Interaction type for NMB*** | **Strategies with lowest opportunity cost†** | **Opportunity cost** | | | |
| --- | --- | --- | --- | --- | --- | --- |
|  |  |  | **Minimum** | **Always include** | **Never include** | **Include if >simple effect** |
| AFIST | MQual | Always, | £857 | £857 | £2,936 | £919 |
| AFIST_DoubleInt | MQual | Always, p<0.05, p<0.10, p<0.25, AIC, Qual, QualBig, QualNMB, QualBigNMB, QualBigSig, ≥250, ≥500, ≥1000, | £0 | £0 | £2,106 | £0 |
| AFIST_HalfInt | Mixed | Never, | £593 | £1,453 | £593 | £1,457 |
| AFIST_DoubleSize | MQual | Always, | £140 | £140 | £2,900 | £171 |
| AFIST_NoInt | Zero | Never, | £1,777 | £1,946 | £1,777 | £1,946 |
| AFIST_NoInt_DoubleSize | Zero | AIC, | £510 | £766 | £514 | £750 |
| ATEAM | Mixed | Never, BIC, ≥500, ≥1000, | £9 | £26 | £9 | £26 |
| ATEAM_DoubleInt | Mixed | Qual, QualBig, QualNMB, QualBigNMB, QualBigSig, | £2 | £5 | £19 | £2 |
| ATEAM_HalfInt | Mixed | Never, ≥500, ≥1000, | £6 | £25 | £6 | £21 |
| ATEAM_DoubleSize | Mixed | ALL | £0 | £0 | £0 | £0 |
| ATEAM_NoInt | Zero | Never, ≥250, ≥500, ≥1000, | £17 | £44 | £17 | £44 |
| ATEAM_NoInt_DoubleSize | Zero | Never, ≥250, ≥500, ≥1000, | £0 | £8 | £0 | £11 |
| Boyle | Qual | QualNMB, | £2,403 | £2,466 | £3,427 | £2,466 |
| Boyle_DoubleInt | Qual | Always, QualBig, QualBigNMB, QualBigSig, ≥250, | £1,796 | £1,796 | £5,802 | £1,796 |
| Boyle_HalfInt | Qual | Never, | £1,082 | £1,455 | £1,082 | £1,455 |
| Boyle_DoubleSize | Qual | QualNMB, | £1,319 | £1,501 | £3,007 | £1,523 |
| Boyle_NoInt | Zero | Never, | £1,397 | £2,031 | £1,397 | £2,044 |
| Boyle_NoInt_DoubleSize | Zero | Never, | £840 | £1,388 | £840 | £1,362 |
| Hollis | Super | QualBigSig, | £13 | £20 | £14 | £22 |
| Hollis_DoubleInt | LargeSuper | Qual, QualBig, | £3 | £8 | £4 | £15 |
| Hollis_HalfInt | Super | Never, ≥250, ≥500, ≥1000, | £9 | £19 | £9 | £20 |
| Hollis_DoubleSize | Super | QualBig, | £3 | £7 | £4 | £8 |
| Hollis_NoInt | Zero | QualBig, | £13 | £21 | £14 | £20 |
| Hollis_NoInt_DoubleSize | Zero | Never, ≥250, ≥500, ≥1000, | £4 | £13 | £4 | £11 |
| MTA | MQual | ≥250, | £138 | £144 | £372 | £149 |
| MTA_DoubleInt | MQual | Always, QualBig, QualBigNMB, QualBigSig, | £138 | £138 | £942 | £138 |
| MTA_HalfInt | Mixed | p<0.10, AIC, | £101 | £117 | £144 | £128 |
| MTA_DoubleSize | MQual | Always, QualBigNMB, QualBigSig, | £43 | £43 | £170 | £43 |
| MTA_NoInt | Zero | Never, ≥1000, | £53 | £133 | £53 | £117 |
| MTA_NoInt_DoubleSize | Zero | Never, ≥1000, | £0 | £48 | £0 | £48 |
| UKBEAM | Qual | QualNMB, QualBigNMB, | £51 | £52 | £56 | £51 |
| UKBEAM_DoubleInt | Qual | QualNMB, QualBigNMB, | £102 | £105 | £170 | £102 |
| UKBEAM_HalfInt | Sub | Never, ≥250, ≥500, ≥1000, | £22 | £50 | £22 | £50 |
| UKBEAM_DoubleSize | Qual | QualNMB, QualBigNMB, | £43 | £44 | £63 | £43 |
| UKBEAM_NoInt | Zero | Qual, | £51 | £73 | £52 | £73 |
| UKBEAM_NoInt_DoubleSize | Zero | Never, ≥250, ≥500, ≥1000, | £35 | £52 | £35 | £52 |

* Abbreviations for interaction types: LargeSuper, super-additive interaction larger than the smaller of the two simple effects; Mixed, mixed non-qualitative interaction; MQual, mixed qualitative interaction; Qual, non-mixed qualitative interaction; SubAdd, sub-additive interaction; Super, super-additive interaction smaller than either the simple effect; Zero, interaction equals zero.

† Abbreviations for analyses: Always, always include all interactions [criterion 1]; Never, never include any interactions [criterion 2]; p<0.05 [or 0.10, 0.25], including interactions where p<0.05 [criterion 3], p<0.10 [criterion 4]; or p<0.25 [criterion 5]; AIC, include interactions decreasing AIC [criterion 6]; BIC, include interactions decreasing BIC [criterion 7]; Qual, include qualitative interactions in costs or benefits [criterion 8]; QualBig, include interactions for costs or benefits if >simple effect [criterion 9]; QualBigSig, include interactions for costs or benefits if p<0.05 or >simple effect [criterion 10]; QualNMB, include qualitative interactions for costs, benefits or NMB [criterion 11]; QualBigNMB, include interactions for costs, benefits or NMB if >simple effect [criterion 12]; ≥250, include |interactions| ≥0.25 or ≥£250 [criterion 13]; ≥500, include |interactions| ≥0.5 or ≥£500 [criterion 14]; ≥1000, include |interactions| ≥1 or ≥£250 [criterion 15]

We also assessed coverage, statistical power and bias (Table 4.2). Averaged across all trials, coverage was below 95% for all criteria other than “always include” (Table 4.3). However, some trials had a >95% coverage; the explanation for this is unclear, although it could result from the gamma-distributed costs and the ceiling effect for benefits.

**Table 4.2** The measures used to assess performance of the criteria for deciding which interactions are considered

| **Measure** | **Rationale** | **Details of calculation** |
| --- | --- | --- |
| Coverage for simple effects | Under a purely frequentist framework, the priority is to maximise the statistical power to detect the effect of A and B while keeping the risk of a type I error at alpha.  Coverage and power assess the performance of the criterion for hypothesis testing. However, this is less relevant for economic evaluation. Nonetheless, coverage will (to some extent) identify analyses that are prone to bias as well as those that are inefficient. | The proportion of simulations in which the 95% CI around the effect of A or B from the mixed model favoured by any given criterion included the true simple effect value that was used to generate the simulated trials. The simple effect (i.e. $\mu_{a}-\mu_{o}$) was used to estimate coverage in all cases, since an analysis that excludes an interaction implicitly assumes that the at-the-margin estimate $0.5(\mu_{a}+\mu_{ab}-\mu_{0}-\mu_{b})$ equals the simple effect $\mu_{a}-\mu_{o}$. |
| Statistical power for main effects |  | The proportion of simulations in which the coefficient of A (or B) in the analysis chosen by any given criterion is significantly different from zero (p<0.05). |
| Amount of bias in incremental effects | The treatment effects estimated within analyses excluding interactions are biased estimates of the simple effect ($\mu_{a}-\mu_{o}$), where the bias is half the size of the interaction term [1]. However, large interactions may have no impact on our treatment adoption decision, whereas some small interactions may change which treatment has highest NMB. | The average bias within estimates of the incremental costs [benefits] of A or B was estimated by multiplying the proportion of samples in which the analysis ignored interactions that were not truly zero by 50% of the absolute true interaction term within those scenarios. For example, if the true interaction were £200 and criterion X allowed for the interaction in 40% of samples:$bias=£40=\left\vert£200\right\vert\cdot50\%\cdot40\%$. |

In order to approximate the QALY distributions seen in real trials, health benefits for all scenarios other than those assuming no interaction were truncated at a maximum level relevant to each trial. Such ceiling effects reduce the means and standard deviations (SDs) for the simulated samples to values less than the secondary data on which they are based and reduce means/SDs more for the treatment arms with highest mean benefits. Such ceiling effects will not change which treatment has highest health benefits or NMB in each sample and will therefore not bias estimates of the probability or opportunity cost of adopting the wrong treatment. However, ceiling effects will mean that the average size of the interaction term across all 300 samples will be systematically different from the values shown in Online Resource 3. As result, mixed models estimated that the interactions for QALYs were, on average 9.8% lower than the data inputs would suggest; as expected, the discrepancy was greatest for the 20 scenarios where the ceiling effect was <2 SDs from the largest group mean. Ceiling effects will also introduce a systematic error into coverage estimates. Nonetheless, this is unlikely to affect the conclusions since ceiling effects were not used for the scenarios assuming no interaction.

Coverage was markedly reduced for criteria that excluded some/all interactions, since it was defined as the proportion of samples in which the 95% CI included the true *simple effect* (i.e. the difference between the means for groups *a* and *0*). For analyses excluding interactions, treatment effects are estimated as the effect of A averaged *across patients with and without B*; the 95% CI around this *main effect* may exclude the true simple effect due to either omitted-interaction bias or a failure of distributional assumptions. As result, coverage is affected by both bias and inefficiency and was directly proportional to the proportion of samples in which interactions were included.

On average, the six original trials had 76% (range: 39-100%) power to detect the main treatment effect for cost ignoring all interactions, compared with 49% (range: 24-82%) power for benefits. Although power was, on average, lower for "always include" than for "never include", some criteria (e.g. 8, 14 or 15) had higher power than "never include" (Table 4.2) because pooling treatment arms despite large interactions reduces power [2-5].

Bias from omitted interactions was substantial for many trials and criteria, but was completely avoided by including all interactions (Table 4.2). As expected, all other criteria produced some bias, although bias was 15-47% larger for "never include" than the second worst criterion (BIC). With the exception of “always include”, the three criteria including interactions above a certain absolute size had lowest absolute mean bias.

**Table 4.3** Comparison of performance of the different criteria with regards coverage, power and bias. The values shown in bold represent the most favourable of all criteria for this measure

| **Criterion** | **Coverage for effects of A and B:**  **% (n)^a^** | | **Power for effects of A and B:**  **% (n)**^b^ | | **Bias (0.5*ignored interactions):**  **mean (range across scenarios)**^c^ | |
| --- | --- | --- | --- | --- | --- | --- |
|  | **Cost** | **Benefit** | **Cost** | **Benefit** | **Cost** | **Benefit** |
| 1: Always include all interactions | **95.15% (20,553)** | **94.56% (20,424)** | 67.93% (14,673) | 43.95% (9,494) | **£0 (£0, £0)** | **0.00 (0.00, 0.00)** |
| 2: Never include any interactions | 57.70% (12,464) | 85.51% (18,470) | 76.09% (16,436) | 55.09% (11,900) | £770 (£0, £10,870) | 0.15 (0.00, 2.04) |
| 3: Include interactions where p<0.05 | 89.60% (19,353) | 87.89% (18,985) | 71.78% (15,504) | 54.42% (11,755) | £346 (£0, £3,587) | 0.10 (0.00, 1.11) |
| 4: Include interactions where p<0.10 | 91.02% (19,661) | 88.97% (19,218) | 71.31% (15,404) | 53.20% (11,491) | £256 (£0, £2,808) | 0.08 (0.00, 0.78) |
| 5: Include interactions where p<0.25 | 93.23% (20,138) | 91.45% (19,753) | 70.06% (15,133) | 49.93% (10,784) | £123 (£0, £1,522) | 0.04 (0.00, 0.38) |
| 6:Include interactions decreasing AIC | 90.49% (19,546) | 88.51% (19,119) | 71.66% (15,478) | 53.82% (11,626) | £302 (£0, £3,189) | 0.09 (0.00, 0.90) |
| 7: Include interactions decreasing BIC | 83.92% (18,127) | 86.30% (18,640) | 71.94% (15,540) | 55.28% (11,941) | £525 (£0, £6,377) | 0.13 (0.00, 1.54) |
| 8: Include qualitative interactions in cost or benefits | 72.87% (15,739) | 89.08% (19,242) | **77.66% (16,775)** | 54.77% (11,830) | £227 (£0, £2,935) | 0.04 (0.00, 0.32) |
| 9: Include interactions for cost or benefits if >simple effect | 84.92% (18,343) | 90.89% (19,632) | 70.03% (15,127) | 50.91% (10,997) | £108 (£0, £1,214) | 0.04 (0.00, 0.30) |
| 10: Include interactions for cost or benefits if p<0.05 or >simple effect | 91.82% (19,833) | 91.02% (19,660) | 69.95% (15,109) | 50.02% (10,805) | £104 (£0, £1,196) | 0.04 (0.00, 0.30) |
| 11: Include qualitative interactions for cost, benefits or NMB | 76.69% (16,564) | 90.79% (19,611) | 75.60% (16,330) | 52.07% (11,248) | £190 (£0, £2,935) | 0.03 (0.00, 0.21) |
| 12: Include interactions for cost, benefit or NMB if >simple effect | 86.12% (18,602) | 92.12% (19,898) | 69.73% (15,061) | 49.15% (10,616) | £89 (£0, £933) | 0.02 (0.00, 0.20) |
| 13: Include interactions ≥0.25 or ≥£250 | 76.05% (16,426) | 87.41% (18,880) | 69.82% (15,082) | 54.81% (11,840) | £24 (£0, £79) | 0.02 (0.00, 0.07) |
| 14: Include interactions ≥0.5 or ≥£500 | 68.18% (14,726) | 86.63% (18,711) | 69.76% (15,069) | **56.09% (12,116)** | £46 (£0, £180) | 0.03 (0.00, 0.13) |
| 15: Include interactions ≥1 or ≥£1,000 | 64.06% (13,836) | 86.50% (18,684) | 71.44% (15,431) | 55.93% (12,080) | £74 (£0, £303) | 0.04 (0.00, 0.23) |

^a^ Coverage = proportion of simulations in which the 95% CI around the effect of A or B from the mixed model favoured by any given criterion included the true simple effect value that was used to generate the simulated trials. Coverage is shown over the effects of A and B combined and is out of 32,400 (two treatment effects for each of 300 samples of 54 scenarios).

^b^ Power = proportion of simulations in which the coefficient of A (or B) in the analysis chosen by any given criterion is significantly different from zero (p<0.05). Power is shown over the effects of A and B combined and is out of 32,400 (two treatment effects for each of 300 samples of 54 scenarios).

^c^ Average bias within the incremental costs [benefits] = the proportion of samples in which the analysis ignored interactions that were not truly zero, multiplied by 50% of the absolute true interaction term within those scenarios. For example, if the true interaction were £200 and criterion X allowed for the interaction in 40% of samples, $bias=£40=\left|£200\right|\cdot50\%\cdot40\%$.

**Table 4.4** Comparison of performance of the different criteria with regards specificity and sensitivity to detect interactions. The values shown in bold represent the most favourable of all criteria for this measure

| **Criterion** | **Proportion of samples in which interactions are included: % (n)^a^** | | **Sensitivity: proportion of any non-zero interactions taken into account: % (n)^b^** | | **Specificity: proportion of interactions equal to 0 that are excluded: % (n)^c^** | |
| --- | --- | --- | --- | --- | --- | --- |
|  | **Cost** | **Benefit** | **Cost** | **Benefit** | **Cost** | **Benefit** |
| 1: Always include all interactions | **100.00% (10,800)** | **100.00% (10,800)** | **100.00% (7,200)** | **100.00% (7,200)** | 0.00% (0) | 0.00% (0) |
| 2: Never include any interactions | 0.00% (0) | 0.00% (0) | 0.00% (0) | 0.00% (0) | **100.00% (3,600)** | **100.00% (3,600)** |
| 3: Include interactions where p<0.05 | 47.68% (5,149) | 21.20% (2,290) | 66.49% (4,787) | 26.69% (1,922) | 89.94% (3,238) | 89.78% (3,232) |
| 4: Include interactions where p<0.10 | 56.58% (6,111) | 33.13% (3,578) | 74.69% (5,378) | 39.04% (2,811) | 79.64% (2,867) | 78.69% (2,833) |
| 5: Include interactions where p<0.25 | 75.50% (8,154) | 61.40% (6,631) | 88.03% (6,338) | 66.33% (4,776) | 49.56% (1,784) | 48.47% (1,745) |
| 6:Include interactions decreasing AIC | 53.10% (5,735) | 28.38% (3,065) | 71.67% (5,160) | 34.15% (2,459) | 84.03% (3,025) | 83.17% (2,994) |
| 7: Include interactions decreasing BIC | 32.56% (3,517) | 7.02% (758) | 48.10% (3,463) | 9.65% (695) | 98.50% (3,546) | 98.25% (3,537) |
| 8: Include qualitative interactions in cost or benefits | 37.64% (4,065) | 46.40% (5,011) | 44.36% (3,194) | 50.72% (3,652) | 75.81% (2,729) | 62.25% (2,241) |
| 9: Include interactions for cost or benefits if >simple effect | 55.56% (6,001) | 56.51% (6,103) | 66.79% (4,809) | 60.75% (4,374) | 66.89% (2,408) | 51.97% (1,871) |
| 10: Include interactions for cost or benefits if p<0.05 or >simple effect | 65.24% (7,046) | 58.31% (6,298) | 79.94% (5,756) | 62.90% (4,529) | 64.17% (2,310) | 50.86% (1,831) |
| 11: Include qualitative interactions for cost, benefits or NMB | 49.65% (5,362) | 64.37% (6,952) | 54.83% (3,948) | 66.68% (4,801) | 60.72% (2,186) | 40.25% (1,449) |
| 12: Include interactions for cost, benefit or NMB if >simple effect | 64.26% (6,940) | 71.44% (7,716) | 72.68% (5,233) | 74.11% (5,336) | 52.58% (1,893) | 33.89% (1,220) |
| 13: Include interactions ≥0.25 or ≥£250 | 38.33% (4,140) | 25.50% (2,754) | 46.54% (3,351) | 27.50% (1,980) | 78.08% (2,811) | 78.50% (2,826) |
| 14: Include interactions ≥0.5 or ≥£500 | 27.00% (2,916) | 16.46% (1,778) | 31.88% (2,295) | 17.50% (1,260) | 82.75% (2,979) | 85.61% (3,082) |
| 15: Include interactions ≥1 or ≥£1,000 | 20.80% (2,246) | 12.50% (1,350) | 23.57% (1,697) | 13.46% (969) | 84.75% (3,051) | 89.42% (3,219) |

^a^ Percentages are out of 10,800 (300 samples of 6 trials, each with six scenarios).

^b^ Percentages are out of 7,200 (300 samples of 6 trials, each with four scenarios with non-zero interactions).

^c^ Percentages are out of 5,400 (300 samples of 6 trials, each with two scenarios with zero interactions).

**Table 4.5** Comparison of performance of the different criteria with regards the probability and the opportunity cost associated with adopting a treatment that does not have highest true NMB. The values shown in bold represent the most favourable of all criteria for this measure

| **Criterion** | **Opportunity cost of adopting a suboptimal treatment** | | | | | **Probability of adopting treatment with highest NMB** | | | | |
| --- | --- | --- | --- | --- | --- | --- | --- | --- | --- | --- |
|  | **All datasets** | **6 original studies only** | **Zero interaction** | **Non-zero non-qualitative interaction** | **Qualitative interaction** | **All datasets** | **6 original studies only** | **Zero interaction** | **Non-zero non-qualitative interaction** | **Qualitative interaction** |
| Number of datasets included | **36** | **6** | **12** | **11** | **13** | **36** | **6** | **12** | **11** | **13** |
| 1: Always include all interactions | £472 | **£594** | £544 | £157 | **£672** | 81.70% | 80.83% | 79.75% | 85.48% | **80.31%** |
| 2: Never include any interactions | £793 | £1,136 | **£392** | **£75** | £1,772 | 76.60% | 67.72% | **86.14%** | **90.88%** | 55.72% |
| 3: Include interactions where p<0.05 | £556 | £846 | £429 | £96 | £1,062 | 80.61% | 74.61% | 84.08% | 89.36% | 70.00% |
| 4: Include interactions where p<0.10 | £515 | £759 | £468 | £121 | £893 | 81.32% | 77.11% | 82.33% | 88.27% | 74.51% |
| 5: Include interactions where p<0.25 | £491 | £661 | £523 | £154 | £747 | 81.58% | 79.50% | 80.42% | 86.03% | 78.90% |
| 6:Include interactions decreasing AIC | £529 | £791 | £454 | £102 | £960 | 81.11% | 75.89% | 83.00% | 88.91% | 72.77% |
| 7: Include interactions decreasing BIC | £646 | £1,014 | £405 | £81 | £1,347 | 78.32% | 70.50% | 85.64% | 90.27% | 61.46% |
| 8: Include qualitative interactions in cost or benefits | £503 | £660 | £523 | £154 | £779 | 81.71% | 79.50% | 82.89% | 87.52% | 75.72% |
| 9: Include interactions for cost or benefits if >simple effect | £474 | £633 | £517 | £152 | £708 | **83.31%** | 81.06% | 83.22% | 87.91% | 79.51% |
| 10: Include interactions for cost or benefits if p<0.05 or >simple effect | £475 | £632 | £521 | £152 | £707 | 83.14% | **81.22%** | 82.75% | 87.82% | 79.54% |
| 11: Include qualitative interactions for cost, benefits or NMB | £480 | £617 | £542 | £160 | £694 | 81.21% | 80.00% | 80.03% | 84.48% | 79.54% |
| 12: Include interactions for cost, benefit or NMB if >simple effect | £475 | £606 | £540 | £159 | £681 | 81.55% | 80.33% | 80.08% | 84.67% | 80.26% |
| 13: Include interactions ≥0.25 or ≥£250 | **£472** | £600 | £529 | £137 | £703 | 81.22% | 78.78% | 83.50% | 89.42% | 72.18% |
| 14: Include interactions ≥0.5 or ≥£500 | £476 | £638 | £531 | £132 | £718 | 82.33% | 79.11% | 83.69% | 89.85% | 74.72% |
| 15: Include interactions ≥1 or ≥£1,000 | £488 | £646 | £517 | £135 | £759 | 82.01% | 78.06% | 84.14% | 89.64% | 73.59% |

**References**

1. Hung HM. Two-stage tests for studying monotherapy and combination therapy in two-by-two factorial trials. Stat Med 1993, 12(7):645-660.

2. Brittain E, Wittes J. Factorial designs in clinical trials: the effects of non-compliance and subadditivity. Stat Med 1989, 8(2):161-171.

3. Slud EV. Analysis of factorial survival experiments. Biometrics 1994, 50(1):25-38.

4. Simon R, Freedman LS. Bayesian design and analysis of two x two factorial clinical trials. Biometrics 1997, 53(2):456-464.

5. Ng T. The impact of a preliminary test for interaction in a 2 x 2 factorial trial. Proceedings of the Biopharmaceutical Section of the American Statistical Association, Alexandria, VA 1991:220-227.
